# Design and synthesis of phenylthiophosphoryl dichloride derivatives and evaluation of their antitumour and anti-inflammatory activities

**DOI:** 10.3389/fchem.2024.1529211

**Published:** 2025-01-21

**Authors:** Chunyun Xu, Na Yang, Haichun Yu, Xiaojing Wang

**Affiliations:** Department of Dermatology, Maternity and Child Health Hospital of Qinhuangdao, Qinhuangdao, China

**Keywords:** hydrogen sulfide, network pharmacology, anti-tumor, anti-inflammatory, phenylthiophosphoryl dichloride

## Abstract

Tumours and inflammation are serious risks to human health and are importantly regulated by the gas signalling molecule hydrogen sulphide. In this work, we report the rational design and synthesis of H_2_S donor molecules based on phenylthiophosphoryl dichloride nuclei and assess their efficacy against tumours and inflammation. We predicted its potential anticancer targets based on network pharmacology and then verified the inhibitory effect of the active compound **S11** on the pathway PI3K/AKT by enzyme inhibition and molecular docking assay. In addition, compound S11 exhibited a potent anti-inflammatory effect on macrophages, effectively reducing the levels of inflammatory mediators TNF-α, IL-10 and HO-1. Compound **S11** can be used as a new chemical entity for the discovery of new anti-cancer drugs or anti-inflammatory drugs.

## Graphical Abstract

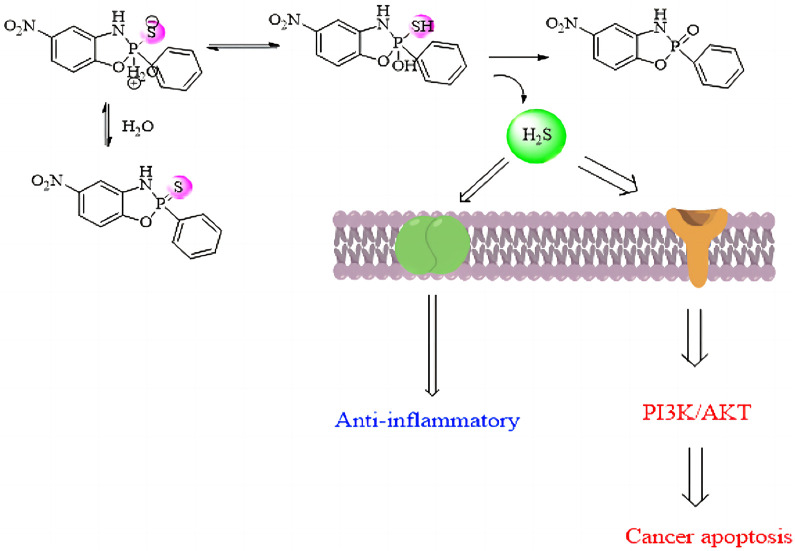

## 1 Introduction

**FIGURE 1:**
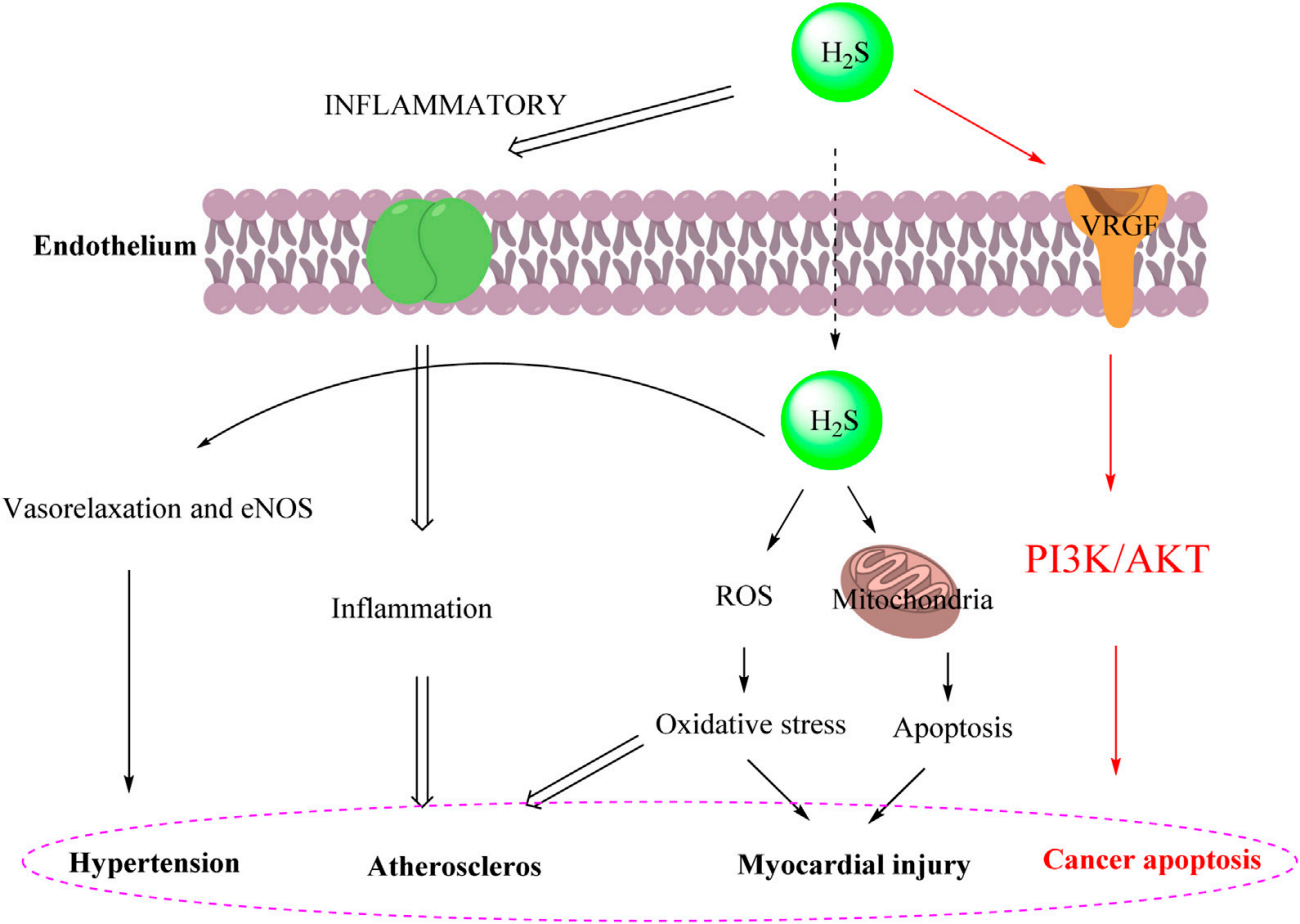
Schematic diagram of H_2_S partial immunomodulatory mechanism.

Hydrogen sulfide (H_2_S) is recognized as one of the three major gas signal transmitters along with nitric oxide (NO) and carbon monoxide (CO). In the past decade, H_2_S has been shown to have various biological activities, including positive regulatory effects in antioxidant mechanisms, promotion of angiogenesis, anti-inflammatory responses, and ion channel modulation (Hao et al., 2021; Li et al., 2021; Dilek et al., 2020). Furthermore, the cardioprotective effect of H₂S is associated with the inhibition of myocardial cell apoptosis following myocardial injury. The antioxidant effect of H_2_S is also reflected in preserving mitochondrial function by inhibiting mitochondrial respiration (Sanchez-Aranguren et al., 2020). Moreover, H_2_S plays a significant role in the hypertension (Goor et al., 2020), atheroscleros (Munteanu, 2023), myocardial injury (Kolluru et al., 2023) and cancer apoptosis (Gao et al., 2024) as illustrated in Figure 1.

Cancer can proliferate indefinitely by maintaining reproductive signalling or overexpressing growth factors to regenerate itself. It may also be initiated by aberrant activation of downstream signalling pathways, particularly the phosphatidylinositol 3-kinase (PI3K)/mammalian target of rapamycin (mTOR) pathway, or by aberrant inactivation of suppressor genes (Siegel et al., 2018; Moses et al., 2018). The critical role of the PI3K/AKT/mTOR (PAM) pathway in cell survival, proliferation, growth, and apoptosis influences make it a promising drug target in the war against cancer (Sanchez et al., 2019; Khan et al., 2019). Because H₂S gas is an important gas signalling transmitter, we can design a series of H₂S donors that can target and kill cancer cells by modulating the PI3K/AKT/mTOR pathway. Traditional drug discovery models can only verify the relationship between compounds and targets through a single experiment. In contrast, network pharmacology provides a “compound-protein/gene-disease” network approach, which operates on the principle of “network target, multi-component therapy” (Zhang et al., 2019). This makes it possible to analyse how small molecule drugs modulate disease at high throughput. Cyberpharmacology maps multi-drug ecological networks onto human and animal disease gene networks, providing a basis for identifying key disease-related drug targets (Nogales et al., 2022). In this study, a protein-protein interaction (PPI) network of compounds **S11** associated with cancer was constructed using publicly available databases. Subsequently, potential targets of **S11** for cancer treatment were identified. These targets were then analysed by Gene Ontology (GO) and Kyoto Encyclopedia of Genes and Genomes (KEGG) enrichment. Finally, it was validated using molecular docking methods with enzyme inhibition experiments. Similarly there is interest in the use of H_2_S donors for the treatment of inflammatory diseases. Therefore, it is of great interest to know as much as possible about H_2_S donors in vitro inflammatory systems. The anti-inflammatory effects of this series of hydrogen sulphide donors on macrophages *in vitro* were further tested in this context.

In a word, developing more effective organic H_2_S donors is of great significance for cancer and treating inflammation. This article designs a series of novel H_2_S donor molecules based on a sulfur-phosphorus core and the anti-tumor and anti-inflammatory mechanism is preliminarily explored, laying the foundation for the future design of H_2_S donor-based anti-inflammatory and anticancer drugs.

## 2 Results and discussion

### 2.1 Chemical synthesis

**Scheme 1:**
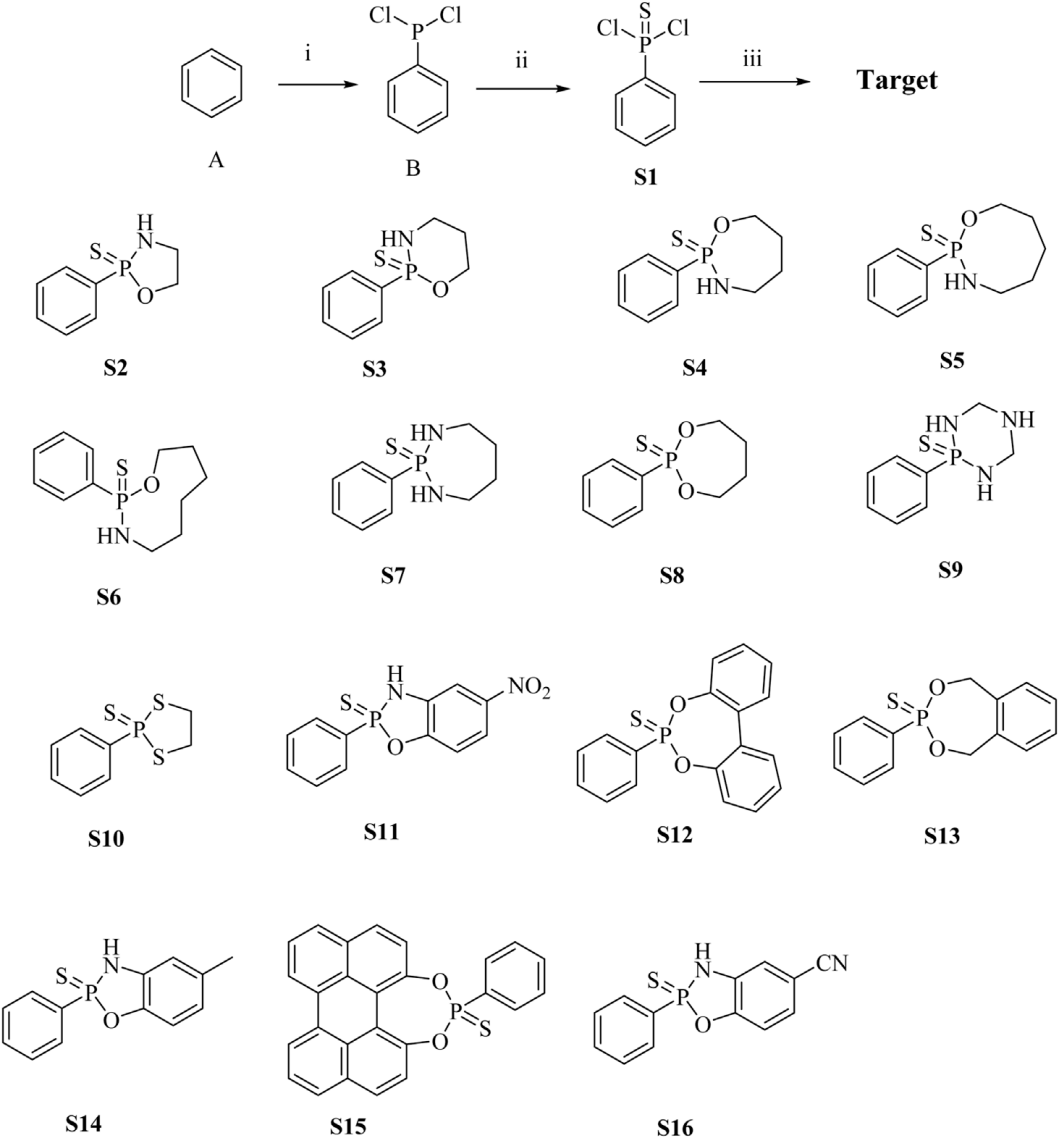
Synthesis of H_2_S donor **S1 - S16**. Conditions and reagents: (i) AlCl_3_, PCl_3_, yield 78%; (ii) S, Benzene, yield 45%; (iii) Triethylamine, different amino alcohols, diols or diamines, yield 30%–45%.

The main donor **S1** of H_2_S was efficiently and concisely synthesized using benzene as the starting material. Compound **S2**-**S16** were synthesized by reacting dichlorophenylthiophosphoryl with different amino alcohols, diols, or diamines in the presence of triethylamine (Scheme 1). In the ^1^H NMR spectra of compounds **S1**-**S16**, the chemical shifts of hydrogen atoms in benzene ring appeared in the range of 8.88–7.28 ppm. For most compounds, the signals of protons on OCH_2_ appeared in the range of 3.40–4.49 ppm. For compounds containing NH group, the chemical shifts of hydrogen in NH are in the range of 3.05–3.54 ppm. Our NMR data for compounds **S2**-**S4** in this paper are consistent with the NMR data reported in Zhang’s article (Zhang et al., 2019).

### 2.2 H_2_S release ability of the compound and its influencing factors

**FIGURE 2:**
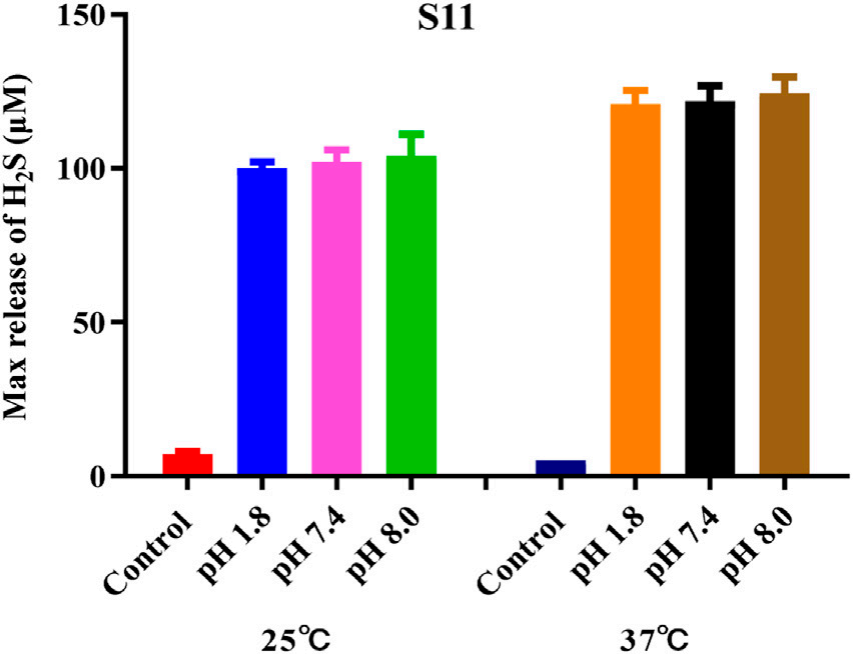
Amount of H_2_S released by compound **S11**. Each bar represents the mean ± SD (standard deviation) of three independent experiments.

**FIGURE 3:**
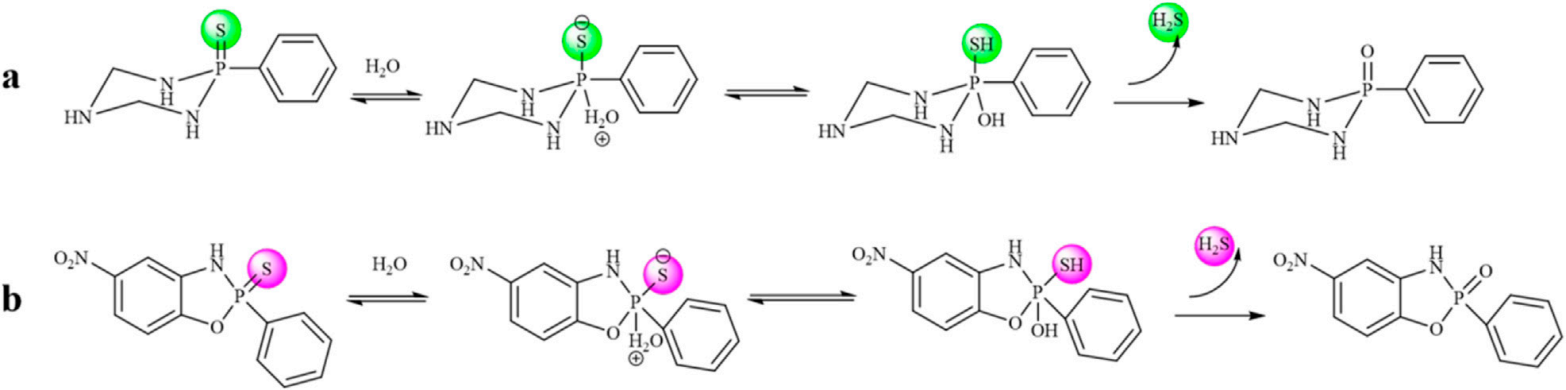
**(A)** Mechanism of compound **S2**-**S10** releasing H_2_S. **(B)** Mechanism of compound **S11**-**S16** releasing H_2_S.

The H_2_S-release capability of the donors was measured using the methylene blue (MB^+^) method (Sun et al., 2022). This method is based on the reaction of zinc acetate with H_2_S to form zinc sulfide, which then reacts with *N, N*-dimethyl-1,4-phenylenediamine sulfate to generate methylene blue in the presence of ferric ions under acid medium condition. The H_2_S is quantified by measuring the absorbance of the solution at 670 nm. Recently, literature has reported that TECP (a water-soluble phosphine reducing agent) or cysteine can promote the release of H_2_S. Therefore, in the presence of TECP or cysteine, the MB⁺ water titration method was used to evaluate the H₂S release ability (Nelmi et al., 2013; Hasegawa and Viles, 2014). The level of pH varies in various tissues and organs of the body; for example, the pH of the small intestine is about 8.0, whereas the pH of gastric juice is about 1.8. Therefore, we tested the release of H_2_S under different temperature and pH conditions. The results, as shown in Figure 2 and Supplementary Figure 1, showed that all compounds released H_2_S at room temperature. The release of H_2_S increased when the temperature was 37°C, indicating that temperature affects the amount of gas released. Different pH values have relatively little effect on such H_2_S donors. The release amount of compound **S11** is the highest, indicating that the size of the cyclic structure and functional groups such as esters and amides have little effect on the release of H_2_S. However, the addition of a benzene ring can increase its release amount, which may be due to the electron transfer of the benzene ring increasing the release amount of H_2_S. The mechanism of H_2_S release was proposed, as shown in Figure 3.

### 2.3 The toxicity of the compounds

Compounds **S1** - **S16** were assessed for toxicities against LO2 cell lines (normal liver cell) and HepG2 cell lines (liver tumor cell) using MTT assay. Stock solutions of the tested compounds in DMSO (8,000 mM) were prepared freshly immediately prior to testing. Initially, cells were seeded in a 96-well plate at approximately 10^5^ cells/well and allowed to adhere overnight. Cell survival relative to control was assessed after 24 h (Sanchez et al., 2019). The results are shown in Table 1. In summary, the toxicities of the series compounds are low to both normal cells LO2, WI38 and tumor cells HepG2, indicating that these compounds do not cause severe harm to the liver severely in the range of tested dose.

**TABLE 1:** IC_50_ (μM) values of all the compounds.

Compound	HepG2^a^ ^,^ ^b^	LO2^a^ ^,^ ^b^	WI38^a^ ^,^ ^b^
S1	>400	>200	>200
S2	>400	>200	>200
S3	>400	>200	>200
S4	>400	>200	>200
S5	>400	>200	>200
S6	>400	>200	>200
S7	>400	>200	>200
S8	>400	>200	>200
S9	>400	>200	>200
S10	>400	>200	>200
S11	>400	>200	>200
S12	>400	>200	>200
S13	>400	>200	>200
S14	>400	>200	>200
S15	>400	>200	>200
S16	>400	>200	>200
5-FU	105	170	202

^a^ Each experiment was repeated three times.

^b^ *IC50* is the minimum concentration of a drug that is toxic to 50% of the cells.

### 2.4 The antitumor activity of the compounds

The anti-proliferative activity of H_2_S on tumor cells has been proven (Zhao et al., 2010; Faris et al., 2023; Liu et al., 2023). This experiment demonstrated that the P = S functional group is an effective functional group for releasing H_2_S and has good anti-tumour activity. The results, as shown in Table 2 and Figure 2, showed that **S11** had high H_2_S release and good antiproliferative activity against MCF7. Based on the highest release of H_2_S with lower toxicity, we chose compound **S11** for subsequent studies. We found that the amount of hydrogen sulfide released was positively correlated with the anticancer activity, and the highest amount of hydrogen sulfide was released from **S11**, **S14** and **S16**, which showed the best activity against MCF7 cells. The activity of aminophenol derivatives was superior to that of 2,2′-dihydroxybiphenyl derivatives. The coupling of aminophenol derivatives to **S1** with electron-withdrawing groups contributed to the activity, and nitro was preferred to cyano to methyl.

**TABLE 2:** IC_50_ (μM) values of all compounds against cell proliferation.

Compound	HeLa^a^ ^,^ ^b^	A549^a^ ^,^ ^b^	MCF-7^a^ ^,^ ^b^
S1	726 ± 12.3	673 ± 22.4	230 ± 9.5
S2	736 ± 22.1	650 ± 32.5	455 ± 3.2
S3	826 ± 17.1	700 ± 32.3	470 ± 4.1
S4	836 ± 19.7	720 ± 22.4	472 ± 3.3
S5	622 ± 20.6	680 ± 20.5	468 ± 3.4
S6	645 ± 15.5	710 ± 22.6	471 ± 5.4
S7	655 ± 15.3	730 ± 32.3	473 ± 4.2
S8	730 ± 20.5	803 ± 20.8	480 ± 3.8
S9	730 ± 20.7	590 ± 27.1	459 ± 3.7
S10	603 ± 20.8	557 ± 18.5	455 ± 2.4
S11	260 ± 7.6	294 ± 16.6	23 ± 3.2
S12	703 ± 17.7	640 ± 15.5	164 ± 4.1
S13	633 ± 18.3	500 ± 18.2	460 ± 3.5
S14	530 ± 11.3	220 ± 20.1	31 ± 2.5
S15	733 ± 28.2	520 ± 22.7	266 ± 3.5
S16	520 ± 20.3	280 ± 28.3	28 ± 2.7
5-FU	120 ± 4.1	104 ± 5.2	136 ± 5.4

^a^ Each experiment was repeated three times.

^b^ *IC50* is the minimum concentration of a drug that is toxic to 50% of the cells.

### 2.5 Assessment of anti-tumour mechanisms

#### 2.5.1 Venn diagram construction and core PPI network screening

**FIGURE 4:**
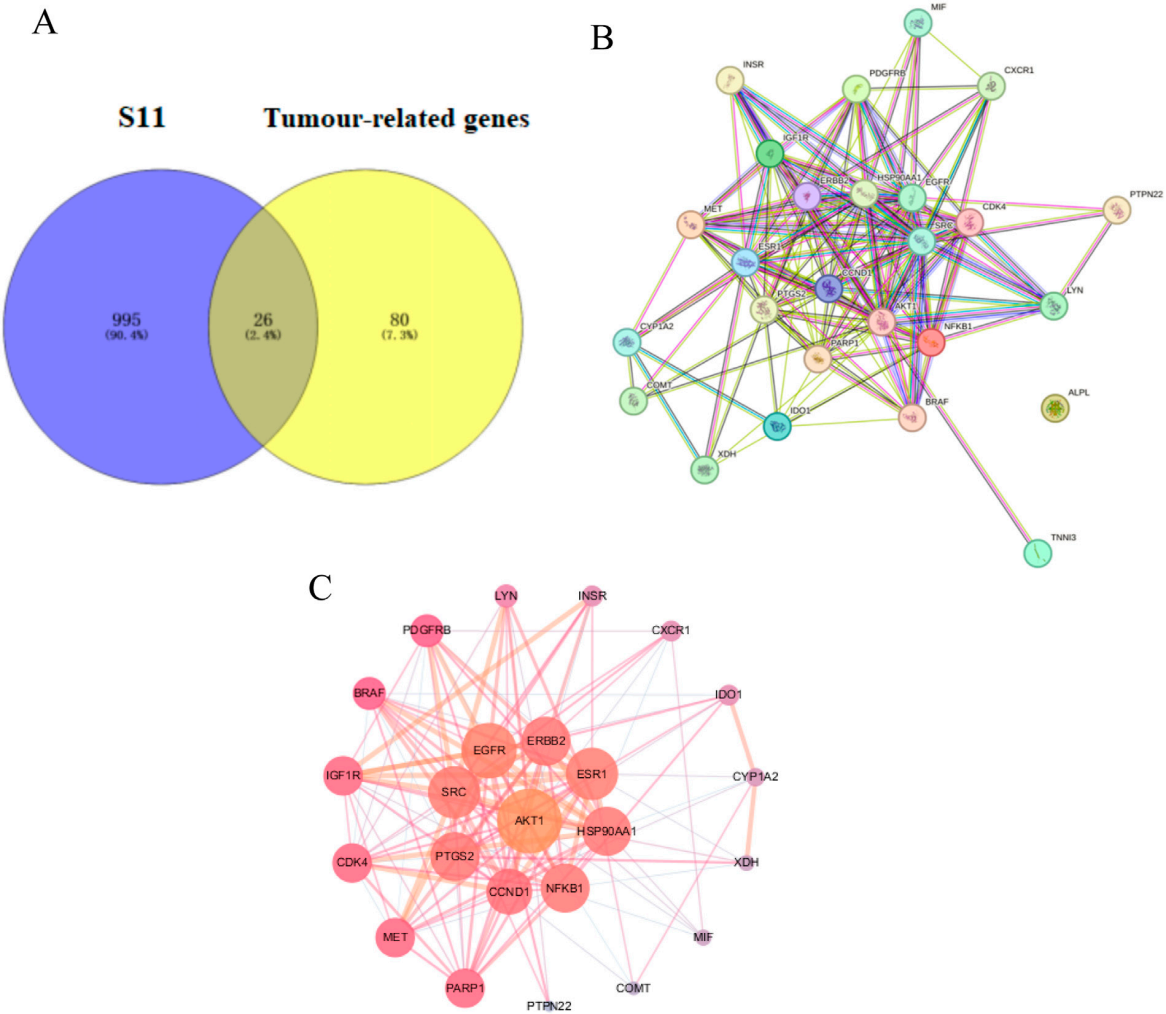
**(A)** Venn diagram reveals the relationship between **S11** and Tumour-related genes. **(B)** Construction of a PPI network of the interactive PPI network of **S11** and tumour targets by the STRING database. **(C)** The interactive PPI network of **S11** and tumour targets by Cytoscape software.

The Venn diagram in Figure 4A visualizes the overlap of **S11** with tumour targets, revealing 26 common genes. It is suggested that **S11** may regulate tumour cell death by interacting with these targets. We then constructed 2 networks using STRING for **S11** (Figure 4B) and an intersecting network of **S11** with cancer target (Figure 4C). Circles in Figure 4C indicate targets of drug-disease interactions; larger circles indicate more significant interactions between targets, suggesting that their respective functions are more important. Combined with Figure 4C, AKT is an important potential target for our analysed.

#### 2.5.2 GO and KEGG pathway enrichment analysis

**FIGURE 5:**
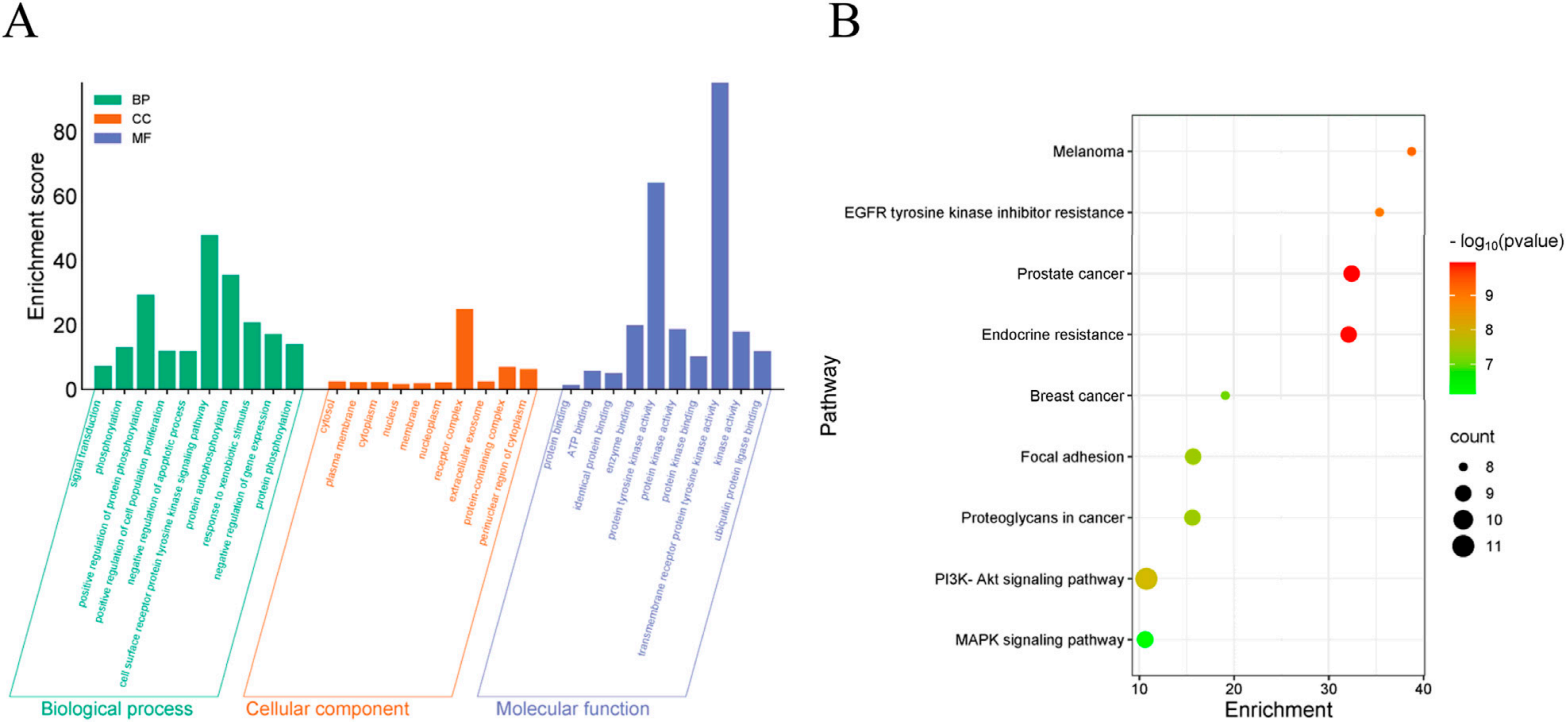
GO analysis and KEGG enrichment analysis of candidate target genes **(A)** Visualized enrichment bubble plots of biological processes (BP), cell components (CC) molecular function (MF). **(B)** Visualized enrichment bubble plots of Kyoto Encyclopedia of Genes and Genomes (KEGG). The term “RichFactor” refers to the proportion of differentially expressed transcripts found in a specific Gene Ontology (GO) entry compared to the total number of transcripts within that GO entry among all annotated transcripts. A higher RichFactor indicates a higher level of enrichment. The points on the graph are colored differently to indicate distinct P values, and the size of the points represents the number of target genes within the pathway.

The results of GO (BP, CC, MF) indicated that the top 10 targets are primarily membrane-associated and involved in lipid metabolism and biosynthesis processes (Figure 5A). KEGG enrichment bubble diagrams analysis identified two significant pathways relevant to **S11**’s therapeutic action, notably the PI3K-AKT signaling pathway and MAPK signaling pathway related to tumor disease (Figure 5B). These analyses underscore the important role of **S11** in the treatment of tumor disease.

#### 2.5.3 Molecular docking

**FIGURE 6:**
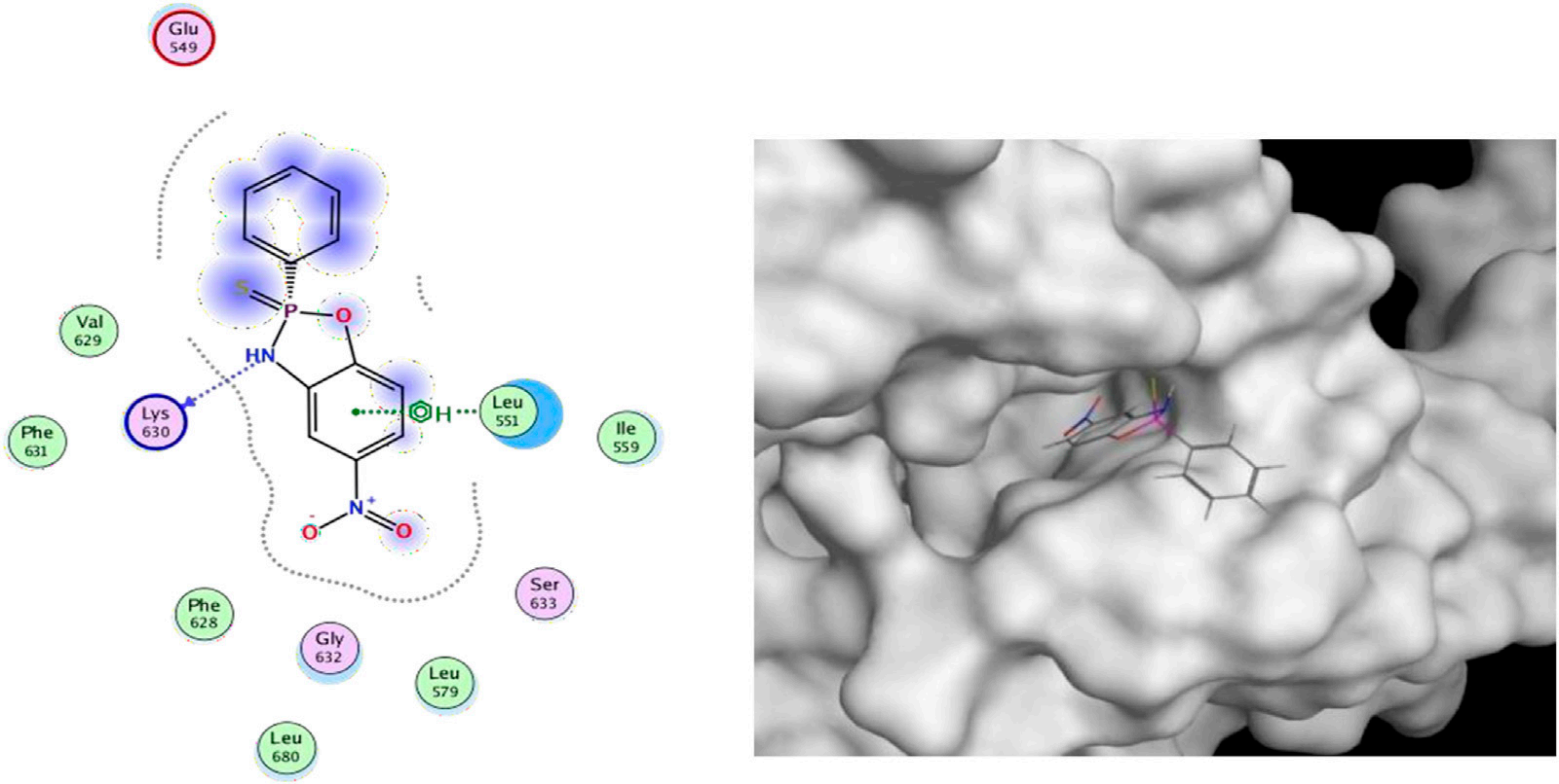
(Left) 2D ligands interaction diagram of the most active compounds (**S11**) with surrounding residues of the most probable binding site (Right) 3D diagram of compound **S11** binding to this site.

For this binding site (7F7W), both GlideScore and Model scores have a significant positive correlation with biological activity. This binding site has good tuberculosis with **S11** and can form hydrogen bonding interactions (Figure 6). And the twist angle of **S11** has changed, which may be caused by newly formed hydrophobic interactions and van der Waals forces between **S11** and the ligand (Khan et al., 2022; Hu et al., 2022; Zhu et al., 2021). This indicates that our compound **S11** may induce cell apoptosis by interacting with this protein, thereby inhibiting the PI3K/AKT signaling pathway mechanism.

#### 2.5.4 Evaluation of anti-tumour targets

PI3K pathway aberrations are the most common in cancer and one of the most widely studied pathways in cancer therapy. To date, more than 30 PI3K inhibitors have entered clinical trials for various cancer types, and aberrant activation of PI3K and its downstream effectors, including Akt and mTOR, has been associated with a variety of cancers. AKT is a central node of the PI3K/AKT/mTOR signalling pathway, which is involved in the regulation of cell proliferation, differentiation and apoptosis and promotes tumourigenesis and metastasis, AKT is over-activated in more than 50% of tumours, including breast, lung, head and neck, endometrial, prostate and colorectal cancers (Hart et al., 2011). In conjunction with our previously predicted anticancer targets and potential mechanistic pathways, in order to further validate the antitumour mechanism of **S11**, we used a PI3K/AKT enzyme inhibition assay. The results, as shown in Table 3, showed that compound **S11** had a better inhibitory effect on PI3Kα, PI3Kβ, PI3Kγ, and AKT using PI-103 as a control, validating our predicted mechanistic pathway. We tentatively suggest that **S11** induces tumour cell death by regulating the PI3K/AKT/mTOR signalling pathway, which in turn induces tumour cell death.

**TABLE 3:** The inhibitory activity of synthesised compounds on PI3K isoforms.

Compound	PI3Kα%inhibition at 10 μM	PI3Kβ% inhibition at 10 μM	PI3Kγ% inhibition at 10 μM	AKT% inhibition at 10 μM
**S11**	67	75	45	61
PI-103	100	5	12	100

### 2.6 The anti-inflammatory activity of the compounds

#### 2.6.1 Effect of compounds on cell activity

**FIGURE 7:**
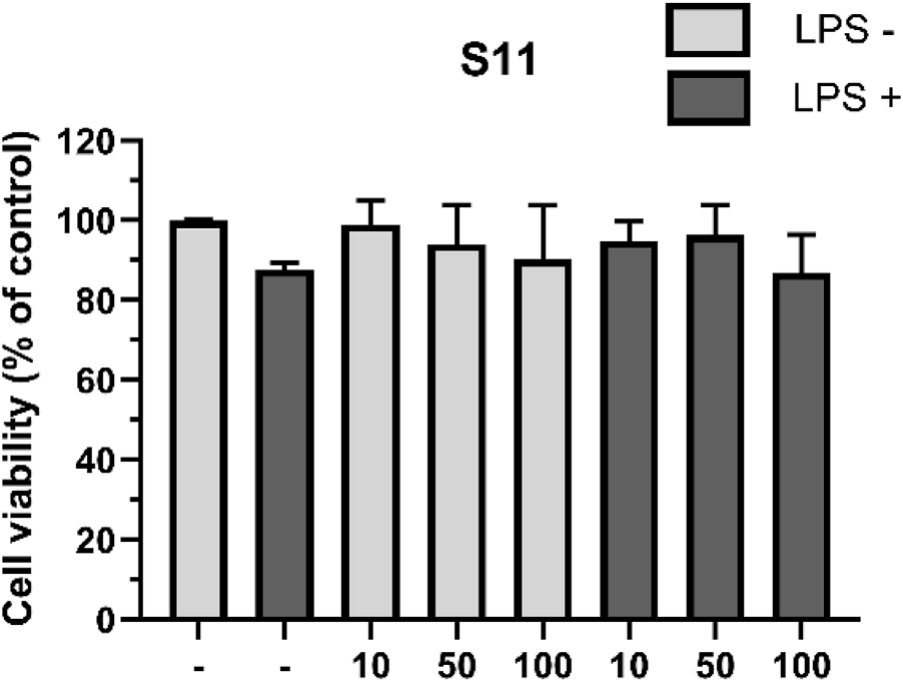
The effect of compounds on RAW264.7 cell viability. Mouse RAW264.7 macrophage cells were treated with compound **S11** at concentrations of 10, 50, and 100 µM for 24 h in the presence or absence of 1 µg/L LPS. Each bar represents the average standard deviation of three independent experiments.

To obtain more accurate anti-inflammatory results and minimize the impact of cell death caused by the test compounds on the outcomes, we first evaluated the effects of the compounds on RAW264.7 macrophage viability. The cells were divided into two groups: one treated with 1 mg/mL LPS and the other untreated. Macrophages were exposed to compounds at concentrations of 10, 50, and 100 μM for 24 h, followed by cell viability measurement using the CCK-8 assay (Huang et al., 2022; Flannigan and Wallace, 2015; Citi et al., 2020; Gemici and Wallace, 2015). The results showed that compound **S11** had low toxicity to macrophages and had no significant effect on cell growth in the absence of LPS (Figure 7). In the presence of LPS stimulation, a slight reduction of 11% cell activity was observed.

#### 2.6.2 Impact of the compounds on nitrite production induced by LPS

Nitric oxide (NO) affects inflammation signaling pathways, regulating the intensity and timing of inflammatory responses, making it a crucial regulatory molecule in inflammation. LPS induces the production of nitric oxide (NO) by activating a series of signal transduction pathways. Upon NF-κB activation, transcription of the iNOS gene is promoted, leading to the synthesis of inducible nitric oxide synthase (iNOS). iNOS is an enzyme that produces large amounts of NO, activated under inflammatory conditions to release substantial nitric oxide (Kumar, 2023). *In vivo*, NO is easily oxidized by superoxide ions to generate nitrites. Studies have shown that H_2_S not only acts to scavenge peroxides but also undergoes chemical reactions with NO, forming novel nitrosylthiol compounds. We assess the anti-inflammatory activity of compounds by measuring nitrite production (Somensi et al., 2019).

**FIGURE 8:**
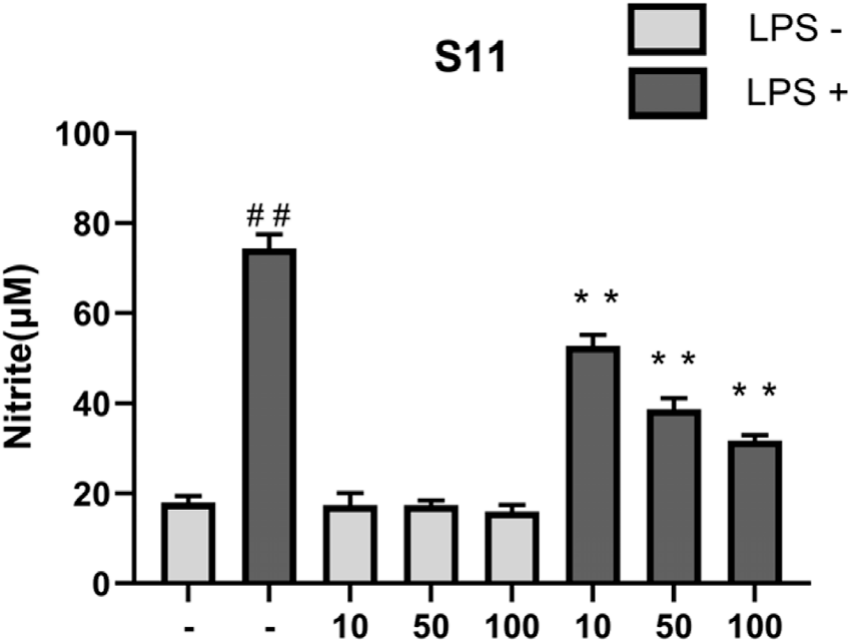
The effect of compounds on nitrite levels in RAW 264.7 cells. Mouse RAW264.7 macrophage cells were treated with compound **S11** at concentrations of 10, 50, and 100 µM for 24 h in the presence or absence of 1 µg/mL LPS. Each bar represents the mean ± SD of three independent experiments. ^#^p < 0.05, ^##^p < 0.01 vs untreated cells; *p < 0.05, **p < 0.01, vs LPS treated cells.

Results indicate (Figure 8) that under conditions of 1 mg/mL LPS, compound **S11** can reduce nitrite levels and showing significant inhibition of iNOS. **S11** show clear dose-dependency. At 100 μM, compound **S11** inhibits nitrite production by approximately 75%. Considering the H_2_S release results, the high level of H_2_S release in **S11** compounds may be the reason for their significant anti-inflammatory activity.

#### 2.6.3 Effect of the compounds on LPS-mediated TNF-α and IL-10

Anti-inflammatory drugs typically exert their effects by inhibiting the NF-κB signaling pathway. Upon NF-κB activation, not only does it promote the transcription of the iNOS gene, but it also increases the expression of various inflammation-related genes such as TNF-α, IL-1β, etc. (Hou et al., 2006). Among these, TNF-α is a key pro-inflammatory cytokine in inflammation responses (You et al., 2022). It can activate immune cells, enhance inflammation signal transduction, and trigger inflammatory reactions, including leukocyte migration and cytokine release. Studies have shown that H_2_S can significantly reduce the number of neutrophils induced by LPS in the liver and lungs, and decrease TNF-α levels (Chiba et al., 2007; Pan et al., 2023; Huang et al., 2022).

**FIGURE 9:**
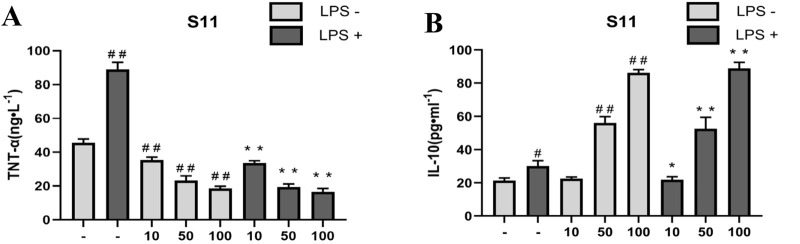
**(A)** The effect of compounds on TNT-α levels in RAW 264.7 cells. **(B)** The effect of compound on IL-10 level in RAW264.7 cells. Mouse RAW264.7 macrophage cells were treated with compound **S11** at concentrations of 10, 50, and 100 µM for 24 h in the presence or absence of 1 µg/mL LPS. Each bar represents the mean ± SD of three independent experiments. ^#^p < 0.05, ^##^p < 0.01 vs untreated cells; *p < 0.05, **p < 0.01, vs LPS treated cells.

Therefore, we assessed the anti-inflammatory ability of compounds by measuring their effect on TNF-α levels in RAW264.7 cells induced by LPS. Results showed (Figure 9A) that compared to the control group, **S11** inhibited TNF-α expression (Figure 8). Compound **S11** decreased TNF-α induction levels by 80%. H_2_S also promotes an increase in plasma IL-10 levels, thereby inhibiting inflammatory mediators. Comparatively (Figure 9B), compound **S11** exhibited the highest induction level of IL-10, being three times that of the control group.

#### 2.6.4 Effect of the compounds on LPS-mediated HO-1

**FIGURE 10:**
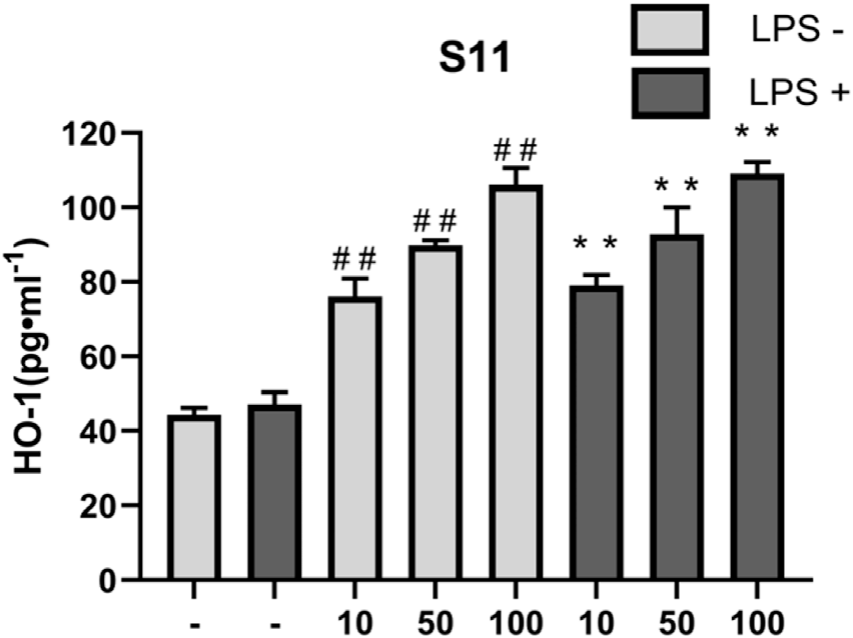
The effect of compounds on HO-1 levels in RAW 264.7 cells. Mouse RAW264.7 macrophage cells were treated with compound **S11** at concentrations of 10, 50, and 100 µM for 24 h in the presence or absence of 1 µg mL-1 LPS. Each bar represents the mean ± SD of three independent experiments. ^#^p < 0.05, ^##^p < 0.01 vs untreated cells; *p < 0.05, **p < 0.01, vs LPS treated cells.

HO-1 is an important antioxidant enzyme involved in regulating intracellular redox balance. It reduces the generation of oxygen free radicals by degrading products such as carbon monoxide (CO) and biliverdin derived from heme, helping to inhibit oxidative stress and cell damage, and reducing the severity of inflammatory responses. Therefore, the expression level of HO-1 reflects the cell’s anti-inflammatory ability to some extent (Shen et al., 2022; Hou et al., 2021; Lee et al., 2022). Nrf2 is a major regulator of the antioxidant defense system *in vivo*, and is involved in signal transduction related to various intracellular defense mechanisms. HO-1 is a target protein downstream of Nrf2, and HO-1 and its products play beneficial roles in modulating inflammatory responses. It has been shown that active compounds can attenuate lipopolysaccharide-induced cellular inflammatory responses by increasing the amount of HO-1 and thereby activating the Nrf2/HO-1 pathway (Lee et al., 2022; Ali et al., 2018). In this experiment, compound **S11** was used to treat RAW264.7 cells, and the expression levels of HO-1 were measured. The results showed that compound **S11** promoted HO-1 expression to approximately twice the level of the control group (Figure 10).

## 3 Conclusion

This experiment aimed to investigate the multifaceted effects of H_2_S in anti-cancer, anti-inflammatory, and other aspects. To develop superior H_2_S donor drugs, we designed a series of H_2_S donor compounds based on thiol-phosphorus core and evaluated these compounds extensively. Specifically, evaluations included assessing the H_2_S releasing capability of the compounds, their toxicity and activity impact on cells, anti-tumour and anti-inflammatory effects. Through these assessments, we aimed to identify H_2_S donor drugs with excellent anti-tumour and anti-inflammatory activities, providing valuable references and guidance for drug development in related fields.

This experiment found that all compounds could release H_2_S, with compound **S11** showing the most significant effect, possibly due to its benzene ring content. After treating LO2, HepG2, and WI38 cell lines with the compound **S1**-**S13** showed low cytotoxicity. Compound **S11** exhibited promising results in anticancer and anti-inflammatory assays. We performed target screening prediction by network pharmacology and validated it by molecular docking and enzyme inhibition assays, and found that compound **S11** may cause cancer cell death by inhibiting the PI3K/AKT pathway. *In vitro*, **S11** was found to have a better anti-inflammatory effect by the assay of some series of anti-inflammatory active factors TNF-α, IL-10 and HO-1.

## 4 Experimental section

All the key intermediates and final products were identified with ^1^H NMR and ^13^C NMR, recorded in a Bruker Avance 400 (^1^H at 400 MHz, ^13^C at 101 MHz), and chemical shifts were reported in parts per million using the residual solvent peaks as internal standards (CDCl_3_ = 7.26 ppm for ^1^H NMR and 77.16 ppm for ^13^C NMR).

### 4.1 Chemically synthetical experiments

#### 4.1.1 Synthesis of compound dichloro (phenyl)phosphane **(B)**

We added 0.1 mmol of benzene, 0.3 mmol of phosphorus trichloride and 0.14 mmol of aluminium chloride to the reaction flask and refluxed with stirring for 5 h. Then add 0.13 mol of phosphorus trichloride and 45 mL of petroleum ether and stir the mixture at reflux for 30 min. After cooling to room temperature, filter the mixture under reduced pressure, distill the filtrate with petroleum ether at atmospheric pressure, and distill under reduced pressure. Collect fractions from 99°C–101°C with a yield of 78%. ^1^H NMR (400 MHz, Chloroform-d) δ 7.94 (t, *J* = 8.6 Hz, 2H), 7.60–7.50 (m, 3H). ^13^C NMR (101 MHz, Chloroform-d) δ 140.46 (d, *J* = 52.2 Hz), 132.79, 130.17 (d, *J* = 31.3 Hz), 129.00 (d, *J* = 7.9 Hz). ^31^P NMR (162 MHz, Chloroform-d) 169.55. TOF-MS, m/z [M + H+], calcd. for C_6_H_6_Cl_2_P^+^, 178.9506, found: 178.9531.

#### 4.1.2 Synthesis of compound phenylphosphonothioic dichloride **(S1)**

Slowly add the settled sulfur powder in batches to the previous product **(B)**, control the vulcanization process temperature to 30°C, most of the sulfur powder disappears, and the reaction solution becomes viscous. Then, raise the temperature to 80°C and stir for 1 h. Atmospheric and vacuum distillation removes unreacted phosphorus trichloride and benzene. Collect the 90°C–91°C fraction, which is the product phenyl thiophosphine dichloride. Yield 48%. 1H NMR (400 MHz, Chloroform-d) δ 8.13 (dd, *J* = 18.6, 7.9 Hz, 2H), 7.63 (dt, *J* = 6.0, 3.0 Hz, 1H), 7.60–7.50 (m, 2H). ^13^C NMR (101 MHz, CDCl_3_) δ 138.07 (d, *J* = 118.2 Hz), 133.81 (d, *J* = 4.0 Hz), 129.99 (d, *J* = 15.2 Hz), 128.70 (d, *J* = 18.2 Hz). ^31^P NMR (162 MHz, Chloroform-d) 169.56. TOF-MS, m/z [M + H+], calcd. for C_6_H_6_Cl_2_SP^+^, 210.9227, found: 210.9237.

#### 4.1.3 Synthesis of compound 2-phenyl-1,3,2-oxazaphospholidine 2-sulfide **(S2)**

Di-aminoethanol (2 mmol) and triethylamine (1 mL) were dissolved in dichloromethane. Phenylphosphine dichloride (2 mmol) was added slowly in an ice bath. The reaction is then carried out at room temperature for 8 h. The precipitate was filtered and the solution was collected and concentrated. The crude product was purified by column chromatography (PE/EA = 4:1) to give a white solid. Yield 43%. Separation and purification methods can also be found in the literature (zhang et al., 2019). ^1^H NMR (400 MHz, Chloroform-d) δ 8.00–7.75 (m, 2H), 7.54–7.29 (m, 3H), 4.53–4.40 (m, 1H), 4.38–4.23 (m, 1H), 3.80–3.60 (m, 1H), 3.54–3.34 (m, 1H), 3.16–2.99 (m, 1H). ^13^C NMR (101 MHz, Chloroform-d) δ 135.80 (d, *J* = 135.0 Hz), 132.14 (d, *J* = 3.2 Hz), 130.89 (d, *J* = 12.3 Hz), 128.42 (d, *J* = 14.8 Hz), 68.35, 43.97.^31^P NMR (162 MHz, DMSO) 169.50. TOF-MS, m/z [M + H^+^], calcd. for C_8_H_11_NOPS^+^, 199.0221, found: 200.0247.

#### 4.1.4 Synthesis of compound 2-phenyl-1,3,2-oxazaphosphinane 2-sulfide **(S3)**

The method is the same as **S2**. Yield 47%. 1H NMR (400 MHz, Chloroform-d) δ 7.87 (ddd, *J* = 21.8, 13.8, 8.0 Hz, 2H), 7.48 (dt, *J* = 7.4, 4.8 Hz, 3H), 4.45 (td, *J* = 16.2, 7.1 Hz, 1H), 4.18–3.97 (m, 1H), 3.52–3.06 (m, 3H), 2.06 (ddt, *J* = 20.1, 10.1, 5.0 Hz, 1H), 1.62 (d, *J* = 14.3 Hz, 1H). ^13^C NMR (101 MHz, Chloroform-d) δ 133.67 (d, *J* = 133.7 Hz), 131.90 (d, *J* = 3.2 Hz), 130.77 (d, *J* = 11.3 Hz), 128.96 (d, *J* = 14.1 Hz), 67.84, 41.33, 26.70. ^31^P NMR (162 MHz, DMSO) 169.51. TOF-MS, m/z [M + H^+^], calcd. for C_9_H_13_NOPS^+^, 213.0377, found: 214.0355.

#### 4.1.5 Synthesis of compound 2-phenyl-1,3,2-oxazaphosphepane 2-sulfide **(S4)**

The method is the same as **S2**. Yield 50%. ^1^H NMR (400 MHz, Chloroform-d) δ 7.87 (dd, *J* = 13.8, 7.2 Hz, 2H), 7.56–7.34 (m, 3H), 4.68–4.45 (m, 1H), 4.17 (dd, *J* = 22.9, 12.0 Hz, 1H), 3.45 (s, 1H), 3.09 (dd, *J* = 30.1, 14.8 Hz, 1H), 2.74 (d, *J* = 13.9 Hz, 1H), 1.97–1.67 (m, 3H), 1.63–1.45 (m, 1H). ^13^C NMR (101 MHz, Chloroform-d) δ 135.13 (d, *J* = 148.6 Hz), 131.41 (d, *J* = 3.2 Hz), 130.32 (d, *J* = 11.0 Hz), 128.37 (d, *J* = 14.5 Hz), 64.95, 42.65, 31.69, 29.70. ^31^P NMR (162 MHz, DMSO) 169.50. TOF-MS, m/z [M + H^+^], calcd. for C_10_H_15_NOPS^+^, 228.0534, found: 228.0599.

#### 4.1.6 Synthesis of compound 2-phenyl-1,3,2-oxazaphosphocane 2-sulfide **(S5)**

The method is the same as **S2**. Yield 50%. ^1^H NMR (400 MHz, Chloroform-d) δ 7.75 (dd, *J* = 13.1, 8.2 Hz, 2H), 7.38–7.28 (m, 3H), 4.56 (qd, *J* = 9.7, 5.9 Hz, 1H), 4.07–3.85 (m, 1H), 3.49 (s, 1H), 3.02–2.84 (m, 1H), 2.78–2.62 (m, 1H), 1.98–1.82 (m, 1H), 1.73–1.33 (m, 5H). ^13^C NMR (101 MHz, Chloroform-d) δ 134.89 (d, *J* = 149.3 Hz), 130.81 (d, *J* = 3.1 Hz), 129.87 (d, *J* = 11.0 Hz), 127.93 (d, *J* = 14.5 Hz), 65.57, 53.39, 41.80, 29.57, 28.52, 23.62. ^31^P NMR (162 MHz, DMSO) 169.50. TOF-MS, m/z [M + H^+^], calcd. For C_11_H_17_NOPS^+^, 242.0690, found: 242.0677.

#### 4.1.7 Synthesis of compound 2-phenyl-1,3,2-oxazaphosphonane 2-sulfide **(S6)**

The method is the same as **S2**. Yield 50%. ^1^H NMR (400 MHz, Chloroform-d) δ 7.82 (dd, *J* = 13.7, 8.0 Hz, 2H), 7.52–7.35 (m, 3H), 4.87–4.71 (m, 1H), 4.06–3.89 (m, 1H), 3.27 (s, 1H), 2.97 (t, *J* = 15.1 Hz, 1H), 2.84 (s, 1H), 1.86 (d, *J* = 14.8 Hz, 1H), 1.77–1.61 (m, 2H), 1.59–1.31 (m, 5H). ^13^C NMR (101 MHz, Chloroform-d) δ 134.51 (d, *J* = 149.7 Hz), 131.32 (d, *J* = 3.1 Hz), 130.66 (d, *J* = 10.8 Hz), 128.43 (d, *J* = 14.4 Hz), 62.82, 40.51, 28.53, 27.52, 20.60, 20.04.^31^P NMR (162 MHz, DMSO) 169.51. TOF-MS, m/z [M + H^+^], calcd. for C_12_H_19_NOPS^+^, 256.0847, found: 256.0866.

#### 4.1.8 Synthesis of compound 2-phenyl-1,3,2-diazaphosphepane 2-sulfide **(S7)**

The method is the same as **S2**. Yield 50%. ^1^H NMR (400 MHz, Chloroform-d) δ 7.91 (dd, *J* = 13.6, 7.1 Hz, 2H), 7.47–7.31 (m, 3H), 3.34–3.11 (m, 2H), 3.09–2.84 (m, 4H), 1.73–1.52 (m, 4H). ^13^C NMR (101 MHz, Chloroform-d) δ 136.39 (d, *J* = 126.8 Hz), 131.29 (d, *J* = 3.0 Hz), 130.32 (d, *J* = 11.0 Hz), 128.28 (d, *J* = 13.6 Hz), 41.56, 41.53, 31.72. ^31^P NMR (162 MHz, DMSO) 169.51. TOF-MS, m/z [M + H^+^], calcd. for C_10_H_16_N_2_PS^+^, 227.0694, found: 227.0780.

#### 4.1.9 Synthesis of compound 2-phenyl-1,3,2-dioxaphosphepane 2-sulfide **(S8)**

The method is the same as **S2**. Yield 50%. ^1^H NMR (400 MHz, Chloroform-d) δ 7.79 (dd, *J* = 13.5, 7.6 Hz, 2H), 7.46–7.29 (m, 3H), 4.24 (dd, *J* = 23.8, 5.8 Hz, 2H), 3.95 (td, *J* = 11.7, 5.9 Hz, 2H), 1.96–1.68 (m, 4H). ^13^C NMR (101 MHz, Chloroform-d) δ 132.34 (d, *J* = 149.7 Hz), 131.82 (d, *J* = 3.1 Hz), 130.10 (d, *J* = 11.3 Hz), 128.06 (d, *J* = 14.8 Hz), 67.07, 28.96. ^31^P NMR (162 MHz, DMSO) 169.51. TOF-MS, m/z [M + H^+^], calcd. for C_10_H_14_O_2_PS^+^, 229.0374, found: 229.0398.

#### 4.1.10 Synthesis of compound 2-phenyl-1,3,5,2-triazaphosphinane 2-sulfide **(S9)**

The method is the same as **S2**. Yield 50%. 1H NMR (400 MHz, Chloroform-d) δ 7.90–7.65 (m, 2H), 7.42–7.27 (m, 3H), 3.50–2.69 (m, 7H). ^13^C NMR (101 MHz, Chloroform-d) δ 135.83 (d, *J* = 134.33 Hz), 132.78 (d, *J* = 13.1 Hz), 130.81 (d, *J* = 11.1 Hz), 128.11 (d, *J* = 14.1 Hz), 48.17, 42.12, 38.80. ^31^P NMR (162 MHz, DMSO) 169.51. TOF-MS, m/z [M + H^+^], calcd. for C_8_H_13_N_3_PS^+^, 214.0490, found: 214.0532.

#### 4.1.11 Synthesis of compound 2-phenyl-1,3,2-dithiaphospholane 2-sulfide (S10)

The method is the same as **S2**. Yield 50%. ^1^H NMR (400 MHz, Chloroform-d) δ 8.12 (dd, *J* = 16.9, 7.4 Hz, 2H), 7.54–7.46 (m, 3H), 3.89–3.75 (m, 2H), 3.75–3.58 (m, 2H). ^13^C NMR (101 MHz, Chloroform-d) δ 137.18 (d, *J* = 83.5 Hz), 132.28 (d, *J* = 3.5 Hz), 131.34 (d, *J* = 12.8 Hz), 128.52 (d, *J* = 14.6 Hz), 43.00. ^31^P NMR (162 MHz, DMSO) 169.51. TOF-MS, m/z [M + H^+^], calcd. For C_8_H_10_PS^+^, 232.9604, found: 232.9677.

#### 4.1.12 Synthesis of compound 5-nitro-2-phenyl-3H-benzo [*d*][1,3,2] oxazaphosphole 2-sulfide **(S11)**

The method is the same as **S2**. Yield 50%. ^1^H NMR (400 MHz, DMSO) δ 8.28–8.16 (m, 1H), 7.89 (d, *J* = 7.1 Hz, 2H), 7.82–7.73 (m, 1H), 7.72–7.61 (m, 1H), 6.85 (d, *J* = 9.7 Hz, 1H), 6.69 (s, 3H). ^13^C NMR (101 MHz, DMSO-d6) δ 147.72, 134.81, 134.35 (d, *J* = 7.7 Hz), 134.11 (d, *J* = 3.1 Hz), 131.66 (d, *J* = 12.9 Hz), 131.28, 129.80, 128.99 (d, *J* = 15.6 Hz), 123.13, 117.16 (d, *J* = 4.0 Hz), 113.98. ^31^P NMR (162 MHz, DMSO) 169.51. TOF-MS, m/z [M + H^+^], calcd. For C_12_H_10_N_2_O_3_PS^+^, 293.0150, found: 293.0148.

#### 4.1.13 Synthesis of compound 6-phenyldibenzo [*d, f*][1,3,2] dioxaphosphepine 6-sulfide **(S12)**

The method is the same as **S2**. Yield 50%. ^1^H NMR (400 MHz, Chloroform-d) δ 7.87 (dd, *J* = 14.3, 7.6 Hz, 2H), 7.59–7.47 (m, 3H), 7.42–7.24 (m, 6H), 7.10–7.01 (m, 2H). ^13^C NMR (101 MHz, Chloroform-d) δ 148.01 (d, *J* = 11.7 Hz), 133.05 (d, *J* = 3.1 Hz), 131.33 (d, *J* = 11.5 Hz), 129.70 (d, *J* = 35.0 Hz), 129.04 (d, *J* = 1.8 Hz), 128.04 (d, *J* = 15.0 Hz), 126.27 (d, *J* = 1.8 Hz), 121.98 (d, *J* = 3.7 Hz). ^31^P NMR (162 MHz, DMSO) 169.50. TOF-MS, m/z [M + H^+^], calcd. For C_18_H_13_PS^+^, 325.0374, found: 325.0332.

#### 4.1.14 Synthesis of compound 3-phenyl-1,5-dihydrobenzo [*e*] [1,3,2] dioxaphosphepine 3-sulfide **(S13)**

The method is the same as **S2**. Yield 50%. ^1^H NMR (400 MHz, Chloroform-d) δ 8.04–7.86 (m, 2H), 7.55 (t, *J* = 7.3 Hz, 1H), 7.50–7.41 (m, 2H), 7.40–7.37 (m, 4H), 6.00 (t, *J* = 13.1 Hz, 2H), 4.94–4.80 (m, 2H). ^13^C NMR (101 MHz, Chloroform-d) δ 136.68, 133.02 (d, *J* = 3.1 Hz), 131.35 (d, *J* = 11.8 Hz), 129.19 (d, *J* = 53.5 Hz), 128.45 (d, *J* = 15.3 Hz). 67.47. ^31^P NMR (162 MHz, DMSO) 169.51. TOF-MS, m/z [M + H^+^], calcd. for C_14_H_14_O_2_PS^+^, 277.0374, found: 277.0331.

#### 4.1.15 Synthesis of compound 5-methyl-2-phenyl-3H benzo [*d*][1,3,2] oxazaphosphole 2-sulfide **(S14)**

The method is the same as **S2**. Yield 30%. ^1^H NMR (400 MHz, Chloroform-d) δ 7.82 (dd, *J* = 14.7, 7.8 Hz, 2H), 7.51–7.44 (m, 1H), 7.42–7.34 (m, 2H), 6.66 (s, 2H), 6.46 (d, *J* = 7.9 Hz, 1H), 5.82 (d, *J* = 10.2 Hz, 1H), 2.17 (s, 3H). ^13^C NMR (101 MHz, Chloroform-d) δ 146.24 (d, *J* = 7.4 Hz), 134.05–133.51 (m), 132.15 (d, *J* = 3.2 Hz), 130.93 (d, *J* = 11.4 Hz), 128.71 (d, *J* = 14.8 Hz), 124.83, 121.53, 120.20 (d, J = 2.9 Hz), 116.40, 20.89. ^31^P NMR (162 MHz, DMSO) 169.52. TOF-MS, m/z [M + H^+^], calcd. for C_13_H_13_NOPS^+^, 262.0455, found: 262.0459.

#### 4.1.16 Synthesis of 2-phenylperyleno [1,12-def][1,3,2] dioxaphosphepine 2-sulfide **(S15)**

The method is the same as **S2**. Yield 37%. ^1^H NMR (400 MHz, Chloroform-d) δ 8.09 (d, *J* = 8.8 Hz, 1H), 8.00 (d, *J* = 8.2 Hz, 1H), 7.92 (d, *J* = 8.1 Hz, 1H), 7.73 (ddd, *J* = 35.3, 19.9, 8.8 Hz, 3H), 7.59–7.46 (m, 4H), 7.42–7.29 (m, 4H), 6.91 (d, *J* = 8.8 Hz, 1H). ^13^C NMR (101 MHz, Chloroform-d) δ 151.05–144.86 (m), 133.39 (d, *J* = 3.1 Hz), 132.77 (d, *J* = 1.8 Hz), 132.19 (d, *J* = 1.5 Hz), 132.01 (d, *J* = 11.6 Hz), 131.73 (d, *J* = 1.3 Hz), 130.83 (dd, *J* = 42.0, 1.4 Hz), 128.51 (dd, *J* = 31.7, 14.0 Hz), 127.32 (d, *J* = 23.3 Hz), 126.76 (d, *J* = 6.5 Hz), 125.86, 122.13 (d, *J* = 2.4 Hz), 121.21 (d, *J* = 2.8 Hz). ^31^P NMR (162 MHz, DMSO) 169.52. TOF-MS, m/z [M + H^+^], calcd. for C_26_H_16_O_2_PS^+^, 423.0680, found: 423.0688.

#### 4.1.17 Synthesis of compound 2-phenyl-3H-benzo [*d*][1,3,2] oxazaphosphole-5-carbonitrile 2-sulfide **(S16)**

The method is the same as **S2**. Yield 48%. ^1^H NMR (400 MHz, Chloroform-d) δ 7.83 (dd, *J* = 14.7, 7.8 Hz, 2H), 7.51–7.45 (m, 1H), 7.44–7.35 (m, 2H), 6.80 (dd, *J* = 8.3, 5.6 Hz, 3H), 6.65 (t, *J* = 7.3 Hz, 1H). ^13^C NMR (101 MHz, Chloroform-d) δ 145.65 (d, *J* = 7.8 Hz), 133.85, 132.18 (d, *J* = 3.2 Hz), 130.88 (d, *J* = 11.5 Hz), 128.76 (d, *J* = 14.8 Hz), 127.91 (d, *J* = 3.1 Hz), 123.23, 121.14, 119.40 (d, *J* = 3.2 Hz), 115.58. ^31^P NMR (162 MHz, DMSO) 169.50. TOF-MS, m/z [M + H^+^], calcd. for C_26_H_16_O_2_PS^+^, 423.0680, found: 423.0688.

### 4.2 Cell culture

RAW264.7 cells Cultivated in this laboratory, were cultured in Dulbecco’s Modified Eagle’s Medium (DMEM) (Gibco) supplemented with 10% v/v heat inactivated fetal bovine serum (FBS) (Gibco), l-glutamine (4 mM), sodium pyruvate (1 mM), HEPES buffer (20 mM) (Hyclone), penicillin (100 U/mL) and streptomycin (100 μg/mL). Cells were seeded in non-tissue culture treated Petri dishes (Biomedia) and maintained at 37°C in a 5% CO_2_ humidified environment (Sanchez et al., 2019).

### 4.3 H_2_S measurement

The H_2_S-release capability of the donors was measured using the methylene blue (MB+) method. See Supplementary Material for more details (Sun et al., 2022; Hasegawa and Viles, 2014).

### 4.4 Venny map construction and core PPI network screening

We obtained SMILES of the active compound **S11** from PubChem database, and then obtained **S11** related information from TCSMP database, predicted and obtained related targets through PharmMapper database. Meanwhile, relevant targets were retrieved from Gene Cards database. Then five more databases were collected for cancer disease targets GenesCards database, CTD database, DisGeNET database, TTD database, OMIM database. Venn diagrams of **S11** *versus* cancer targets were obtained and visualised using the online tool Venny 2.0 (https://bioinfogp.cnb.csic.es/tools/venn y/index. html). Subsequently, the identified cross-targets were uploaded to the STRING (https://cn.string-dbOrg/) database, which predicts protein interactions. The free nodes were hidden to obtain the PPI network of **S11** with cancer. This part of the data was further analysed using Cytoscape 3.7.1 software (Zhang et al., 2019; Nogales et al., 2022).

### 4.5 GO and KEGG pathway enrichment analysis

To elucidate the biological significance of the gene list, we used Database Annotation, Visualisation and Integrated Discovery (DAVID), including GO and KEGG pathway enrichment analysis. We entered the cross-targets of **S11** and cancer in the PPI network into DAVID, specifying the species as “*Bos taurus*”. We then performed functional analyses of graphene oxide, including biological processes (BP), cellular components (CC) and molecular functions (MF), as well as KEGG pathway enrichment analyses of the core targets, and concluded that a p-value of <0.05 was statistically significant. Finally, the OmicShare platform (https://www.omicshare.com/) was used to process the data (Zhang et al., 2019; Nogales et al., 2022).

### 4.6 Cell viability

The CCK-8 assay is a commonly used method for assessing cell viability, based on cellular metabolic activity. See Supplementary Material for more details (Huang et al., 2022; Flannigan and Wallace, 2015; Citi et al., 2020; Gemici and Wallace, 2015).

### 4.7 Nitrite level detection

The Griess assay is used to measure nitrite levels in biological samples, both *in vivo* and *in vitro*. See Supplementary Material for more details (Kumar, 2023).

### 4.8 Detection of cytokine levels

In this experiment, the levels of TNF-α, IL-10, and HO-1 cell factors were assessed using the ELISA method (Shen et al., 2022; Hou et al., 2021; Lee et al., 2022). See Supplementary Material for more details.

### 4.9 *In vitro* PI3K and ATK enzyme inhibitory assay

Enzyme activity assays were performed according to the reagent kit and references, and detailed procedures are in the Supplementary Material (Huang et al., 2022).

### 4.10 Docking and collecting data

Selection of previously described 7F7W targets for molecular docking with **S11**. See Supplementary Material for more details (Khan et al., 2022; Hu et al., 2022; Zhu et al., 2021).

### 4.11 Statistical analysis

The above experimental data are the mean ± SD of at least three independent experiments. SPSS 22.0 software was used to process the data, and one-way analysis of variance (ANOVA) was used to measure statistical differences between the two groups.

## Data Availability

The datasets presented in this study can be found in online repositories. The names of the repository/repositories and accession number(s) can be found in the article/Supplementary Material.
