# Supplementary material for: Design and synthesis of phenylthiophosphoryl dichloride derivatives and evaluation of their antitumour and anti-inflammatory activities
Source: Front Chem. 2025 Jan 21;12:1529211. doi: 10.3389/fchem.2024.1529211 (PMC11790660; doi:10.3389/fchem.2024.1529211)
Supplement: Supplementary file 1 [file Supplementaryfile1.doc]

# Design and synthesis of phenylthiophosphoryl dichloride derivatives and evaluation of their antitumour and anti-inflammatory activities

Chunyun Xu[^1^](#_bookmark0)*, Na Yang^1^ , Haichun Yu^1^ , Xiaojing Wang^1^

*^1^Department of Dermatology, Maternity and Child Health Hospital of Qinhuangdao, Qinhuangdao, China, 066000, Qinhuangdao, China*

**Table of Contents**

**H2S release of the compounds**································································1

**H2S measurement**··············································································2

**Cell viability**·····················································································3

**Nitrite level detection**··········································································4

**Detection of cytokine levels**····································································5

**In vitro PI3K and ATK enzyme inhibitory assay**········································6

**Docking and Collecting Data**································································7

**Spectral data**················································································8-37

**H2S release of the compounds**


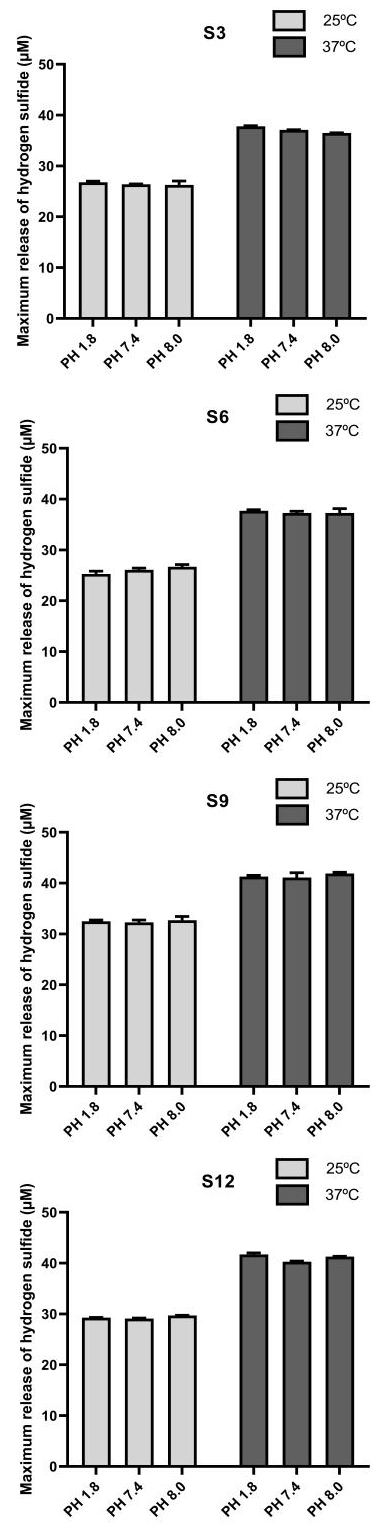

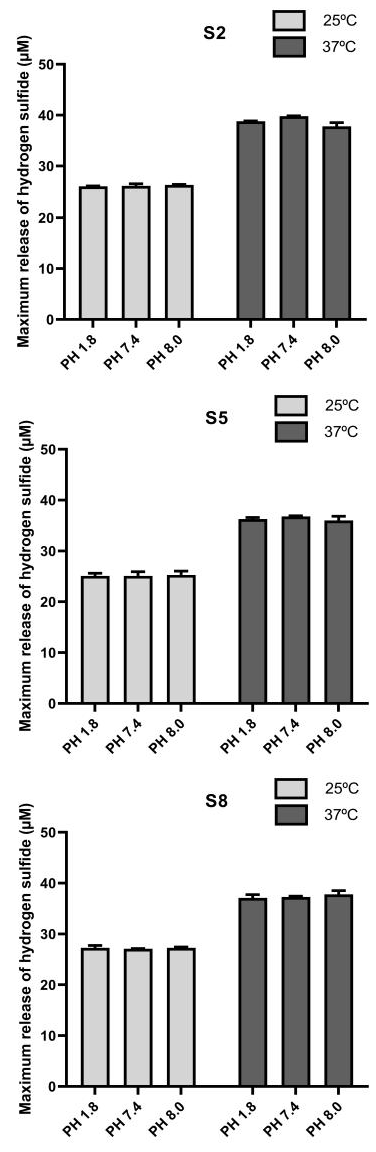

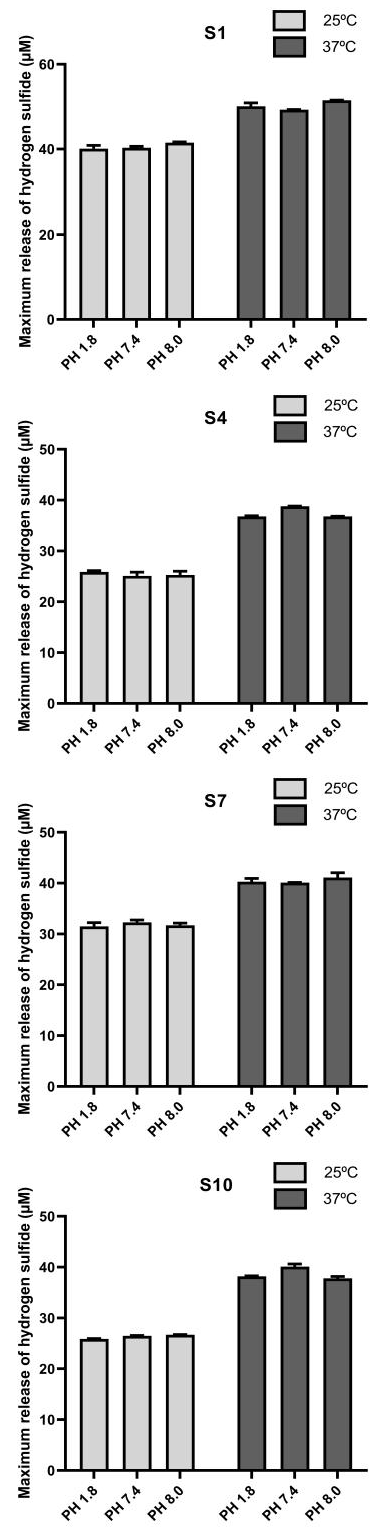

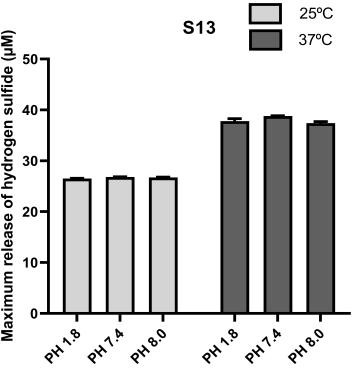


**Figure S1.** H2S released from the compounds. Each bar represents the mean ± SD of three independent experiments.

**H2S measurement**

A 5 mM solution of Na2S in sodium phosphate buffer (20 mM) was prepared (Na2S*9H2O, 120 mg in 100 mL volumetric flask) and used as the stock solution. Aliquots of 50, 100, 200, 400, 600, 800, 1000, 1500 μL of the Na2S stock solution were added into a 50 mL volumetric flask and dissolved in sodium phosphate buffer to obtain the standard solutions in 5, 10, 20, 40, 60, 80, 100, 150 μM, respectively. 1 mL aliquot of the respective solution was reacted with the methylene blue (MB+cocktail: 30 mM FeCl3 (200 mL) in 1.2 M HCl, 20 mM of N,N-dimethyl-1, 4-phenylenediamine sulfate (200 mL) in 7.2 M HCl, 1%w/v of Zn(OAc)2 (100 mL) in H2O at room temperature for at least 15 min (each reaction was performed in triplicate). The absorbance of methylene blue was measured at λmax = 670 nm in UV-Vis spectrophotometer. The Na2S calibration curve was obtained. The reaction was initiated by adding 75 μL of stock solution of the donor (40 mM, in THF) into phosphate buffer (30 mL) containing accelerator for TECP (1.0 mM) or L-Cysteine (1.0 mM). Then 2.0 mL of reaction aliquots were periodically taken and transferred to colorimetric cuvette containing zinc acetate (1% w/v, 200 mL) and *N,N*-dimethyl-1,4-phenylenediamine sulfate (20 mM, 400 mL) in 7.2M HCl and ferric chloride (30 mM, 400 mL) in 1.2M HCl. We adjust the pH of the solution by adjusting the buffer ratio. The absorbance (670 nm) of the resulted solution was determined 15 min thereafter using an UV-Vis spectrometer. The H2S concentration of each sample was calculated against a calibration curve of Na2S.

**Cell viability**

The CCK-8 assay is a commonly used method for assessing cell viability, based on cellular metabolic activity. The principle involves the use of CCK-8 reagent, which contains WST-8 (a water-soluble tetrazolium derivative), that produces a colored product upon reduction, indirectly reflecting cell viability. In brief, cells under investigation are seeded in a 6-well plate and divided into different drug treatment groups and control groups. After a specific period of treatment (24 hours), an appropriate amount of CCK-8 reagent is added to each group’s cell culture medium and thoroughly mixed to ensure uniform distribution within the cells. The culture plate is then incubated in a suitable incubator for a defined period (usually 4 hours), followed by measuring the absorbance of each group's culture medium using a spectrophotometer, typically at a wavelength of 450 nanometers. Cell relative activity or viability is calculated based on absorbance values, often by subtracting the background absorbance from the control group's values and normalizing to the control group's values to obtain relative activity or viability.

**Nitrite level detection**

The Griess assay is used to measure nitrite levels in biological samples, both *in vivo* and *in vitro*. It is commonly employed in biological and medical research related to inflammation, oxidative stress, and serves as a common indicator for NO (nitric oxide). Initially, cell culture media treated with different concentrations of drugs are placed in a 96-well plate, and the Griess reagent is added in proportion to initiate a reaction between nitrites and the Griess reagent, resulting in the formation of a red azo compound. The absorbance is measured at a wavelength of 540-550 nanometers, and the concentration of the red product is directly proportional to the absorbance. Nitrite concentrations can be calculated using a standard curve.

**Detection of cytokine levels**

In this experiment, the levels of TNF-α, IL-10, and HO-1 cell factors were assessed using the ELISA method. Cell lysis was performed using RIPA buffer to collect proteins from cells treated with different compound concentrations. The protein concentration of the samples was then measured using the BCA assay and diluted to 0.1 mg/ml. A series of standard samples with known concentrations were prepared, and the test samples, standard samples, and negative controls were added to pre-coated ELISA plate wells in specified proportions. The plate was sealed and incubated at 4℃ for 8 hours to allow sample adsorption. After adsorption, the plate was washed to remove unbound samples and impurities. Specific detection antibodies were added to form immune complexes with the target proteins. After incubation for 0.5 hours, TMB enzyme substrate was added, followed by stop solution to terminate the enzyme reaction. The absorbance of each well at 562 nanometers wavelength was measured using an ELISA reader, and the concentrations of cell factors in the test samples were calculated based on the standard curve.

**In vitro PI3K and ATK enzyme inhibitory assay**

The inhibitory activity of **S11** on PI3K and ATK enzymes was assayed using the PI3K Assay Kit and the ATK Kit. PI3K enzymes were diluted with 2.5-fold kinase assay buffer according to the manufacturer's instructions to obtain 4 ng/ml. experiments were performed in 96-well plates as follows: 5 mL of PIP2 substrate was added to each well, followed by 5 μL of inhibitor buffer from each inhibitor, and inhibitor buffer was added to the positive controls and blanks. Add 5 uL of 12.5 mM ATP. 10 mL of diluted PI3K enzyme was added to the positive control and inhibitor wells to initiate the reaction. Shake the plate carefully and incubate for 40 min at 30°C. After 40 min of incubation, add 25 mL of ADP-Glo reagent to each well. Cover the plate with aluminium foil and incubate for another 45 min at room temperature. Add 50 mL of kinase detection reagent per well. Cover the plate with aluminium foil and incubate for an additional 30 min at room temperature. Measure luminescence with a microplate reader. A range of (0.1 ~ 100 mM) of the tested compound **S11** was prepared and the experiment was repeated three times. The results were expressed as IC_50_ values (concentration required for 50% inhibition). Dose response curves and linear regression equations were used to calculate the IC_50_ values. PI-103 were used as positive control.

**Docking and Collecting Data**

Selection of previously described 7F7W targets for molecular docking with S11. The files of sdf. structure of S11 active ingredient compounds were downloaded from PubChem database, and imported into ChemBio3D 14.0 software to adjust the spatial conformation of active ingredients, calculate the optimization of energy, and save in mol2 format. After AutoDockTools processing, the files were saved in pdbqt format. The three-dimensional crystal structure of the target protein was downloaded from the PDB protein database (https://www.rcsb.org/). The PDBID was 7F7W. The water molecule and organic matter in the target protein were removed by Notedad2, and then the target protein was imported into AutoDockTools for hydrogenation, charge distribution, and atomic type addition. The pdbqt format file was saved. AutoDockVina was used for molecular docking, and the docking results were plotted with Pymol.

**Spectral data**


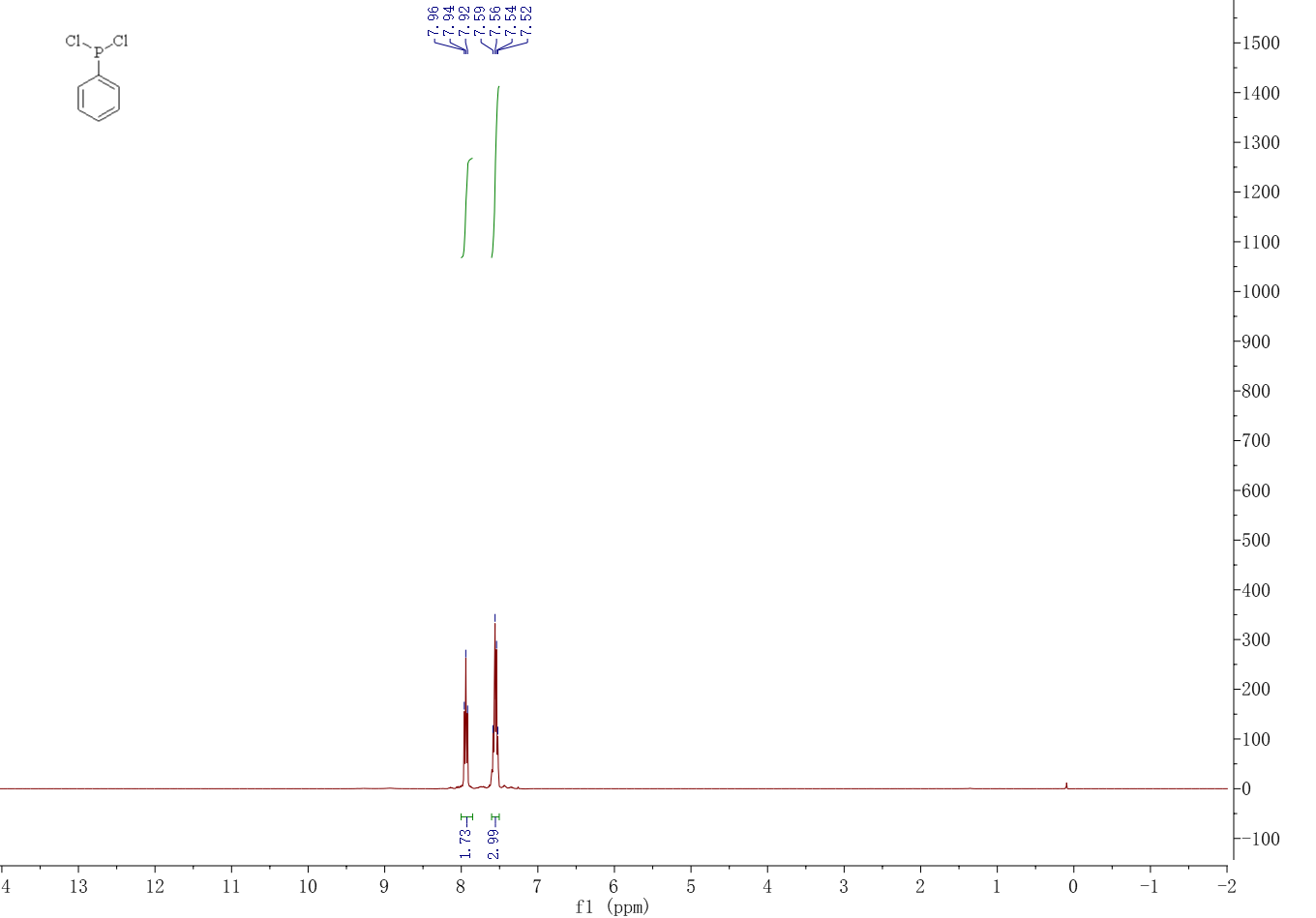


Fig 1. *^1^H NMR of* **B** (400 MHz, CDCl_3_)


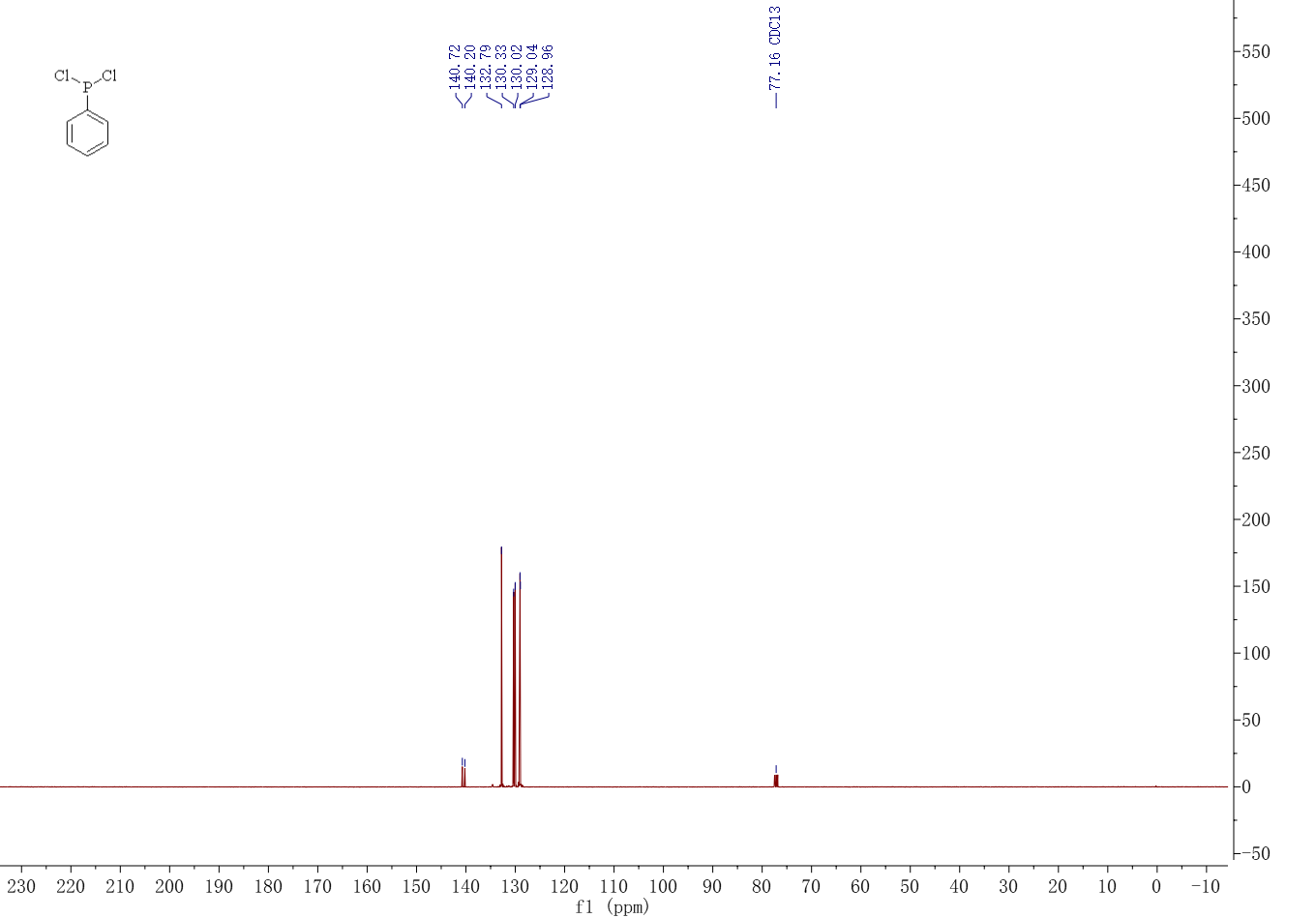


Fig 2. *^13^C NMR of* **B** (101 MHz, CDCl_3_)


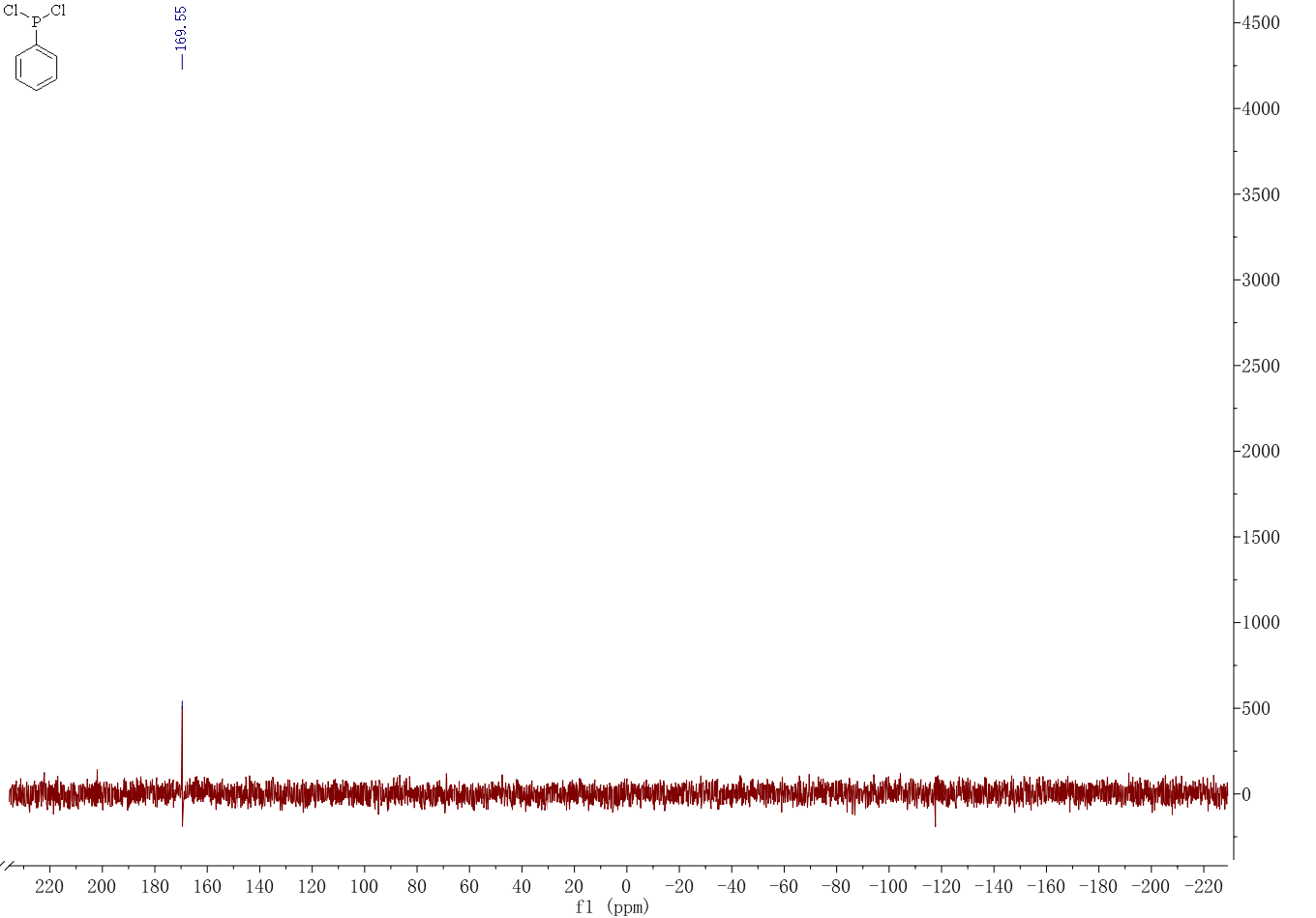


Fig 3. *^31^P NMR of* **B** (162 MHz, CDCl_3_)


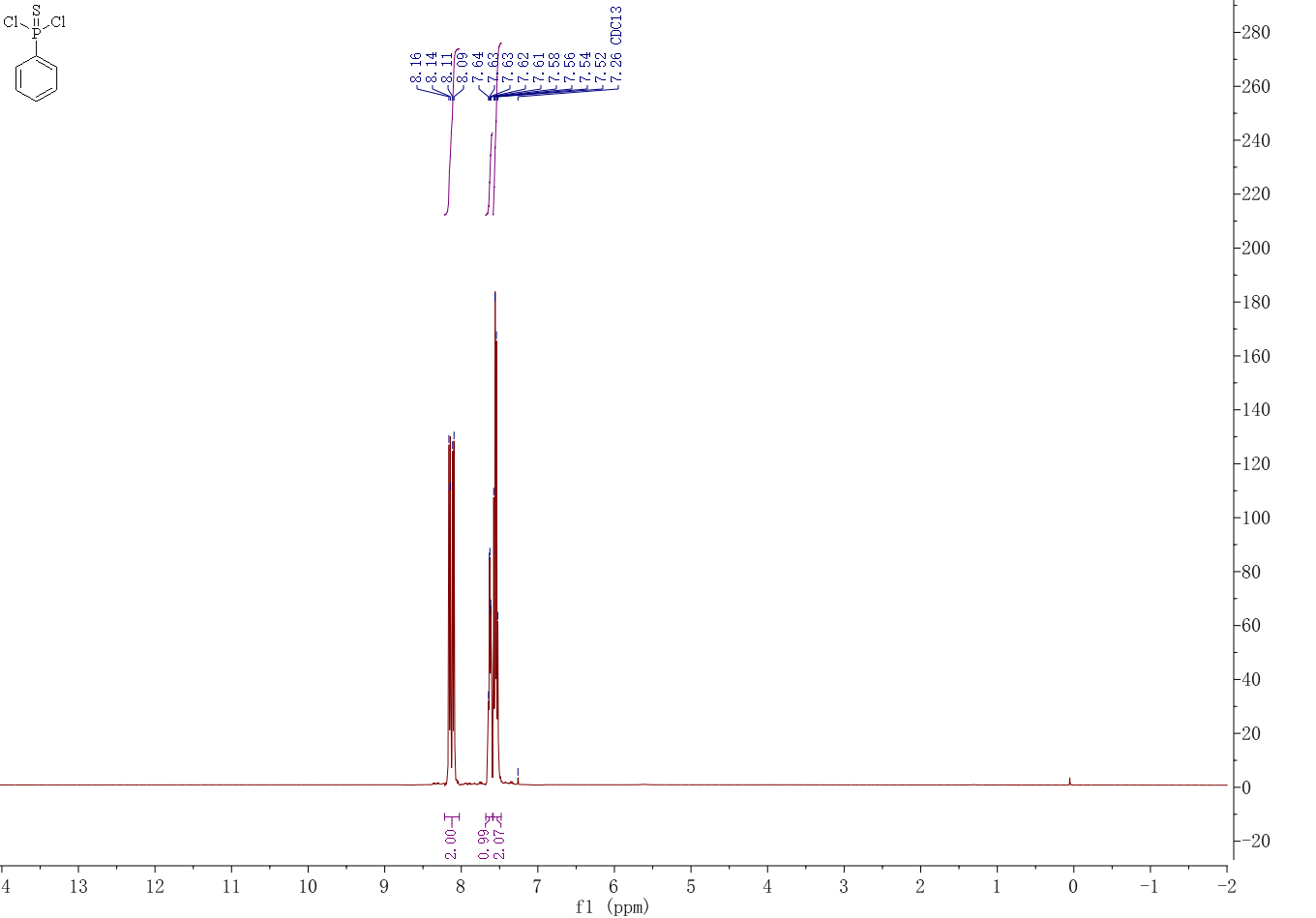


Fig 4. *^1^H NMR of* **S1** (400 MHz, CDCl_3_)


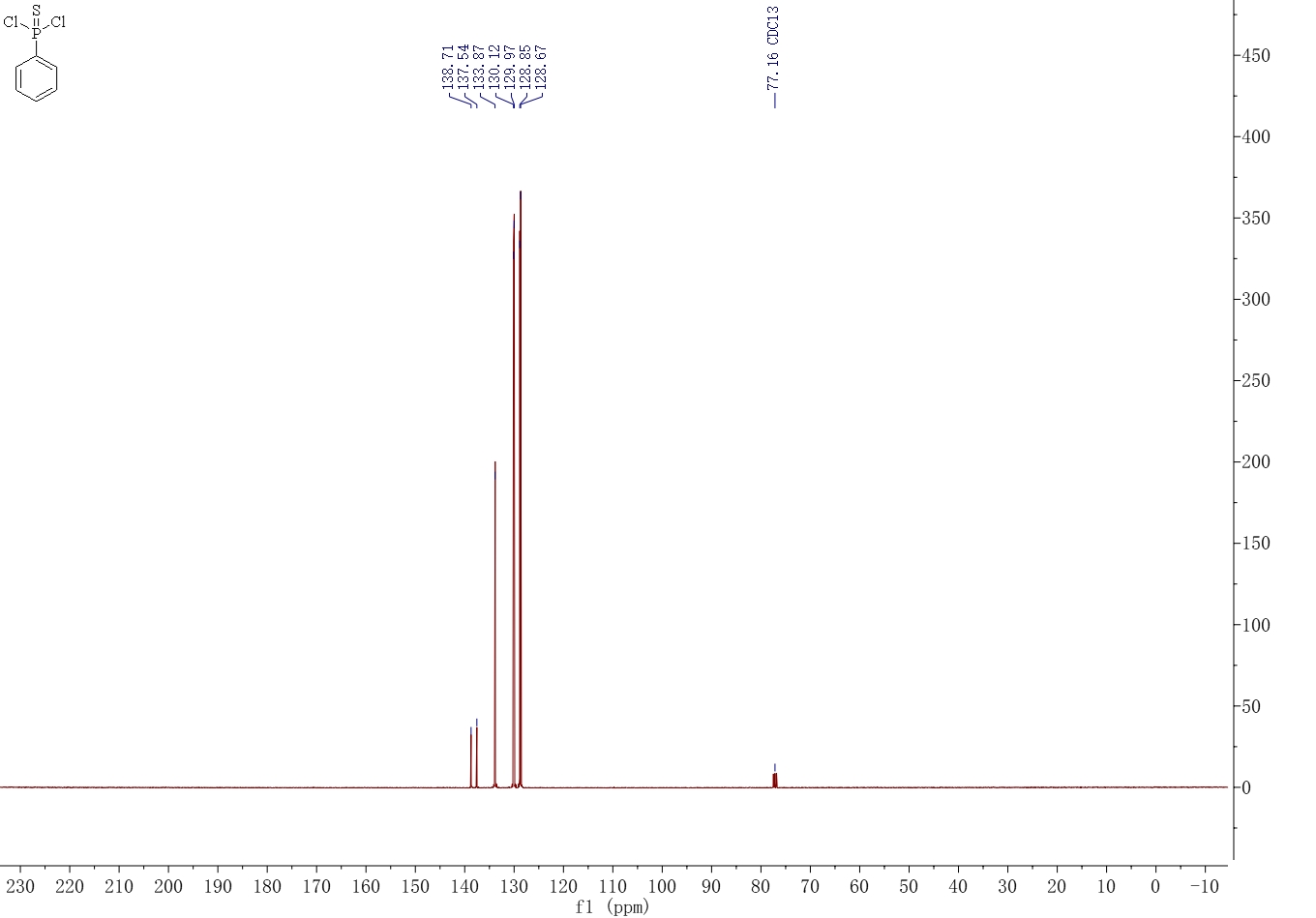


Fig 5. *^13^C NMR of* **S1** (101 MHz, CDCl_3_)


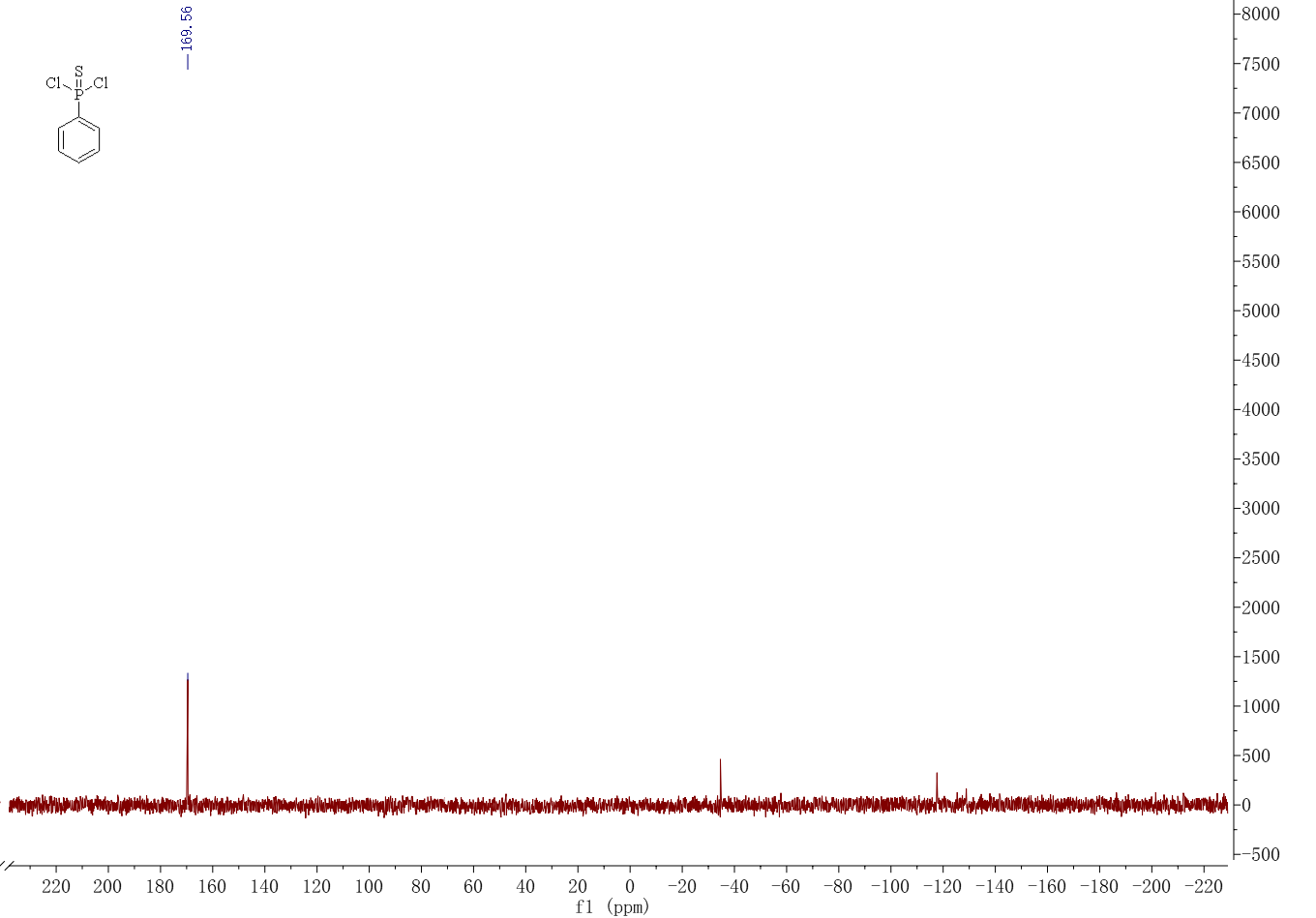


Fig 6. *^31^P NMR of* **S1** (162 MHz, CDCl_3_)


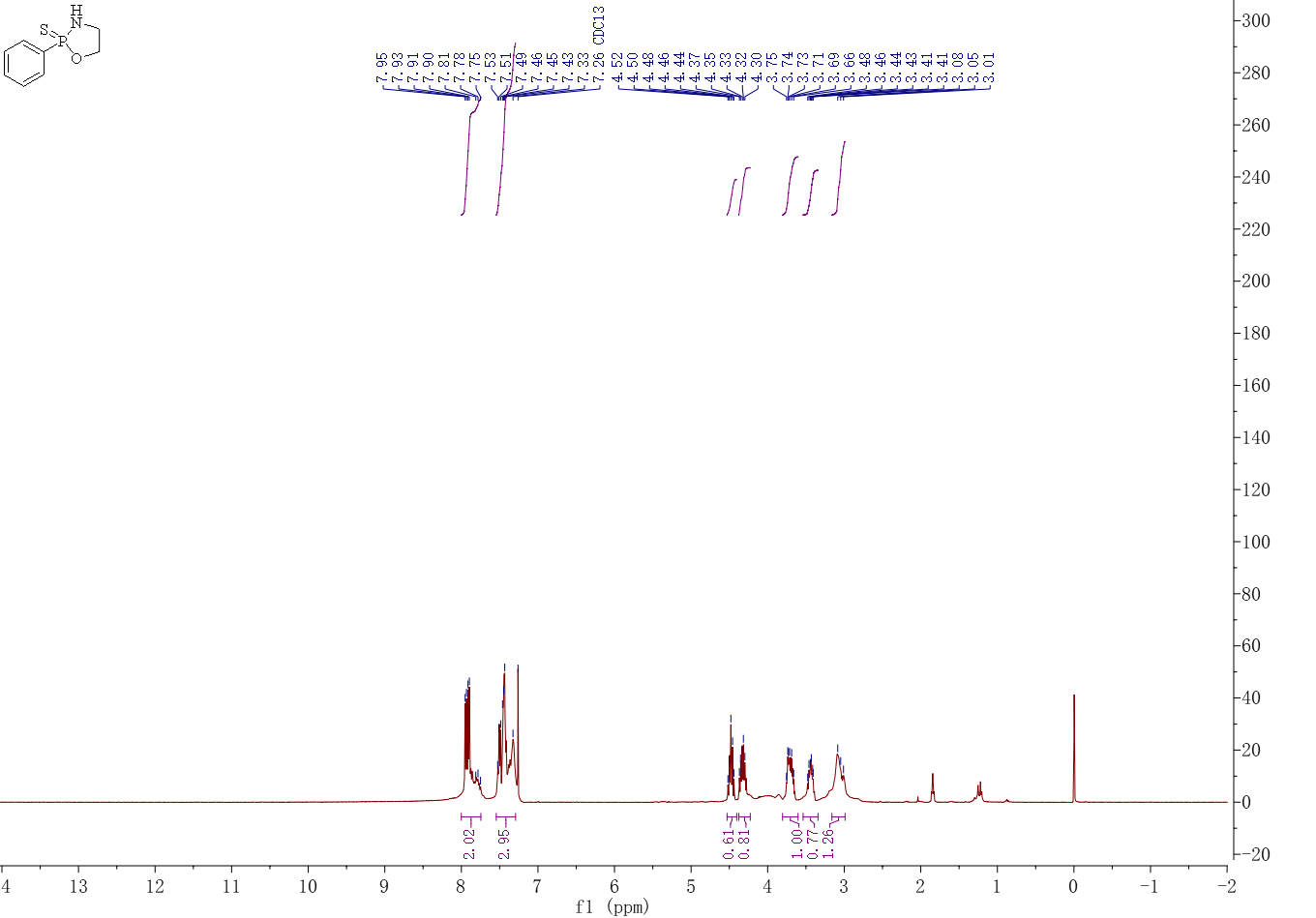


Fig 7. *^1^H NMR of* **S2** (400 MHz, CDCl_3_)


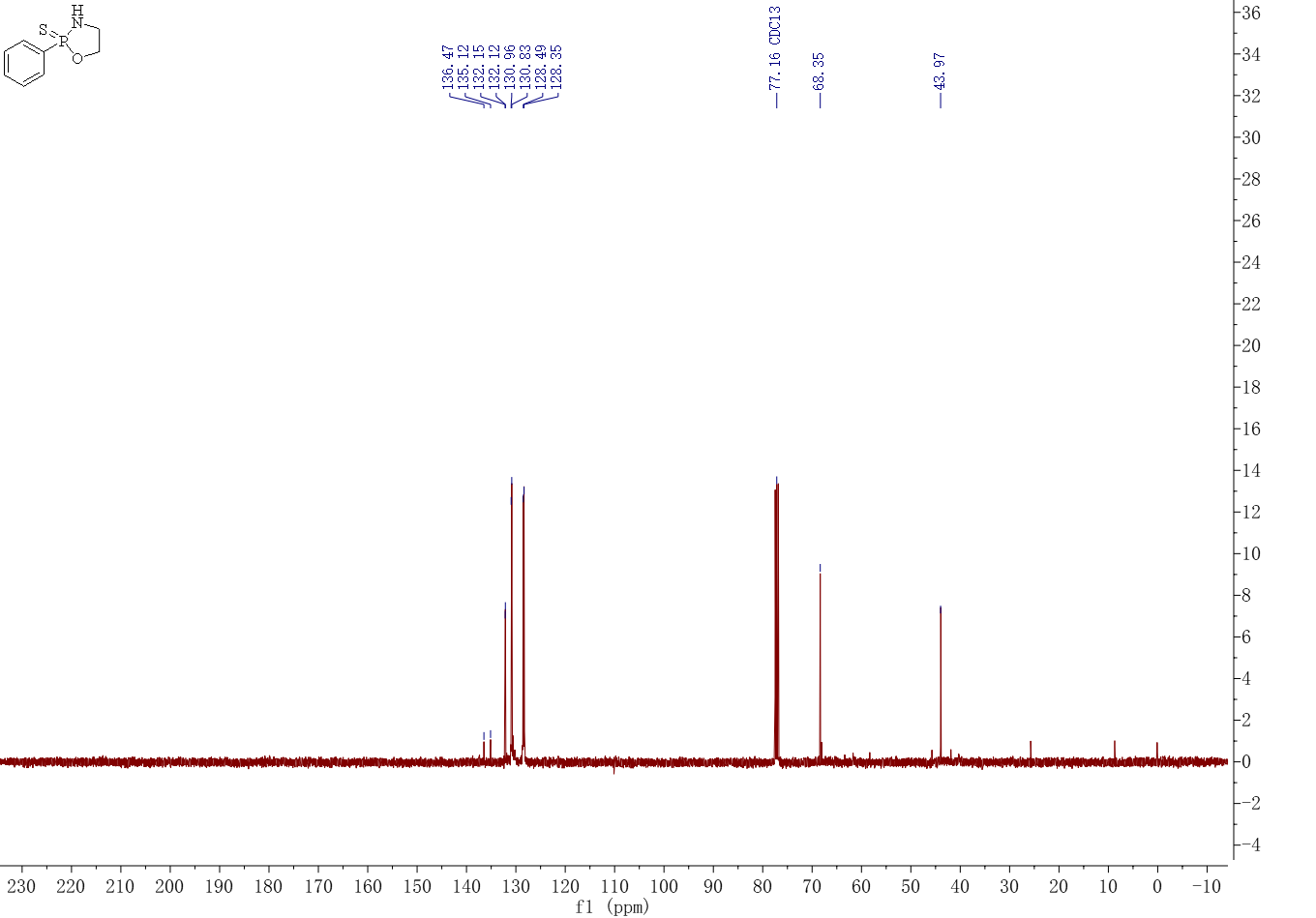


Fig 8. *^13^C NMR of* **S2** (101 MHz, CDCl_3_)


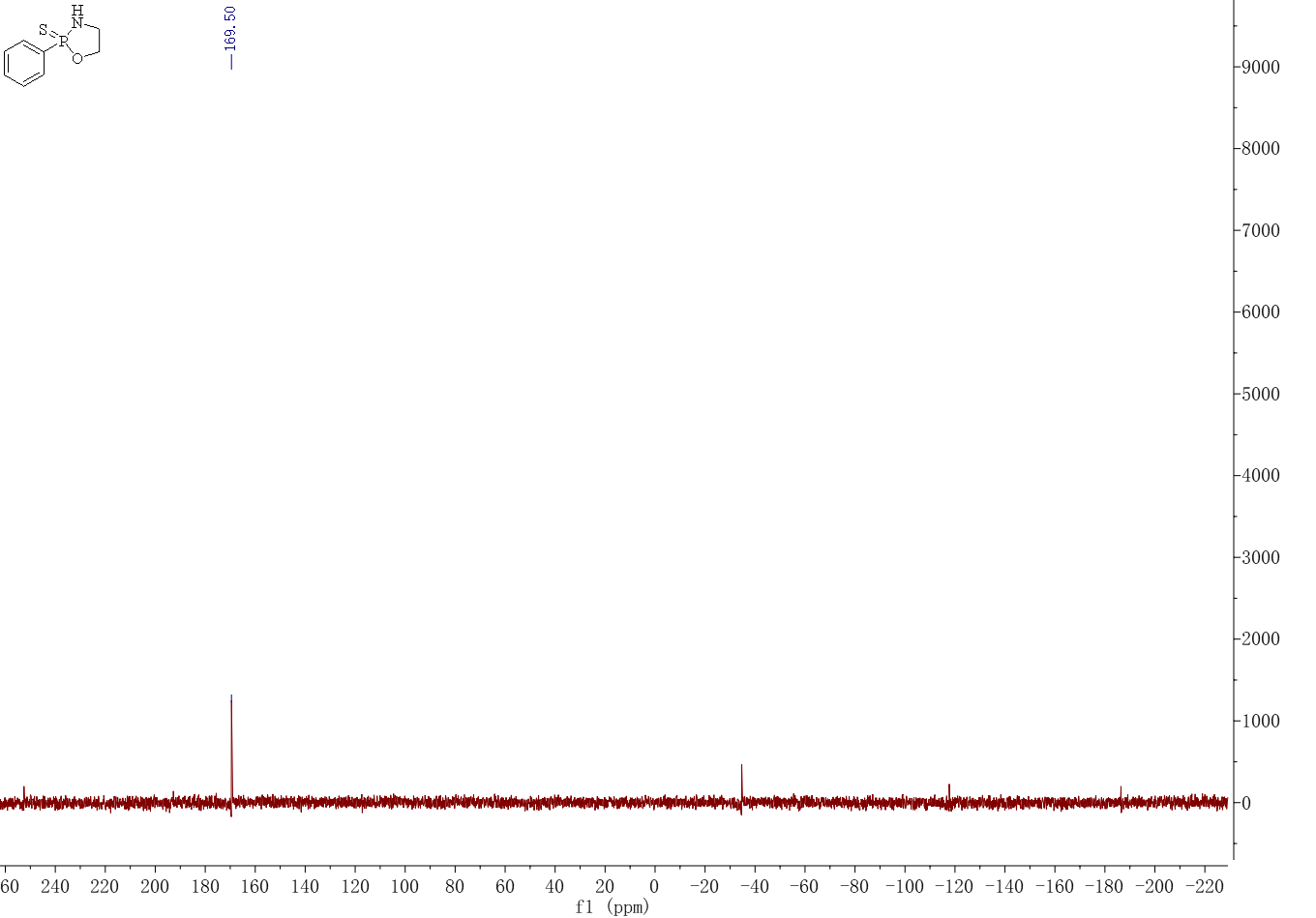


Fig 9. *^31^P NMR of* **S2** (162 MHz, DMSO)


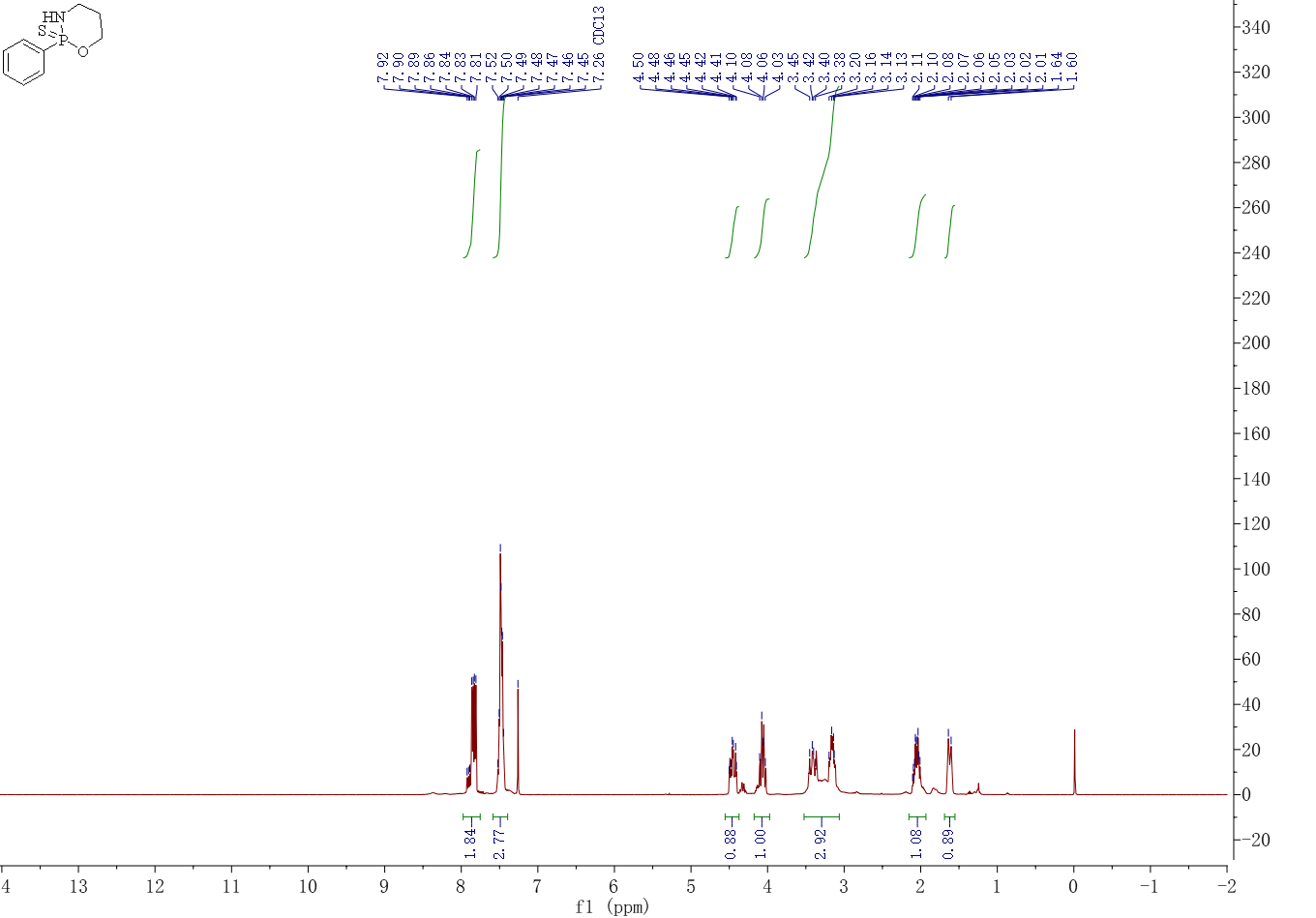


Fig 10. *^1^H NMR of* **S3** (400 MHz, CDCl_3_)


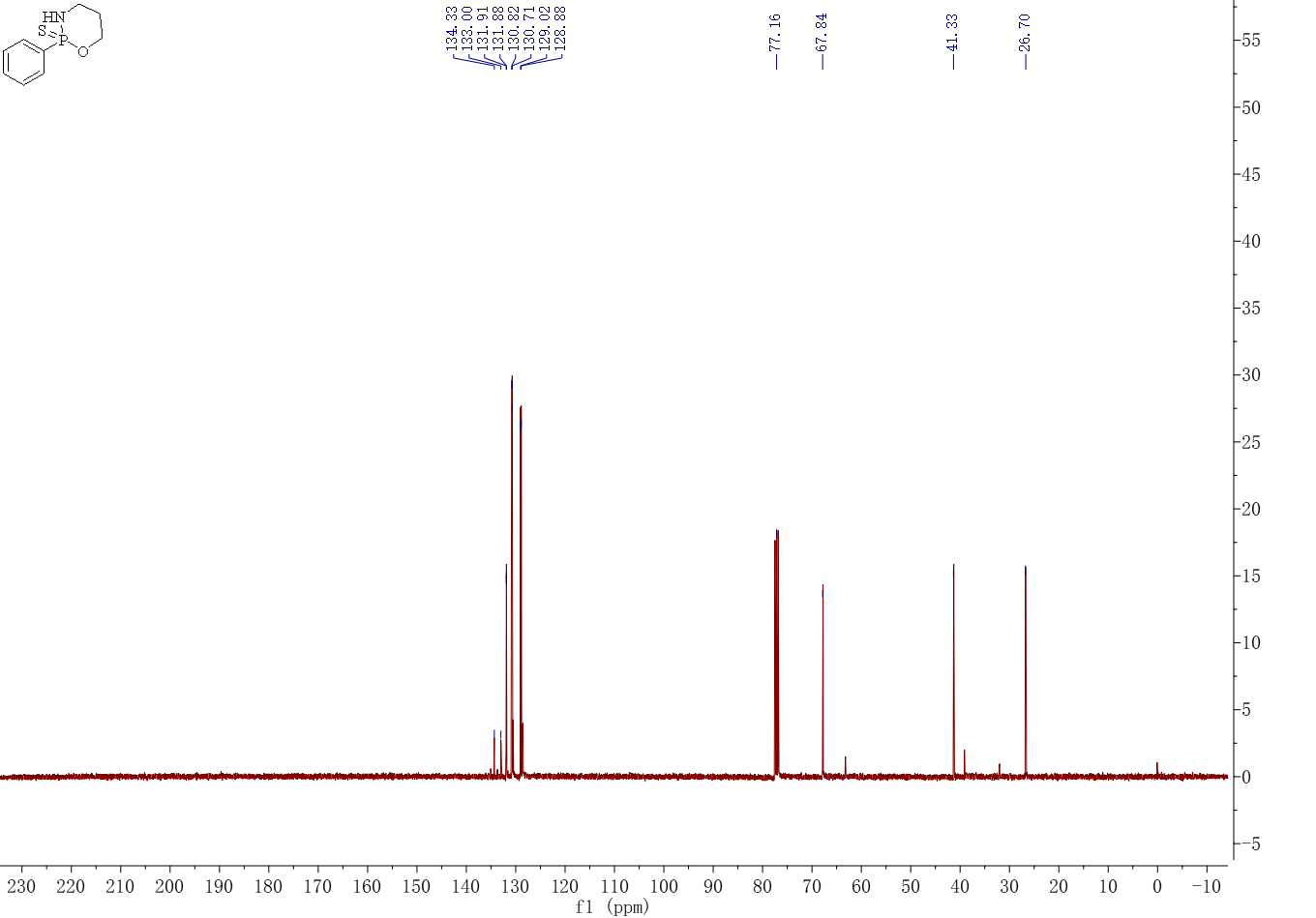


Fig 11. *^13^C NMR of* **S3** (101 MHz, CDCl_3_)


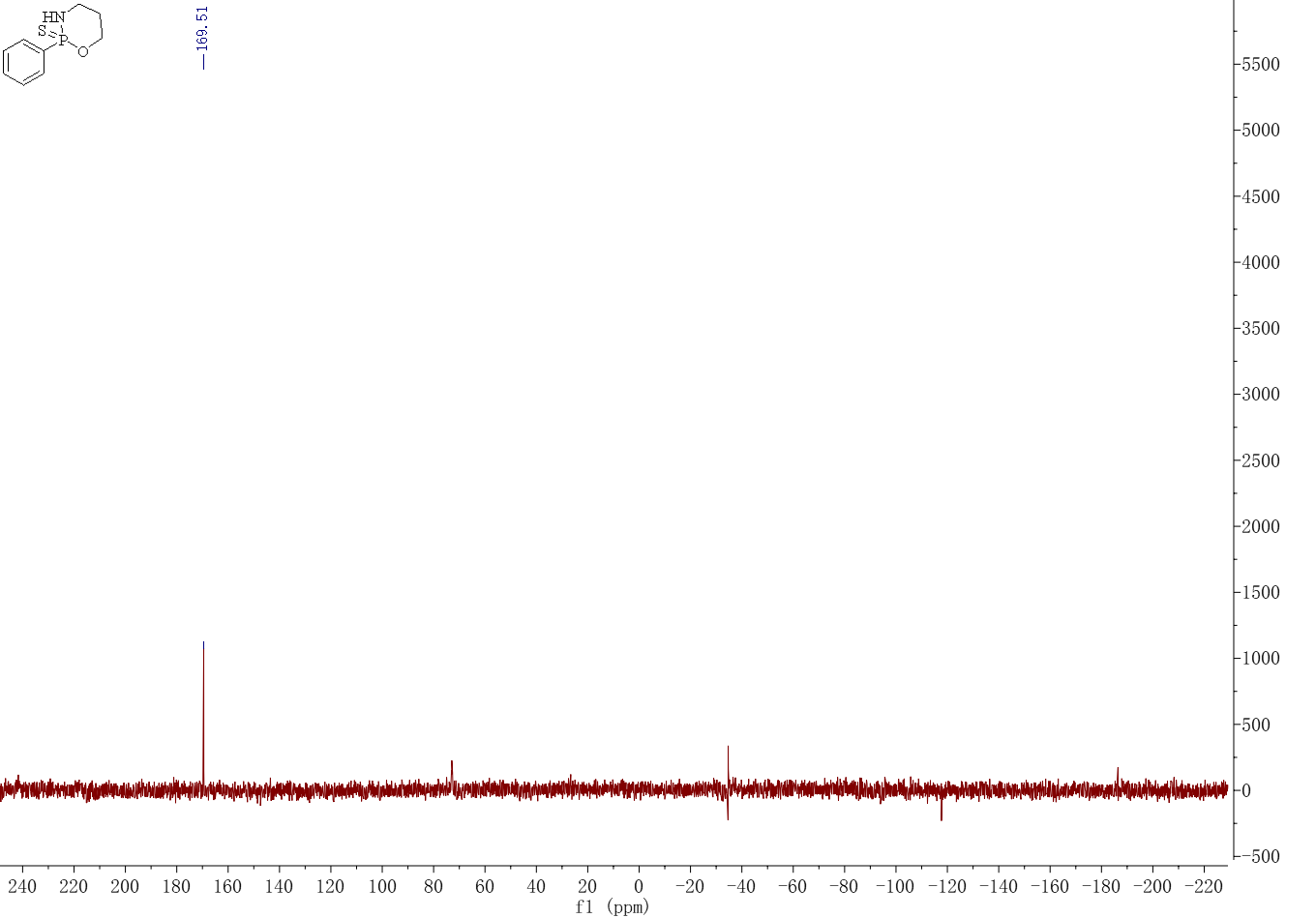


Fig 12. *^31^P NMR of* **S3** (162 MHz, DMSO)


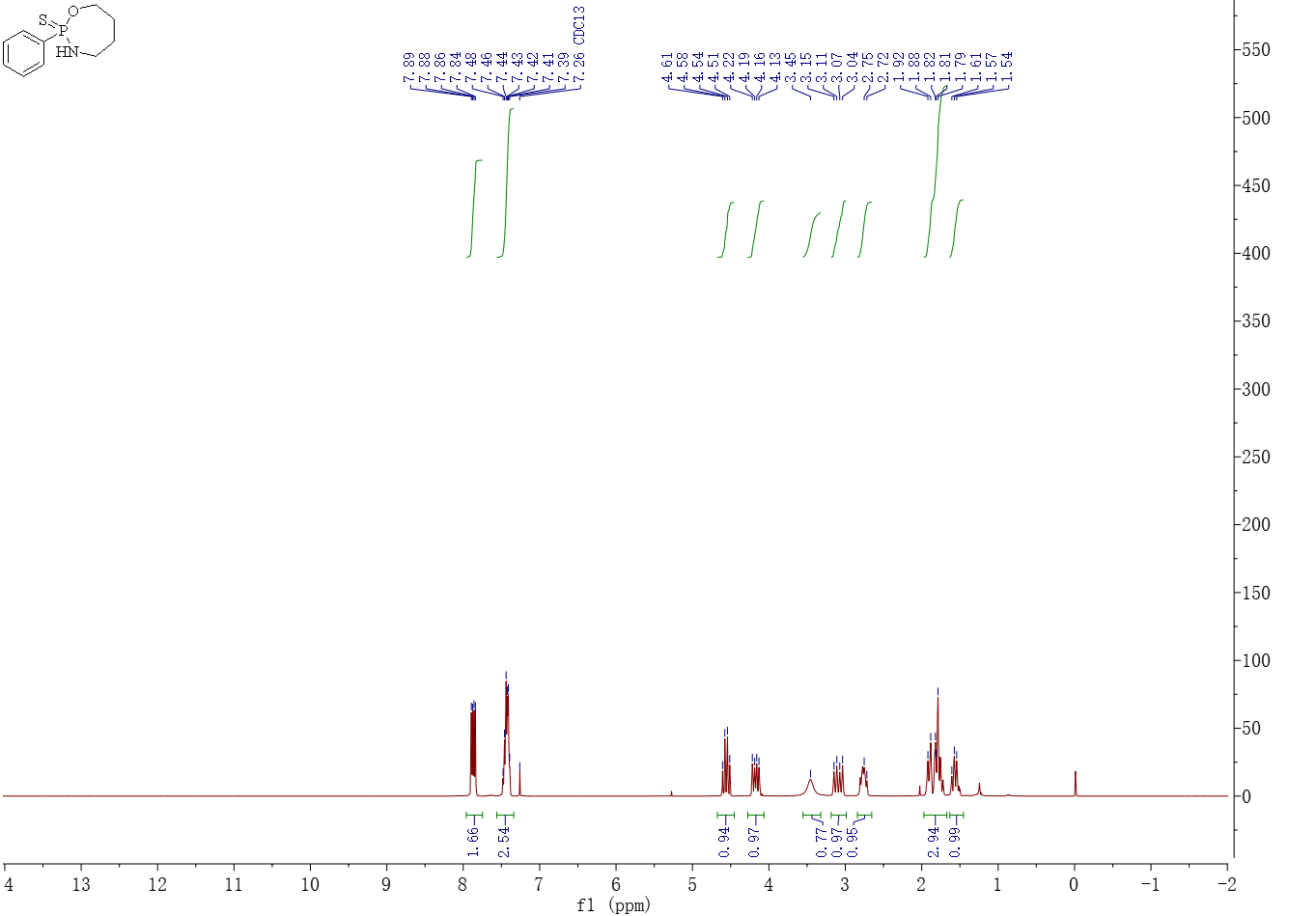


Fig 13. *^1^H NMR of* **S4** (400 MHz, CDCl_3_)


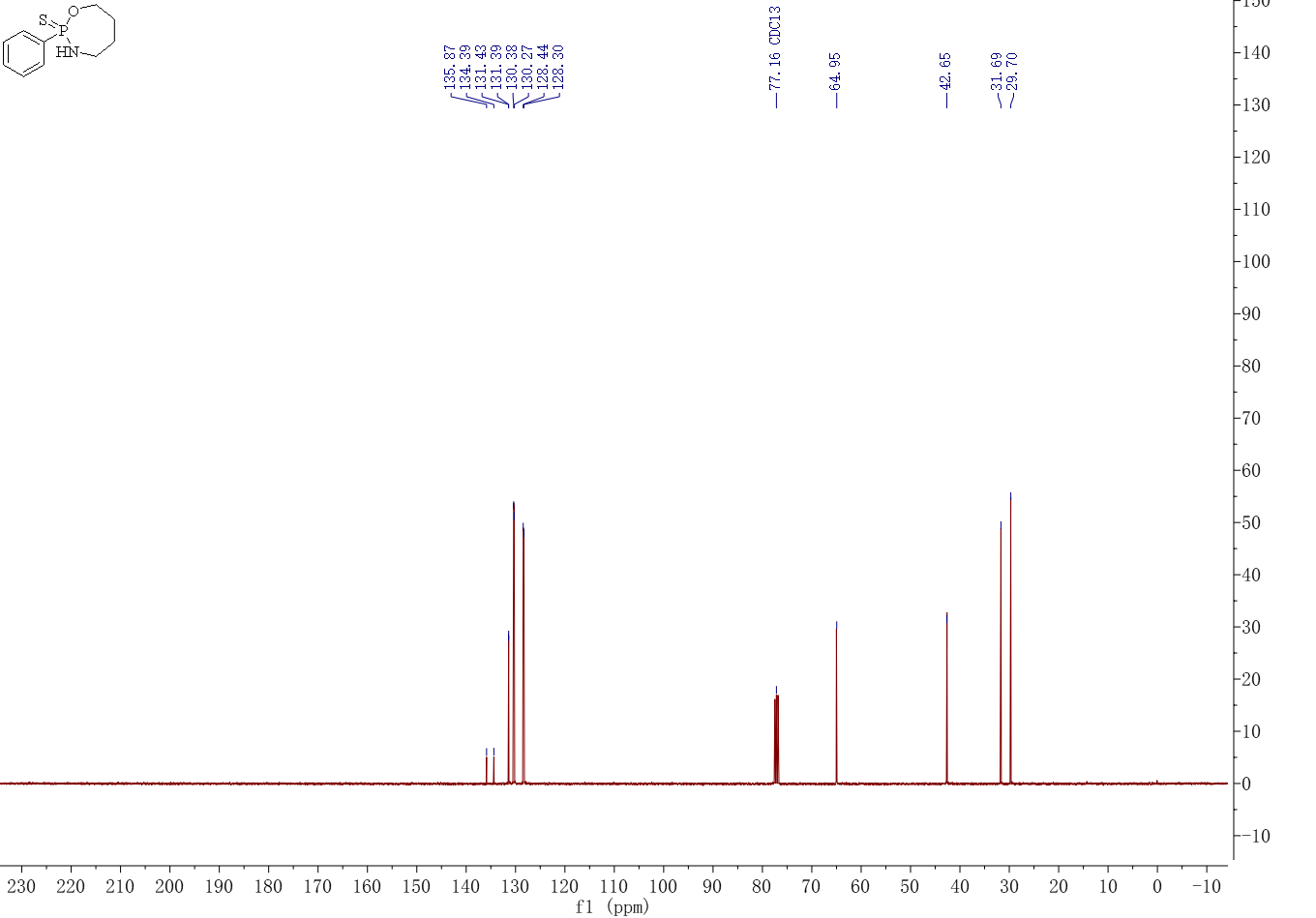


Fig 14. *^13^C NMR of* **S4** (101 MHz, CDCl_3_)


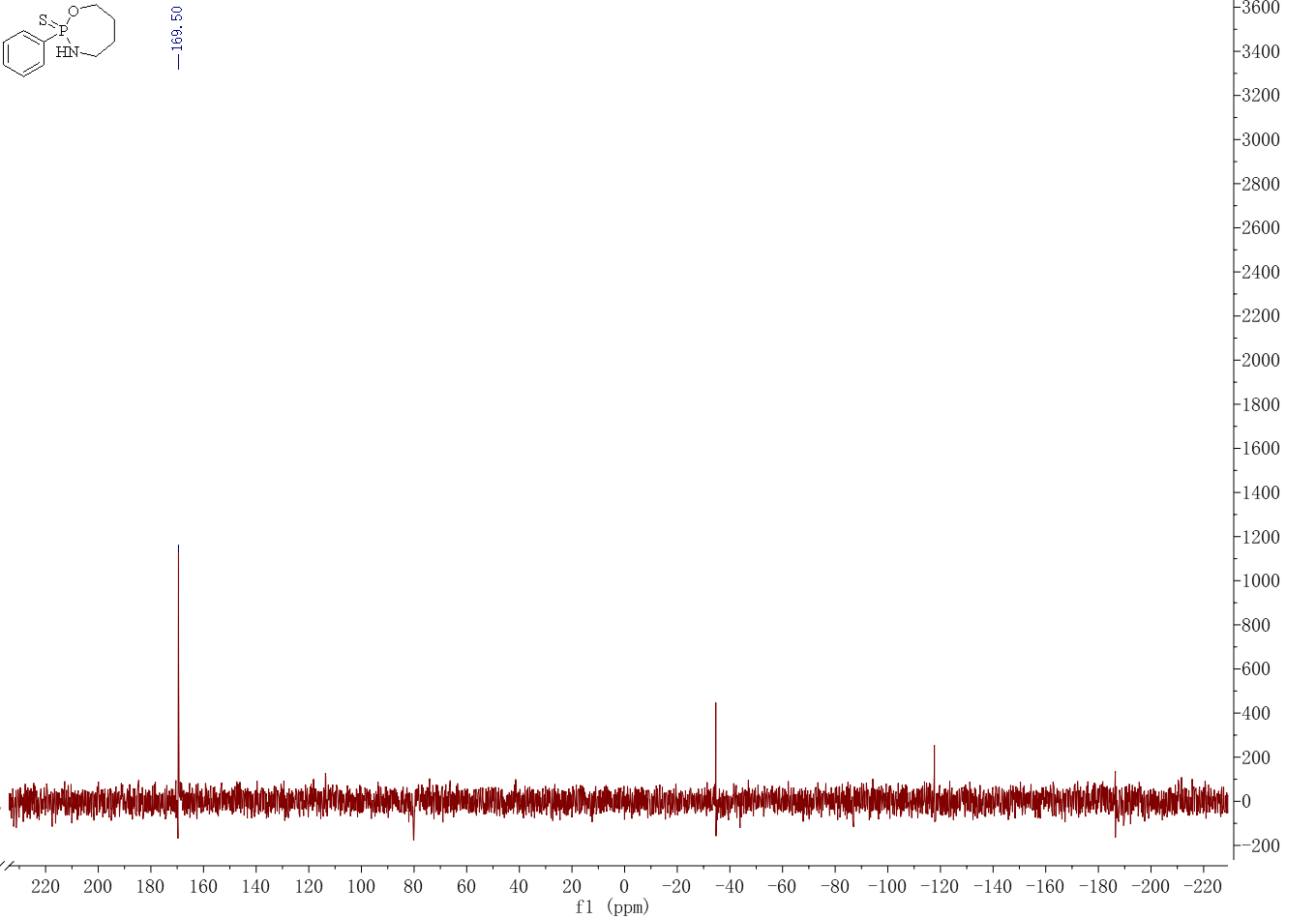


Fig 15. *^31^P NMR of* **S4** (162 MHz, DMSO)


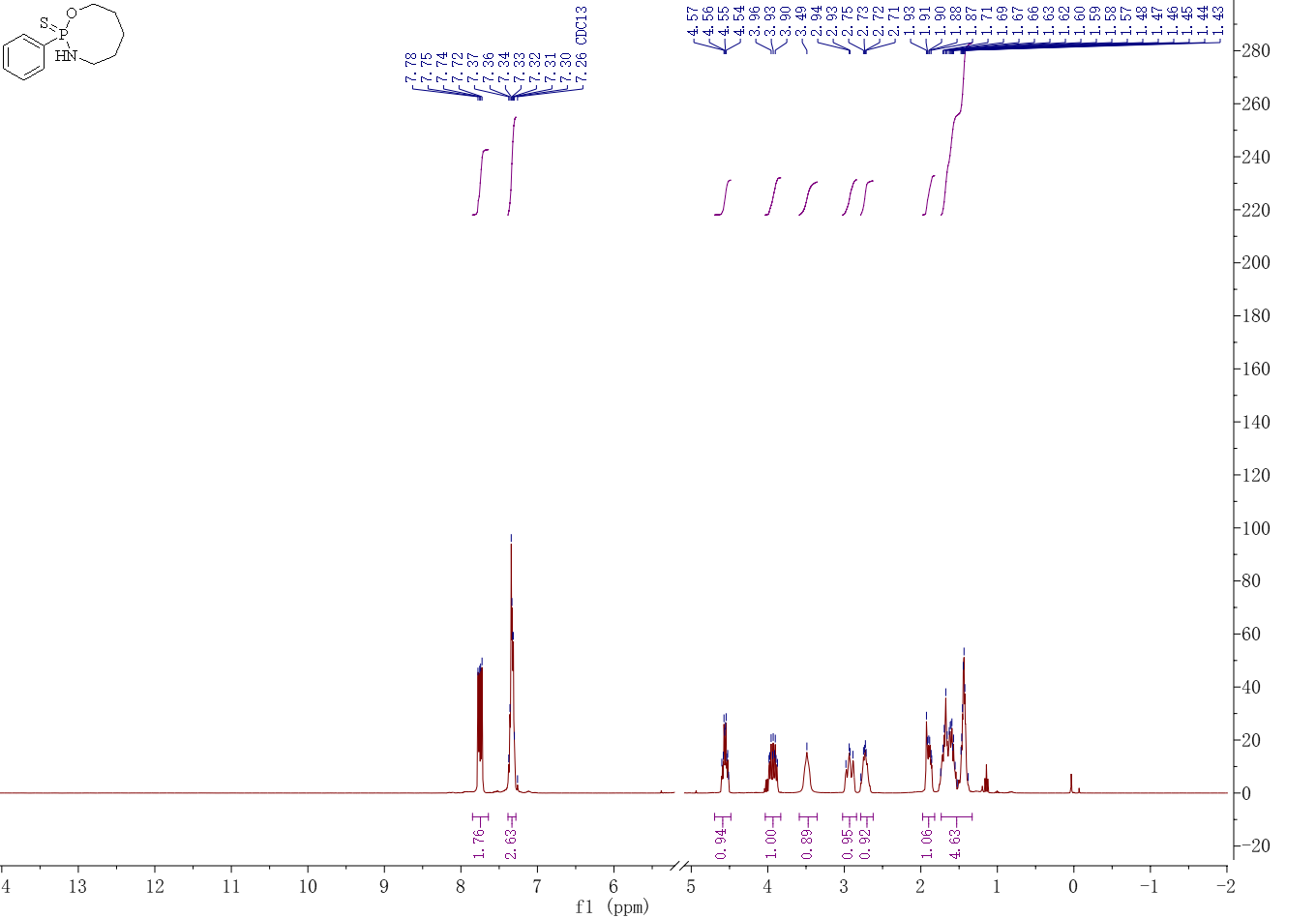


Fig 16. *^1^H NMR of* **S5** (400 MHz, CDCl_3_)


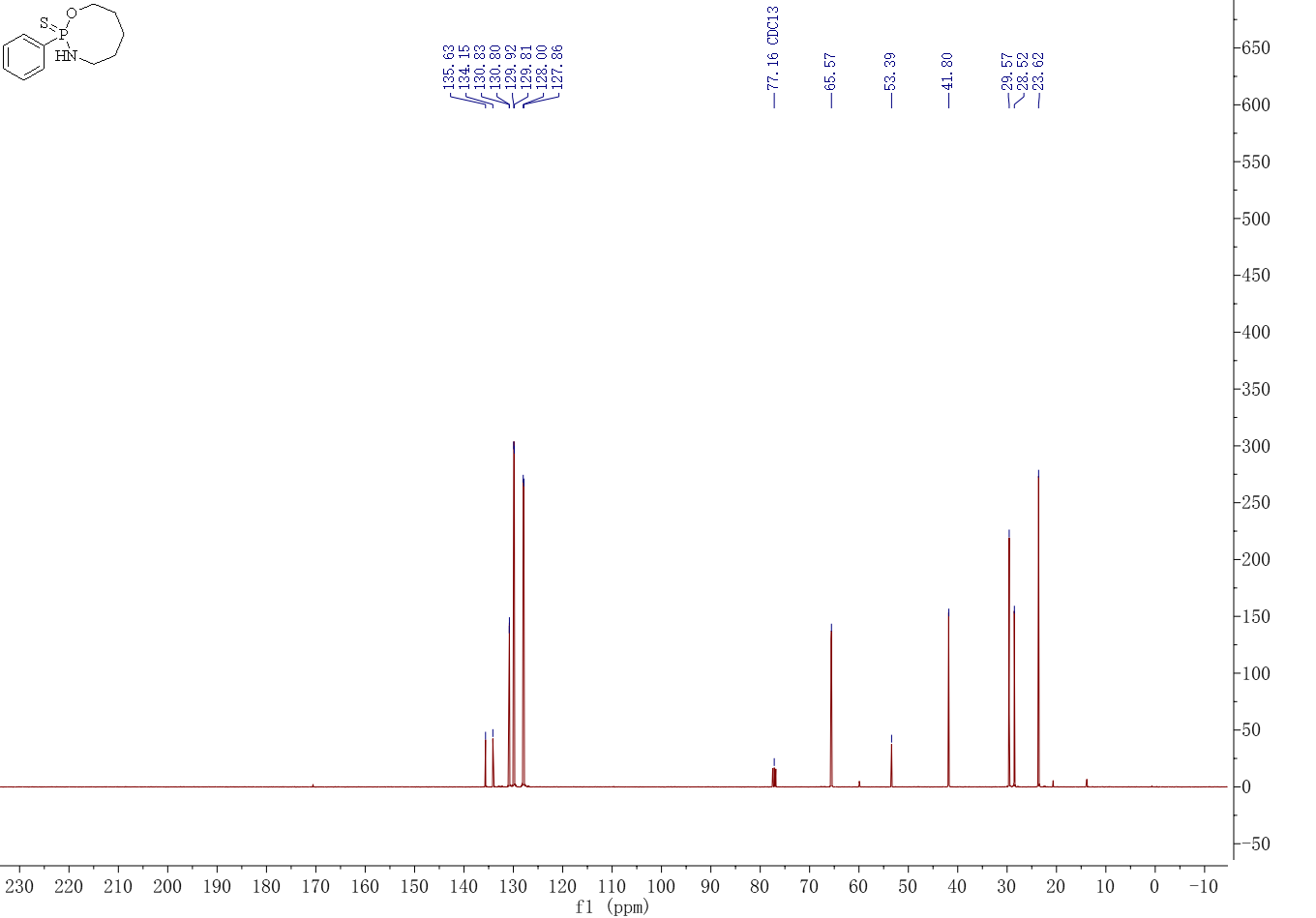


Fig 17. *^13^C NMR of* **S5** (101 MHz, CDCl_3_)


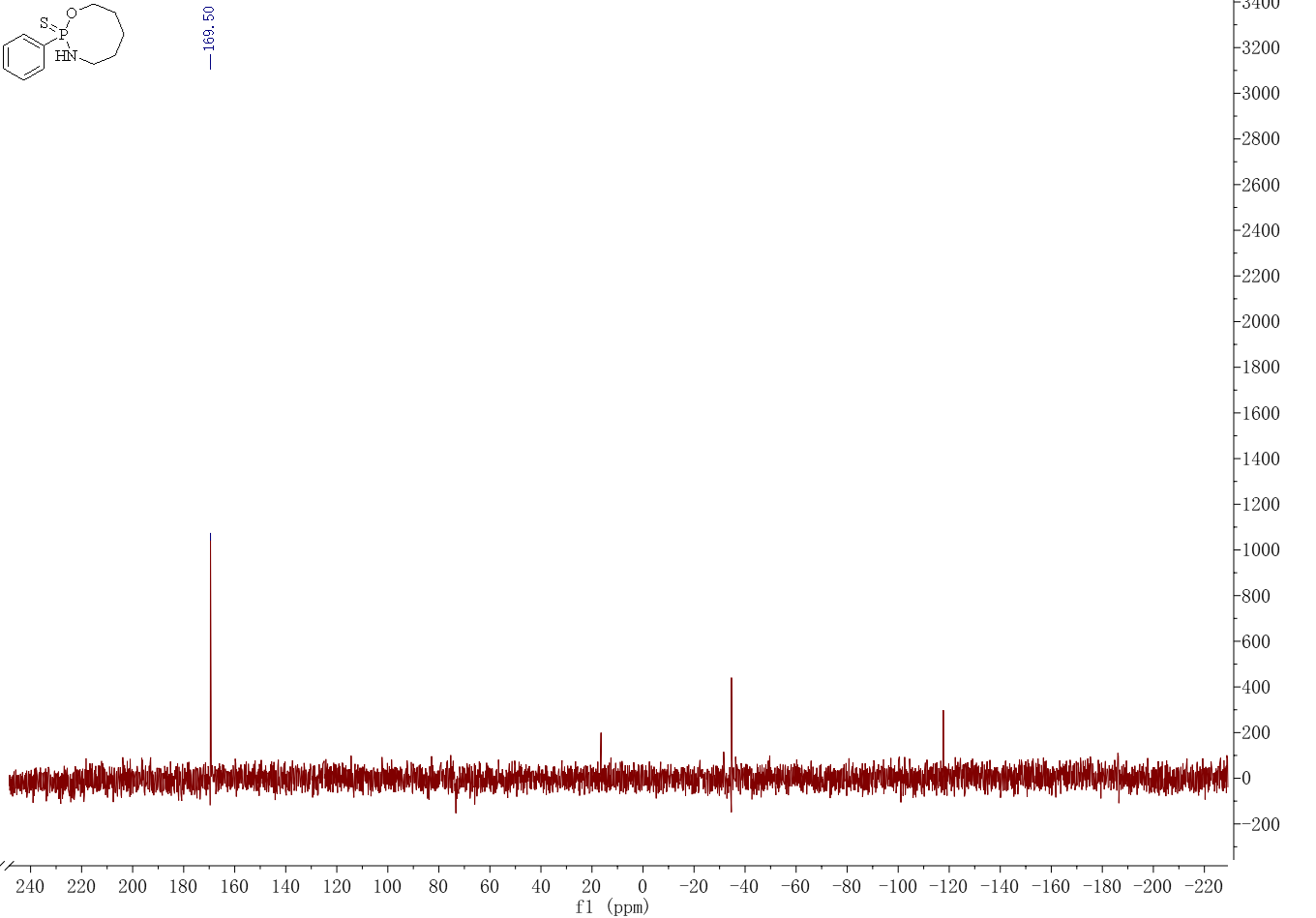


Fig 18. *^31^P NMR of* **S5** (162 MHz, DMSO)


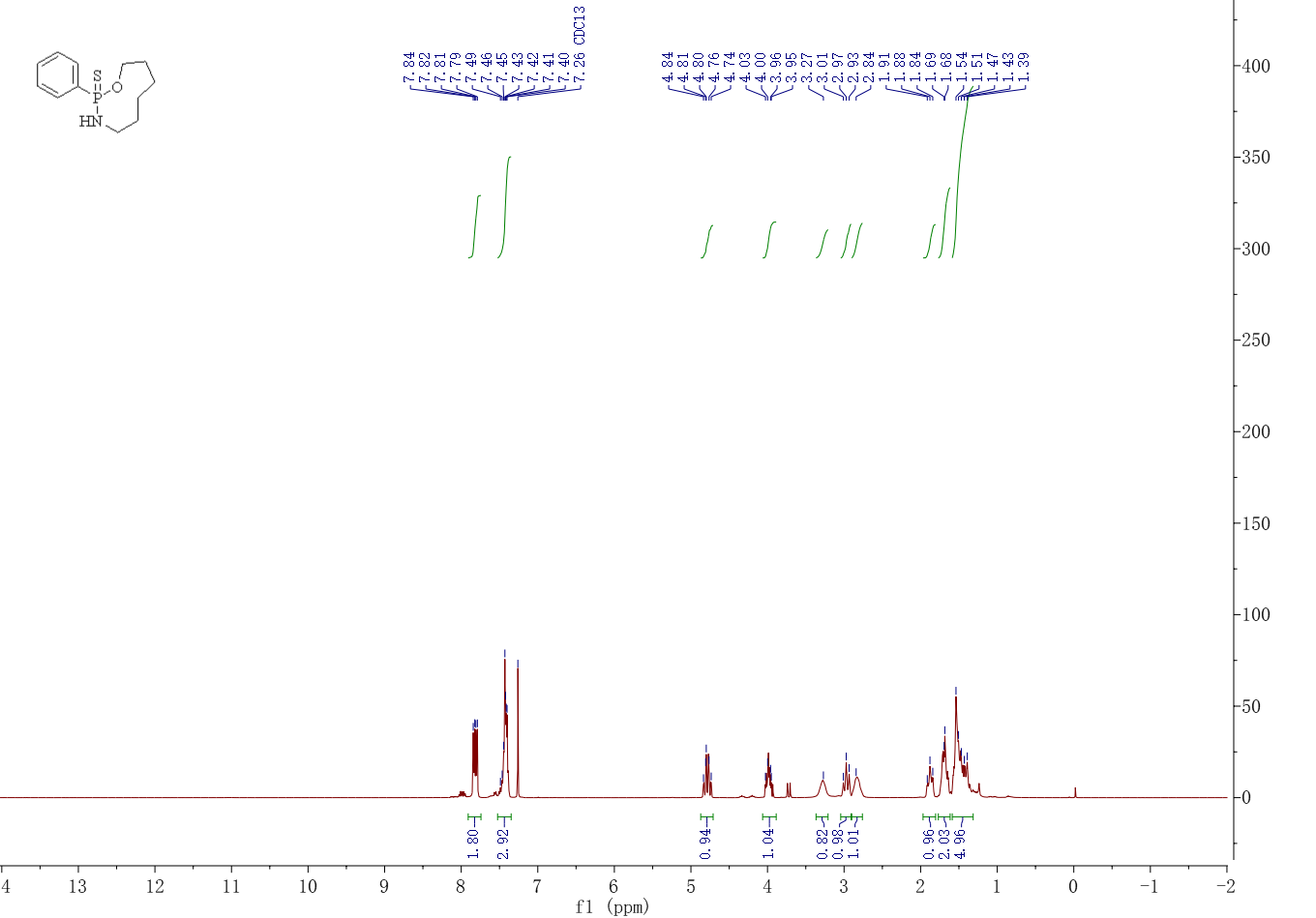


Fig 19. *^1^H NMR of* **S6** (400 MHz, CDCl_3_)


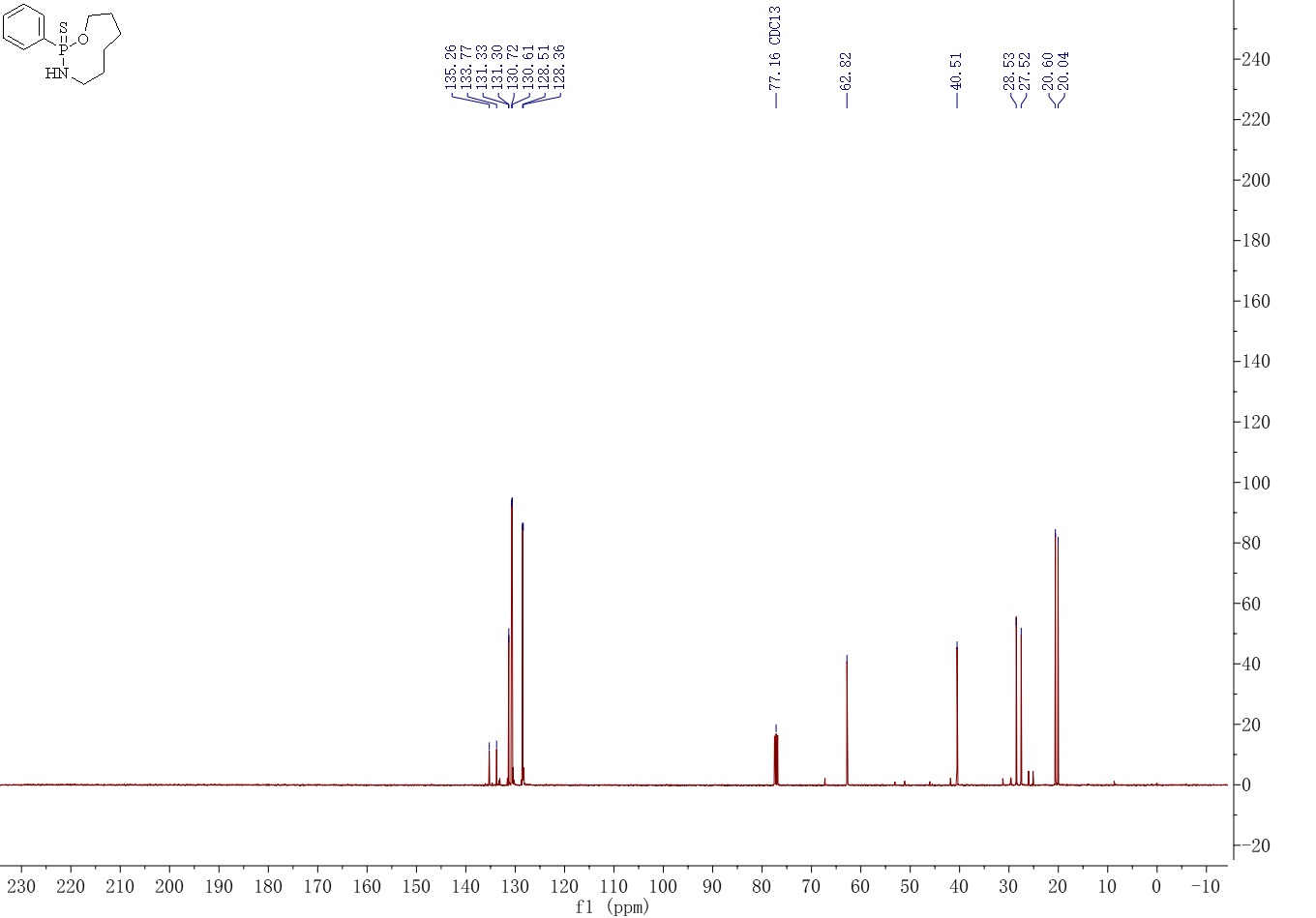


Fig 20. *^13^C NMR of* **S6** (101 MHz, CDCl_3_)


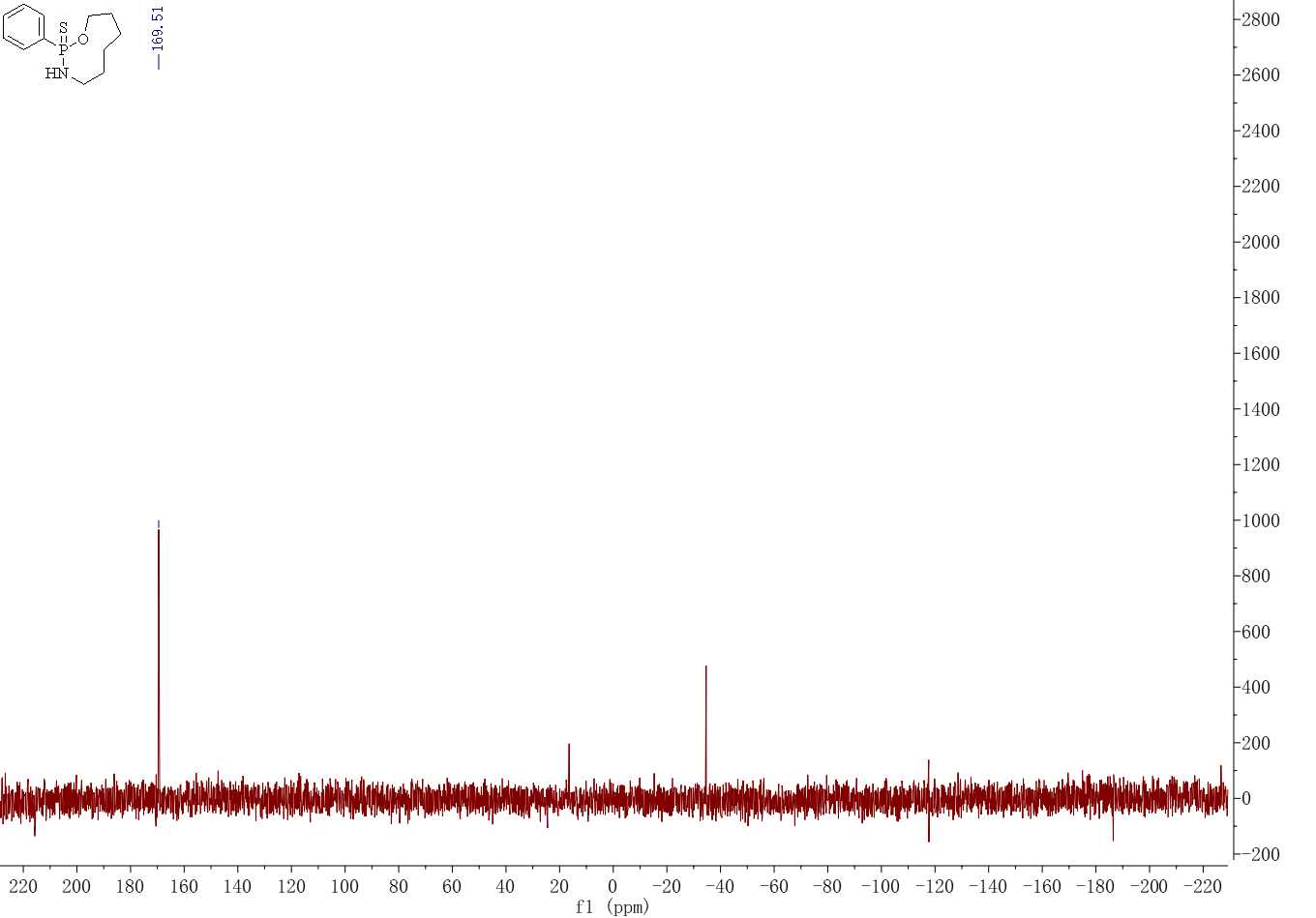


Fig 21. *^31^P NMR of* **S6** (162 MHz, DMSO)


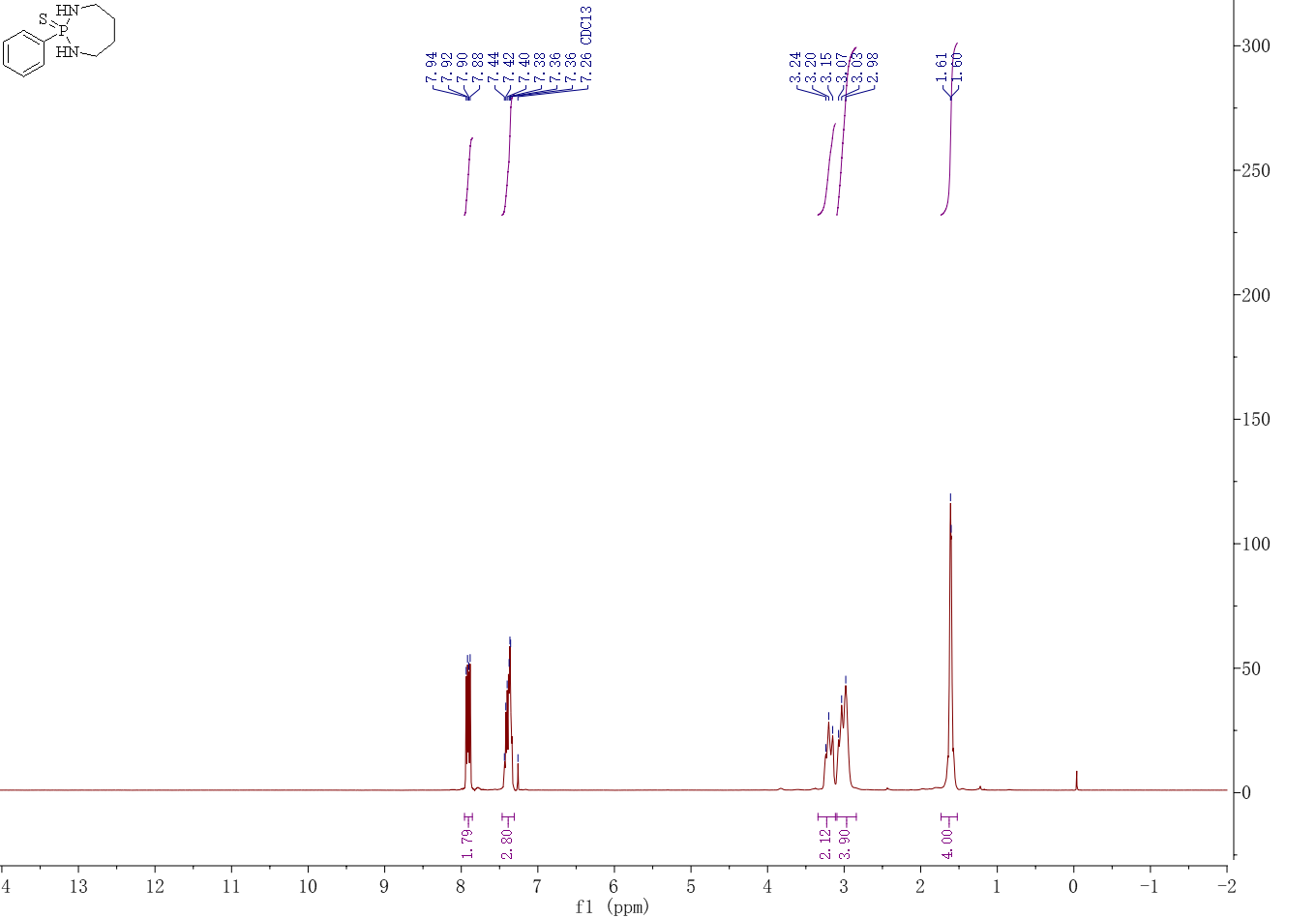


Fig 22. *^1^H NMR of* **S7** (400 MHz, CDCl_3_)


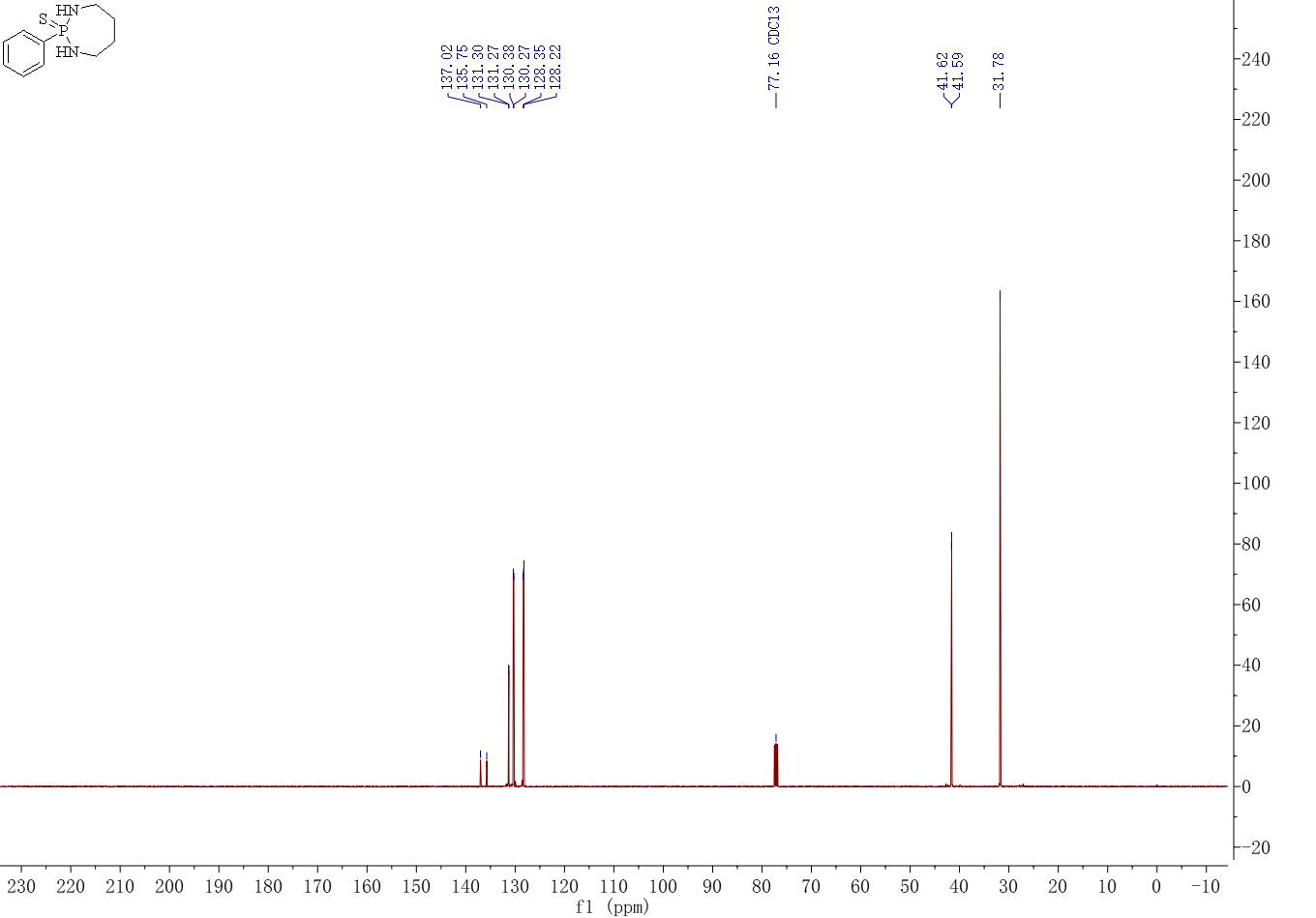


Fig 23. *^13^C NMR of* **S7** (101 MHz, CDCl_3_)


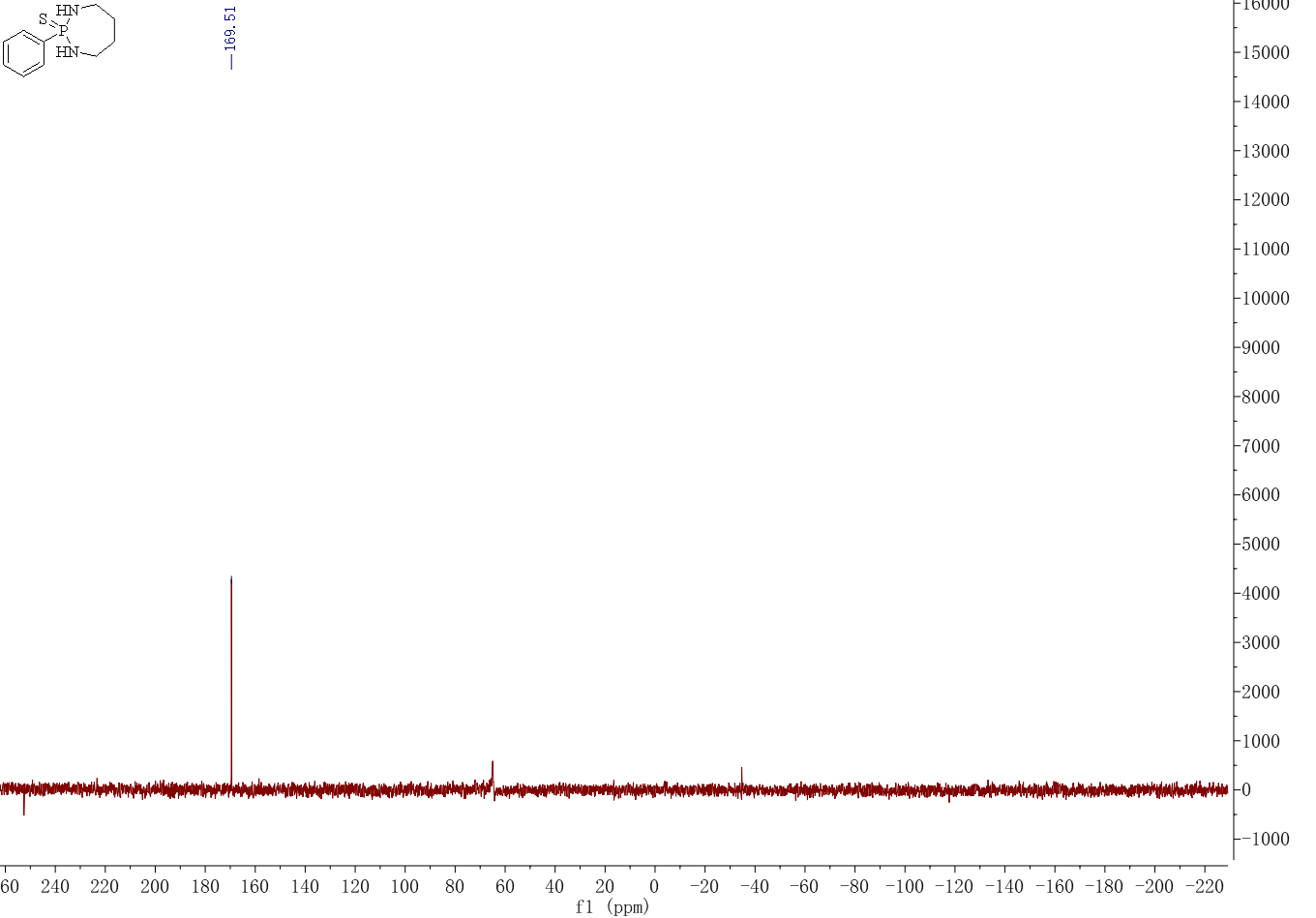


Fig 24. *^31^P NMR of* **S7** (162 MHz, DMSO)


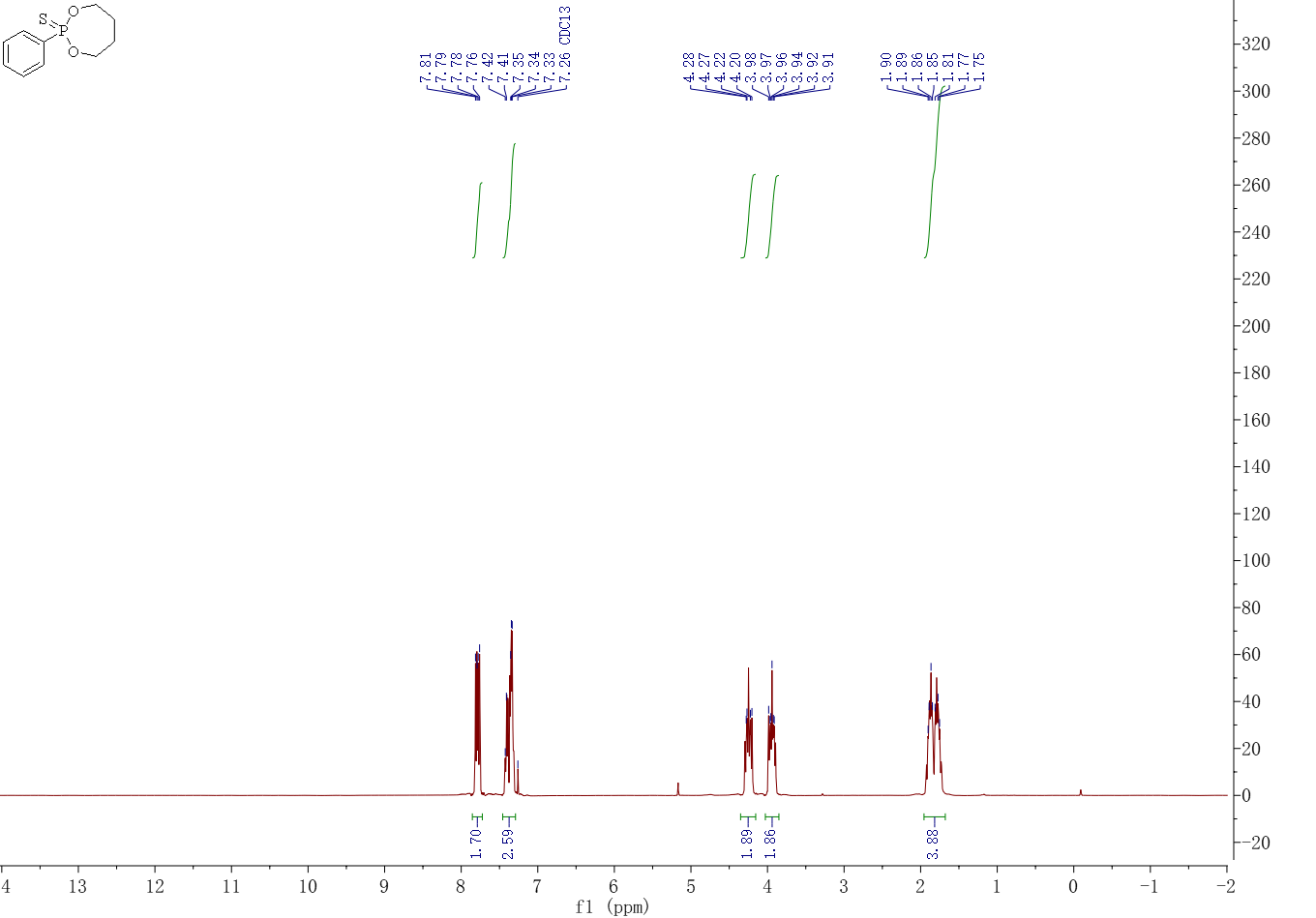


Fig 25. *^1^H NMR of* **S8** (400 MHz, CDCl_3_)


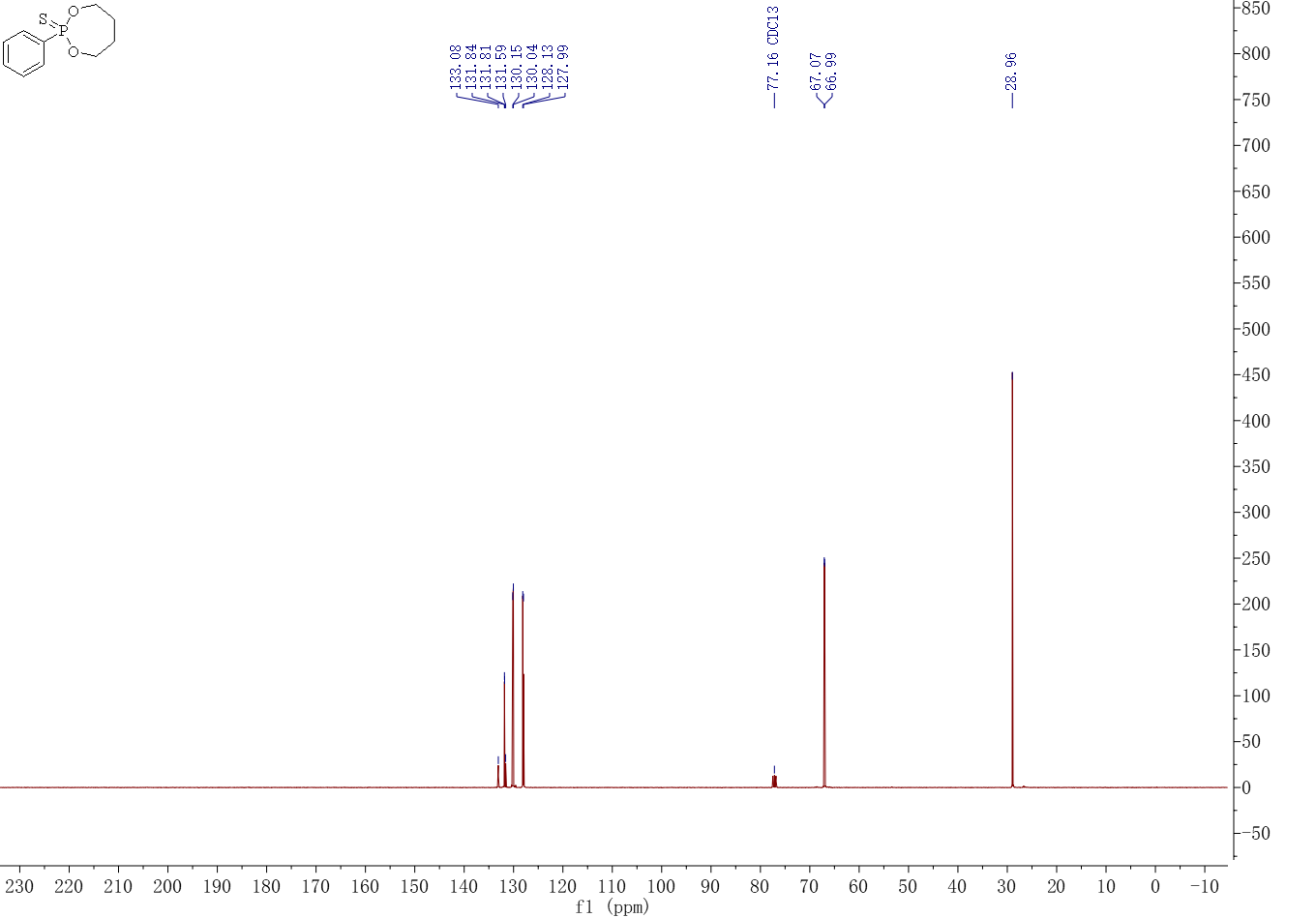


Fig 26. *^13^C NMR of* **S8** (101 MHz, CDCl_3_)


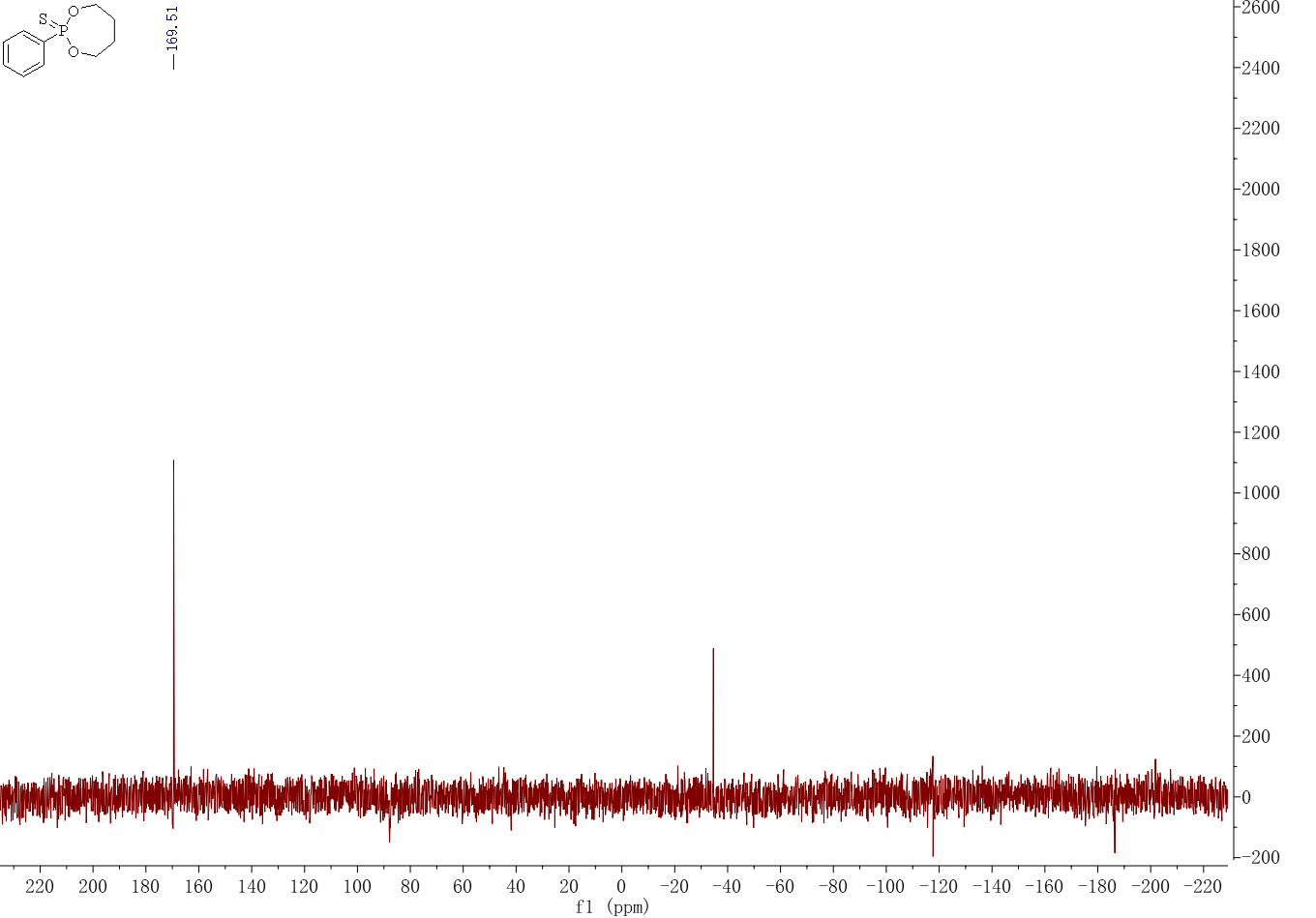


Fig 27. *^31^P NMR of* **S8** (162 MHz, DMSO)


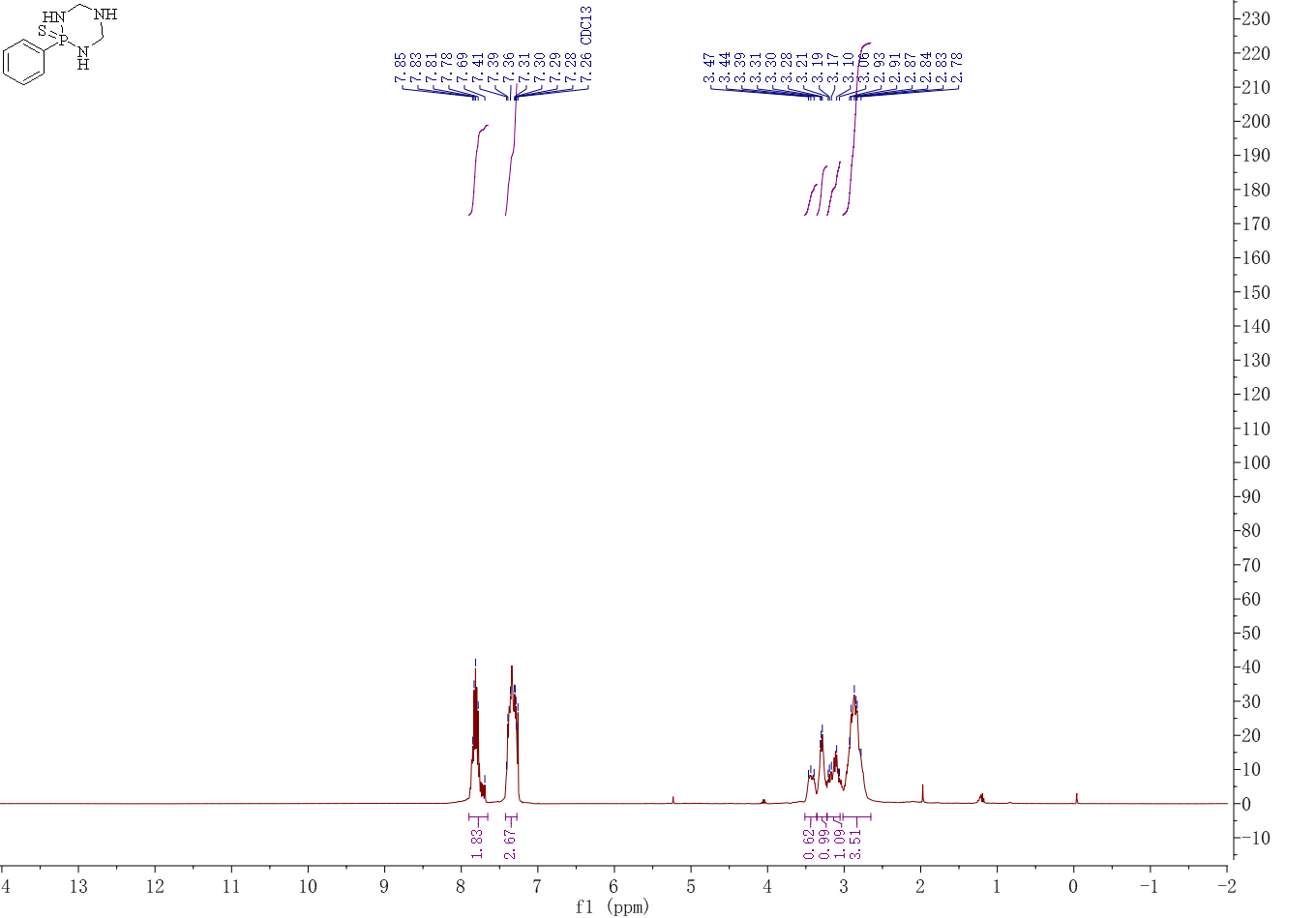


Fig 28. *^1^H NMR of* **S9** (400 MHz, CDCl_3_)


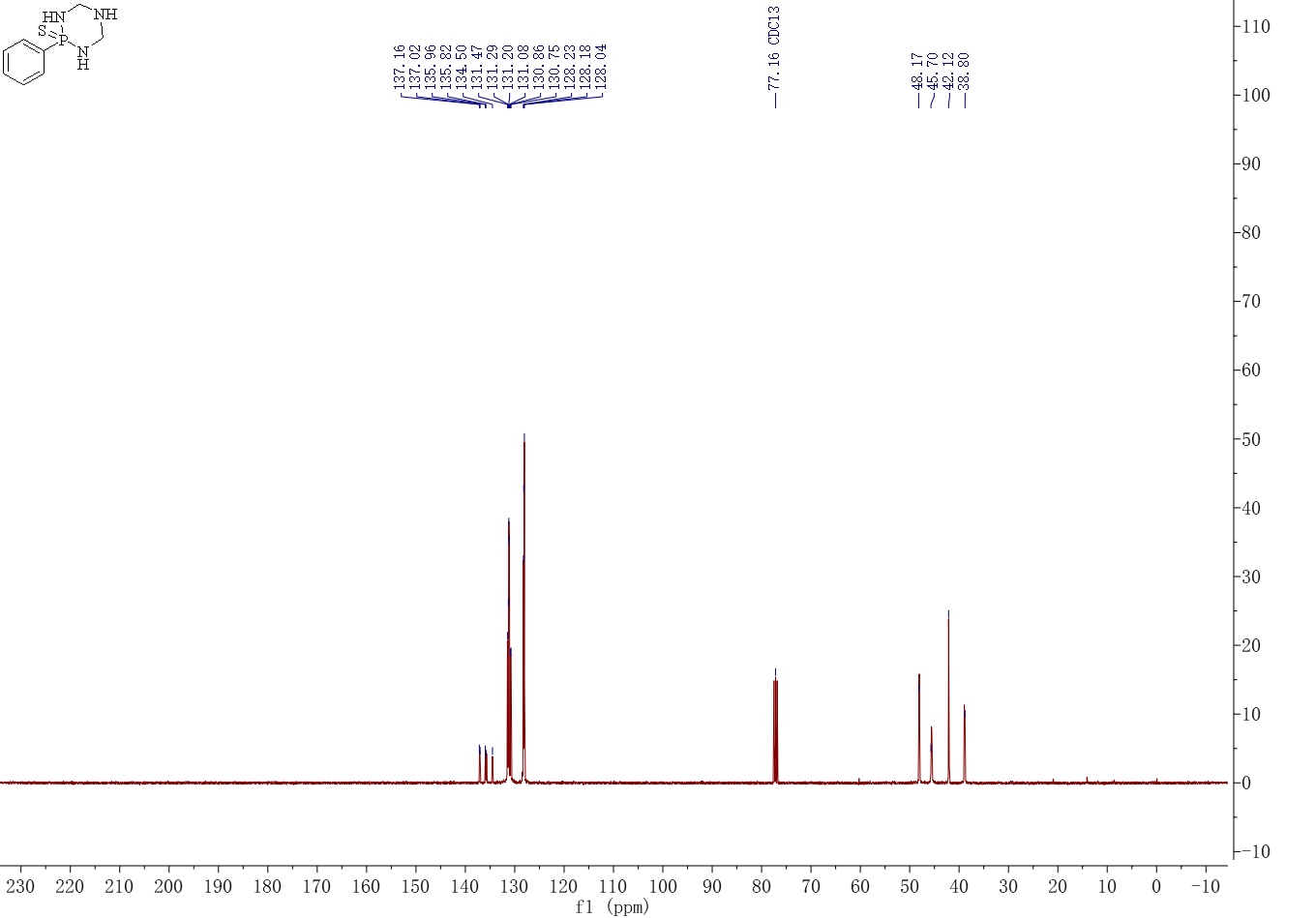


Fig 29. *^13^C NMR of* **S9** (101 MHz, CDCl_3_)


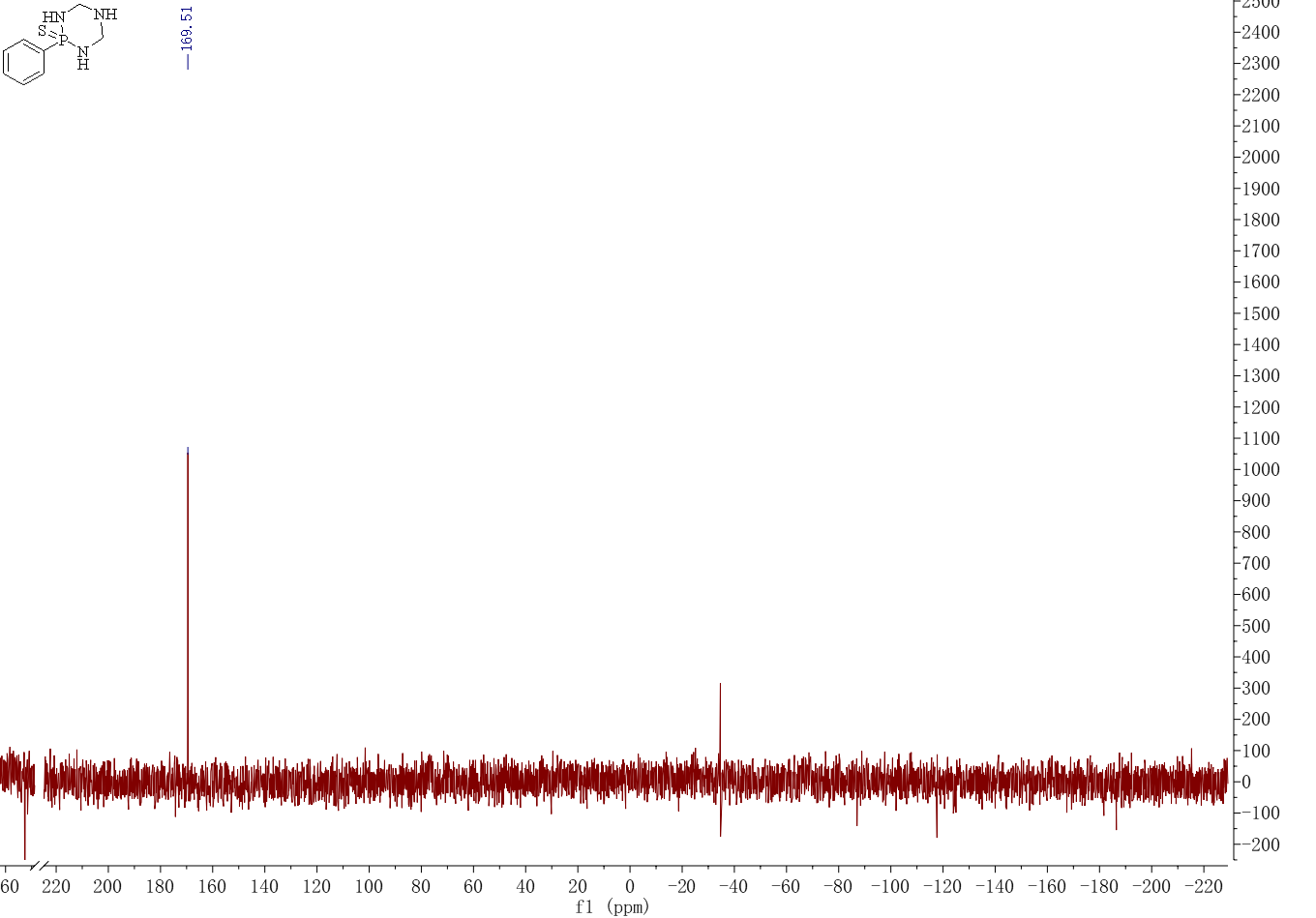


Fig 30. *^31^P NMR of* **S9** (162 MHz, DMSO)


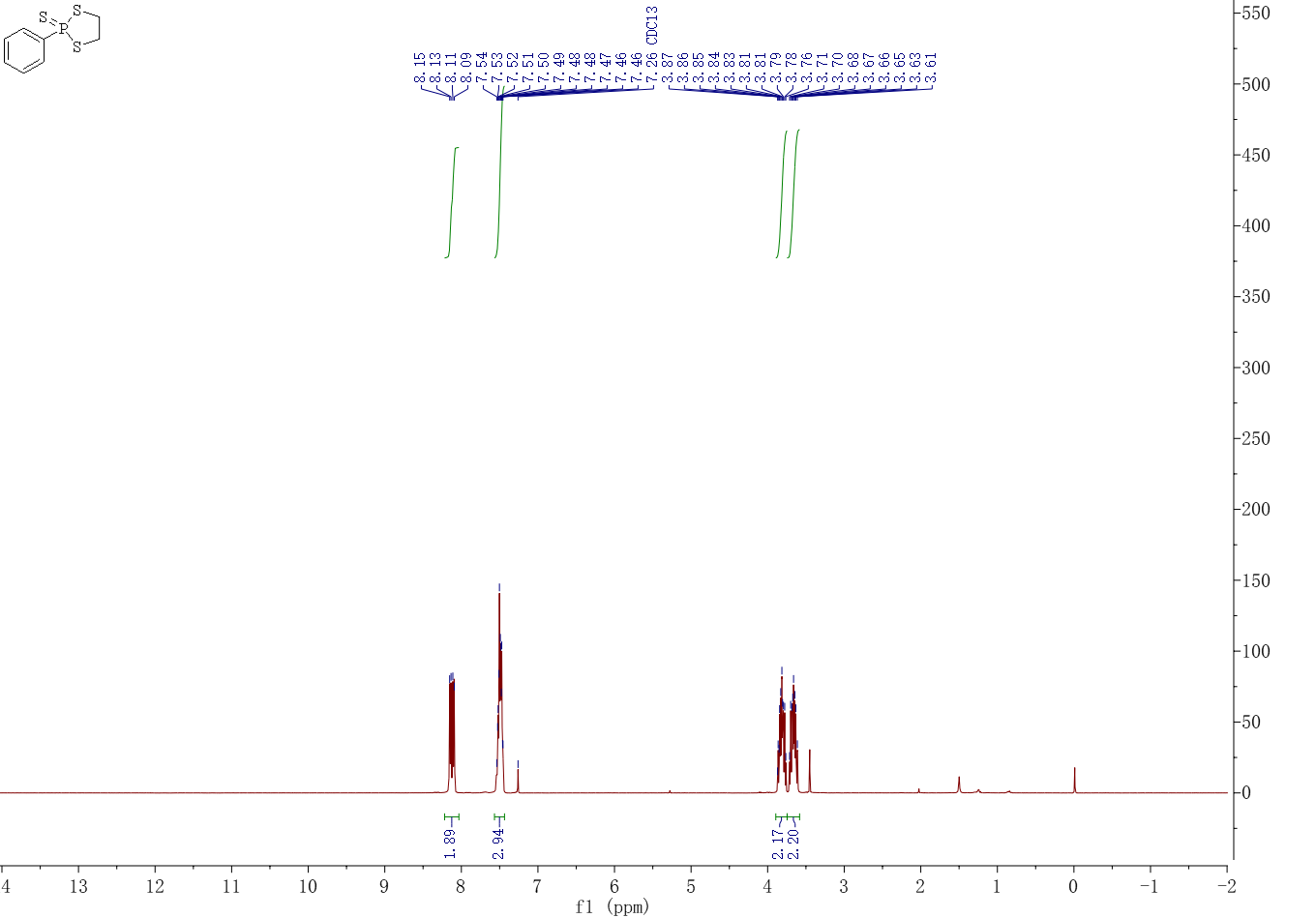


Fig 31. *^1^H NMR of* **S10** (400 MHz, CDCl_3_)


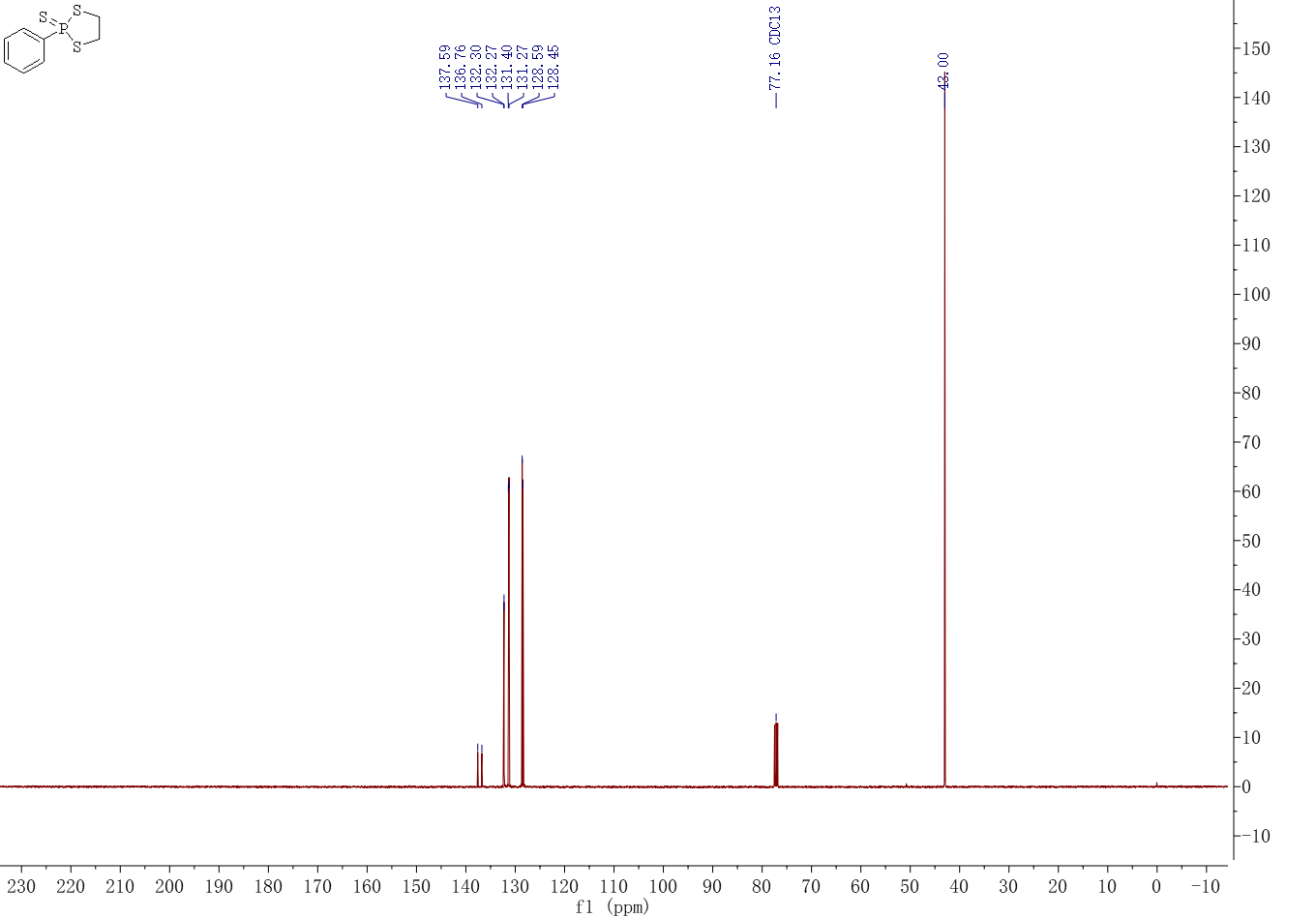


Fig 32. *^13^C NMR of* **S10** (100 MHz, CDCl_3_)


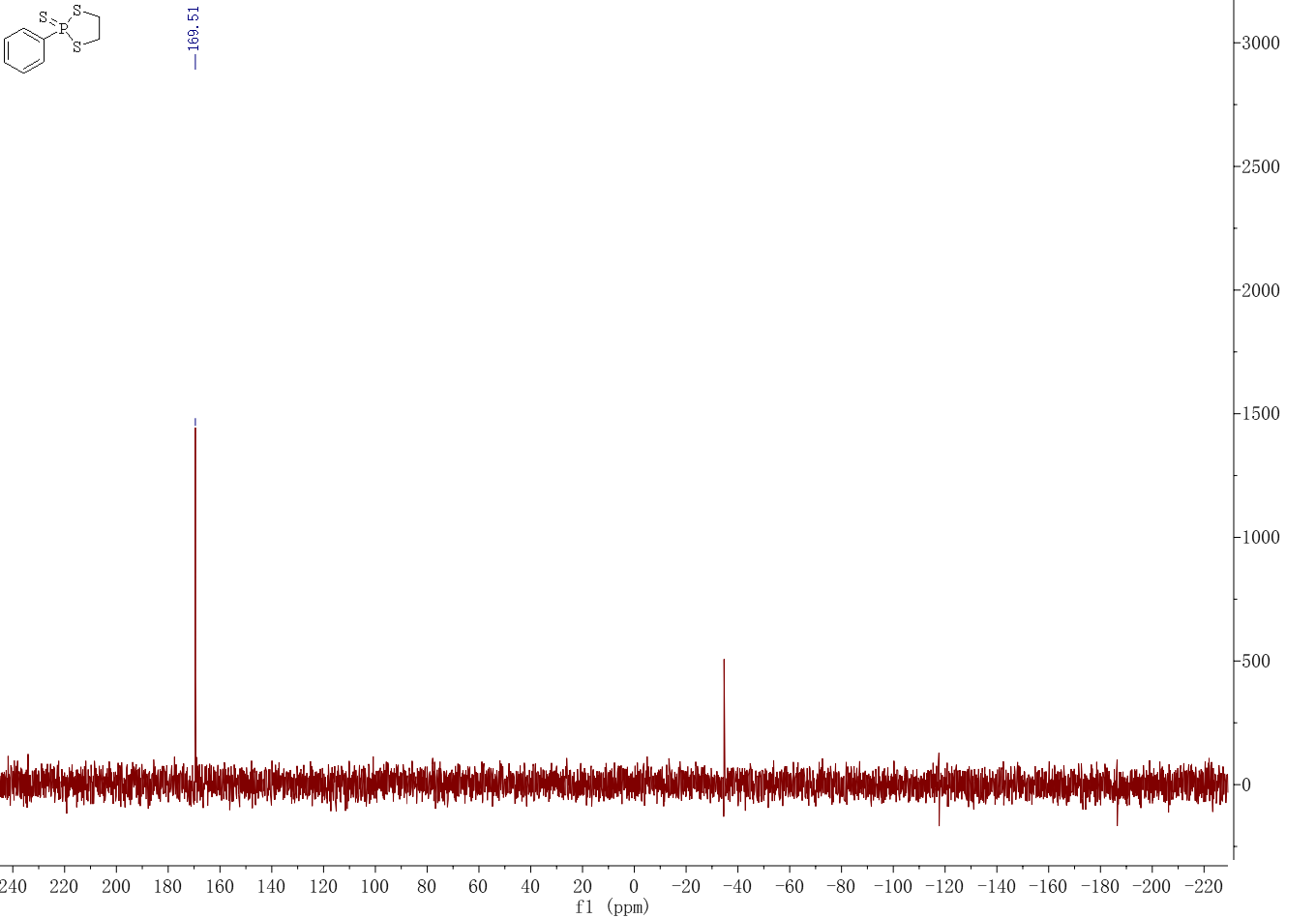


Fig 33. *^31^P NMR of* **S10** (162 MHz, DMSO)


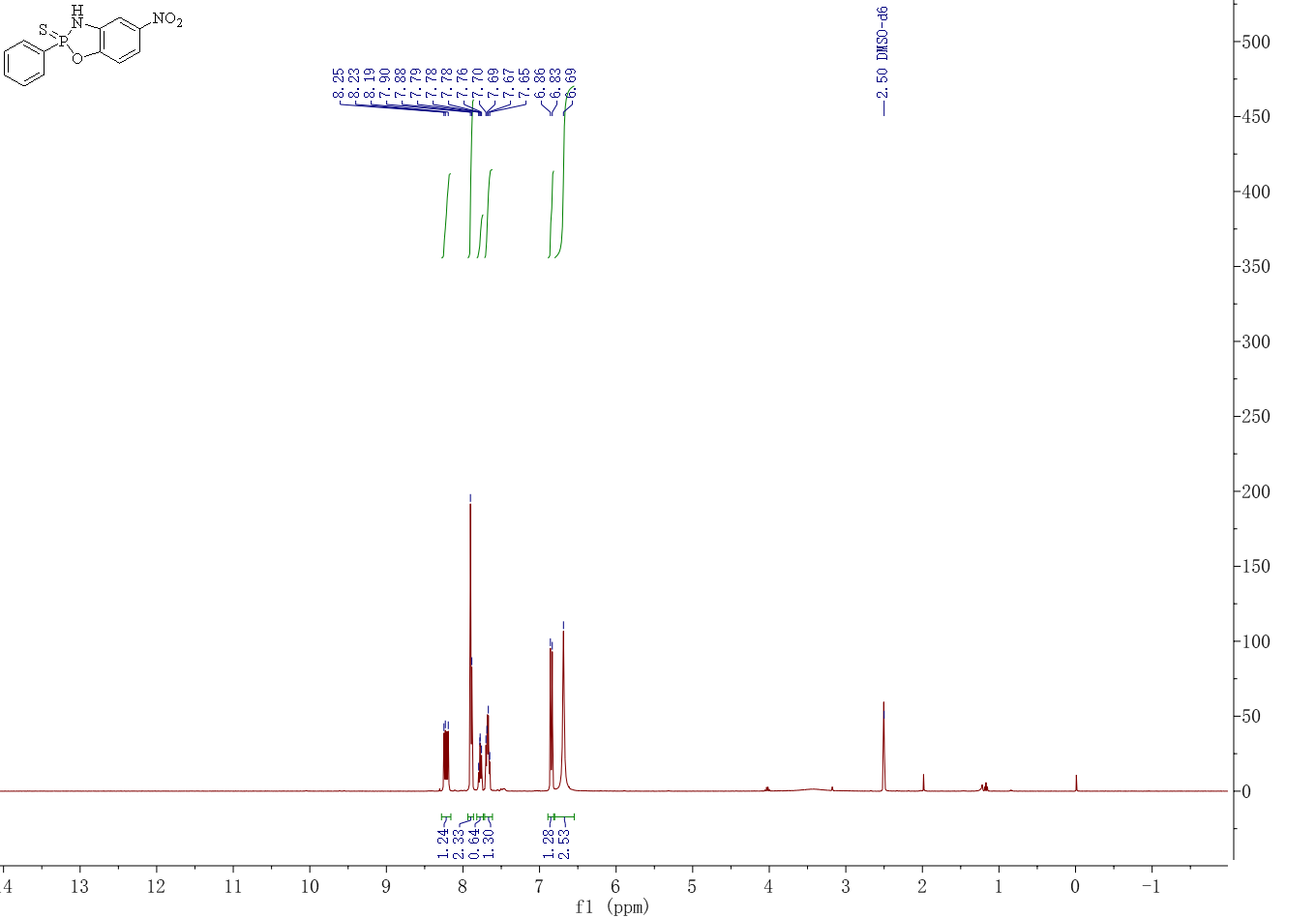


Fig 34. *^1^H NMR of* **S11** (400 MHz, DMSO)


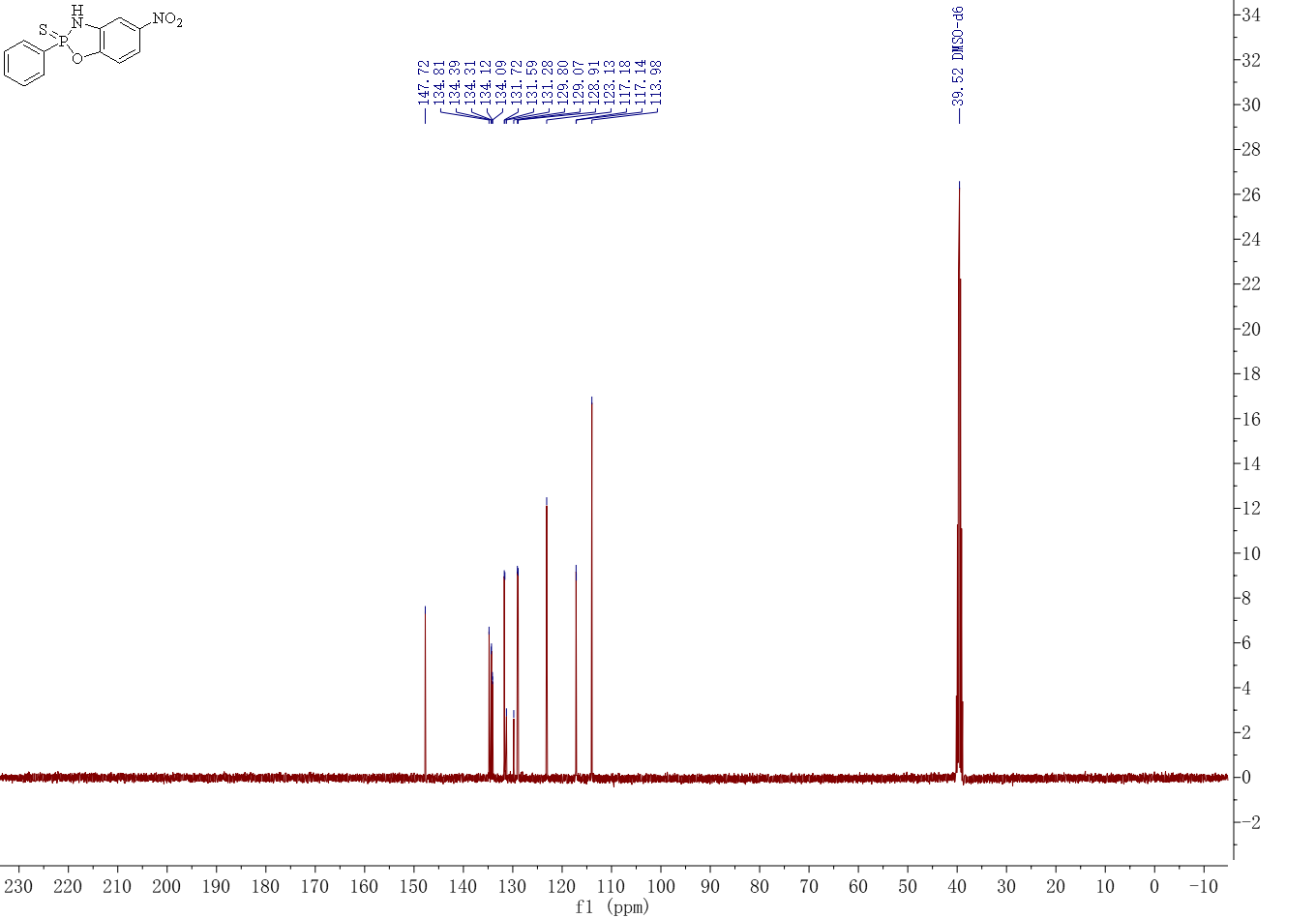


Fig 35. *^13^C NMR of* **S11** (101 MHz, DMSO)


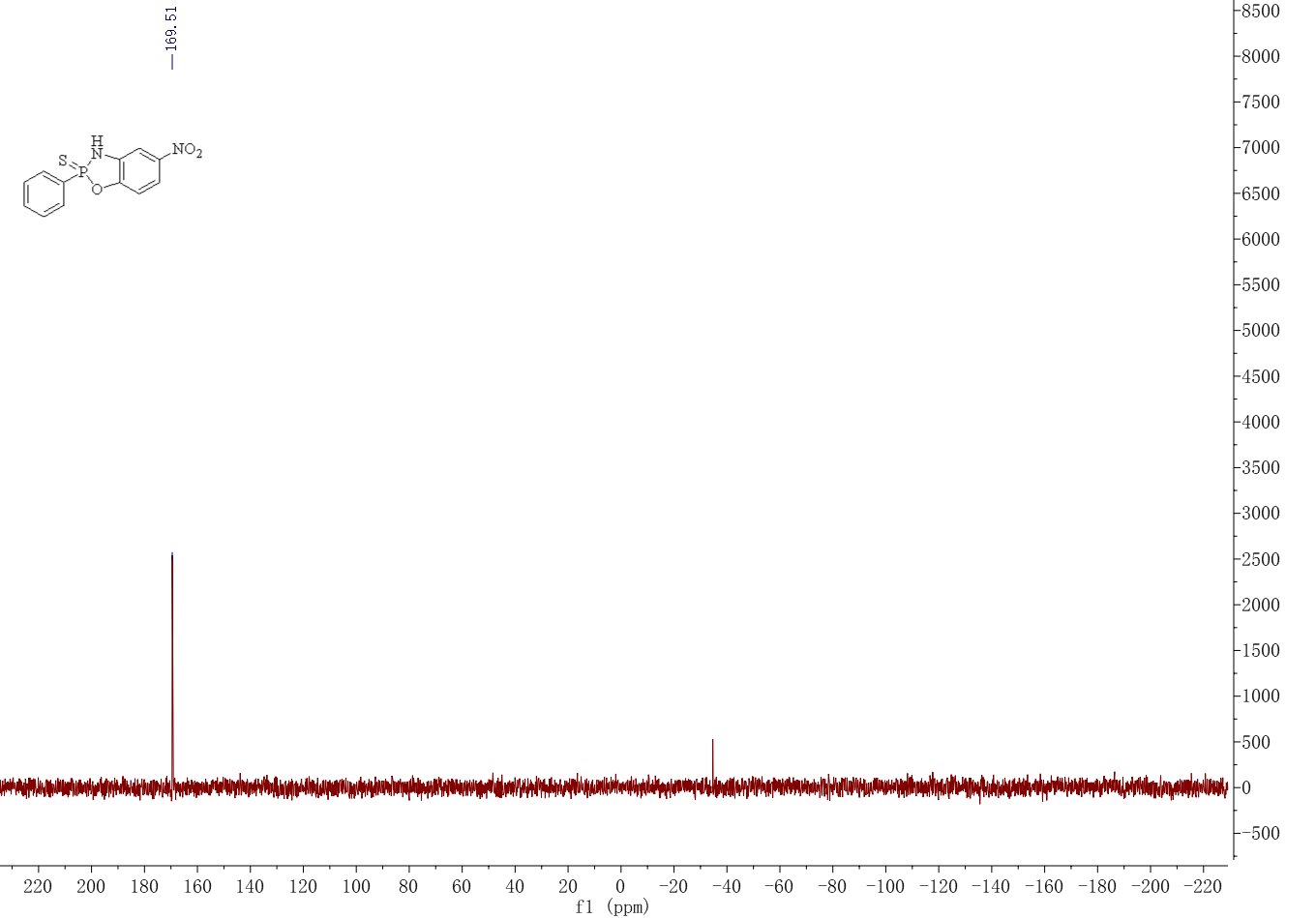


Fig 36. *^31^P NMR of* **S11** (162 MHz, DMSO)


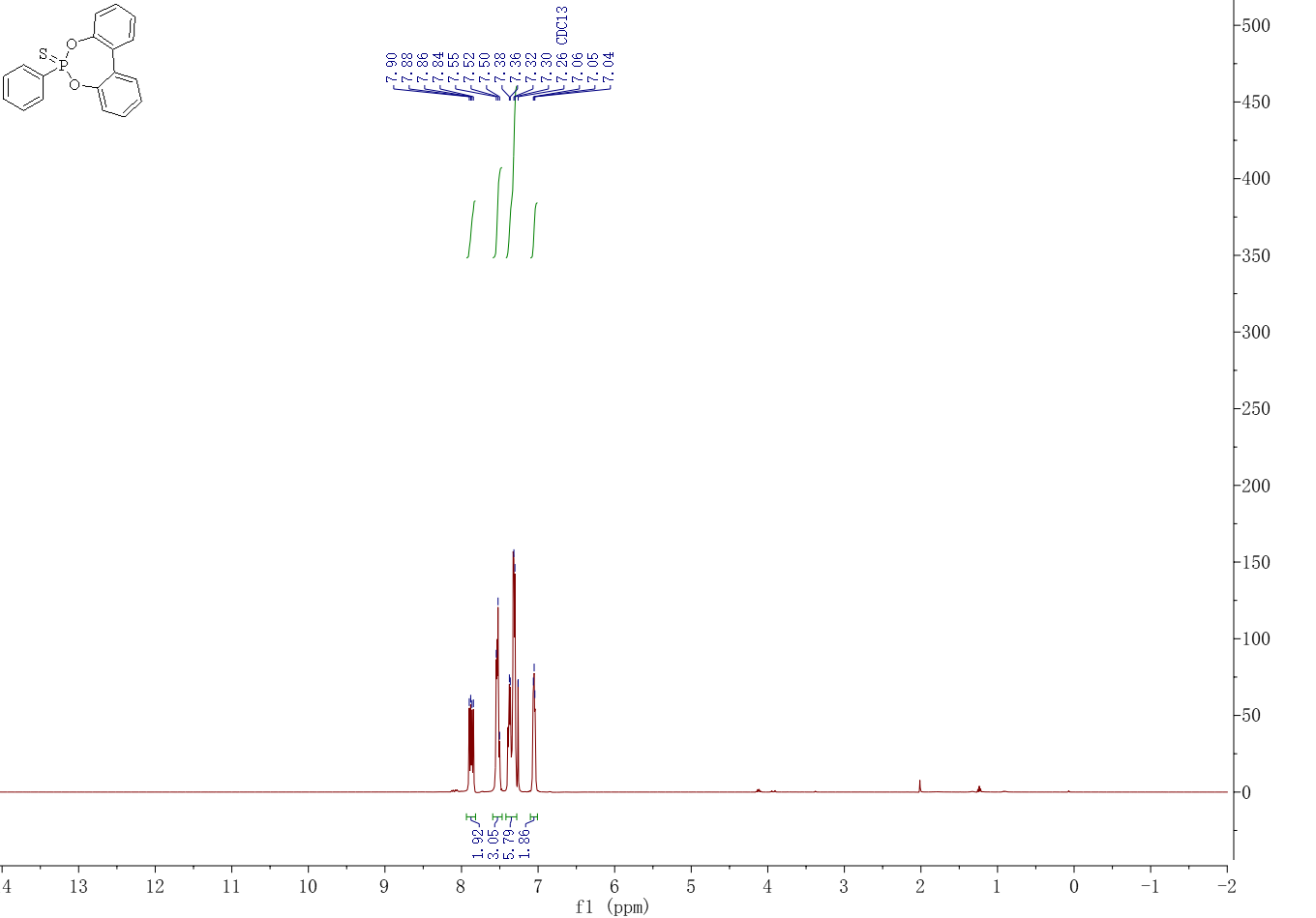


Fig 37. *^1^H NMR of* **S12** (400 MHz, CDCl_3_)


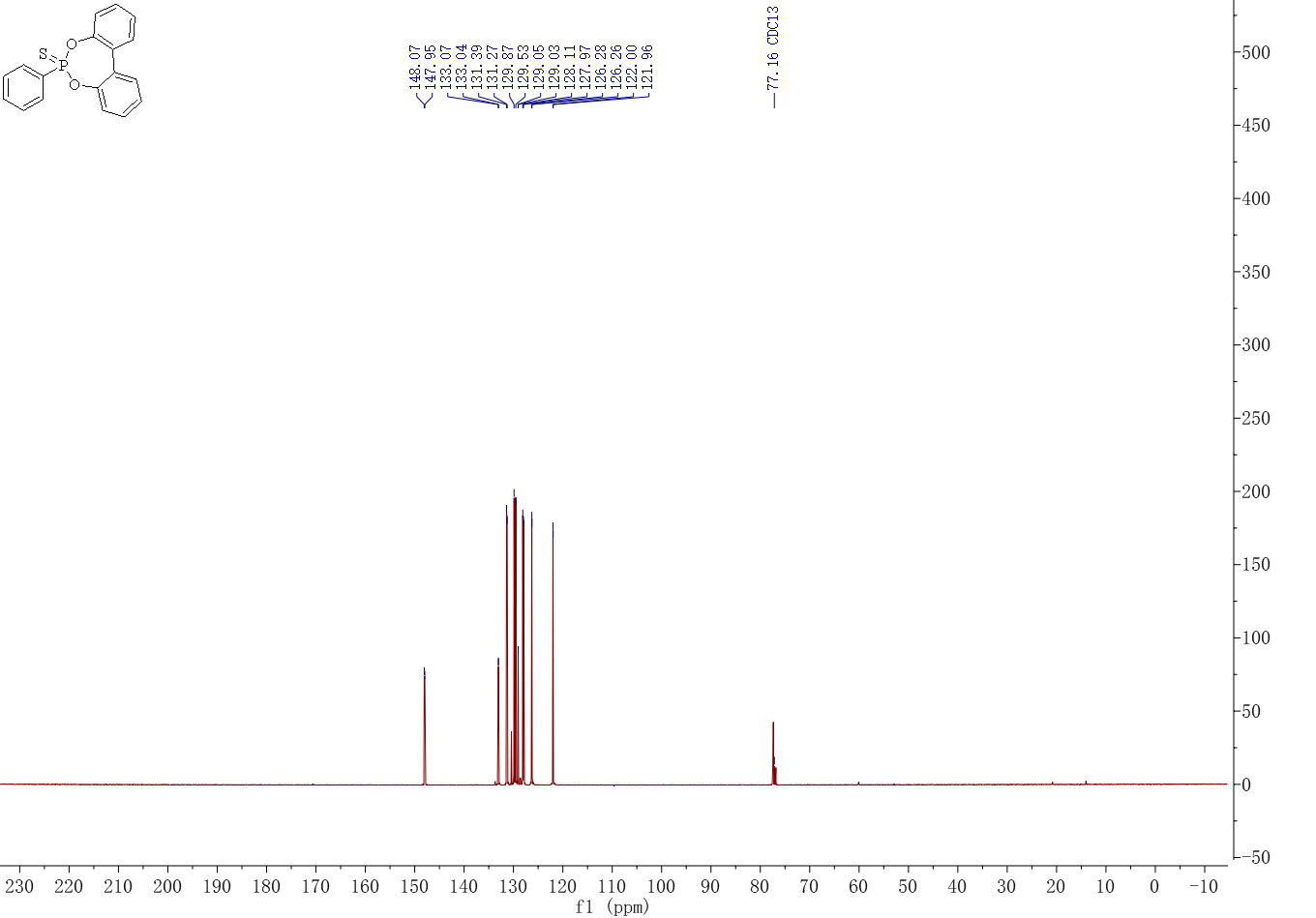


Fig 38. *^13^C NMR of* **S12** (101 MHz, CDCl_3_)


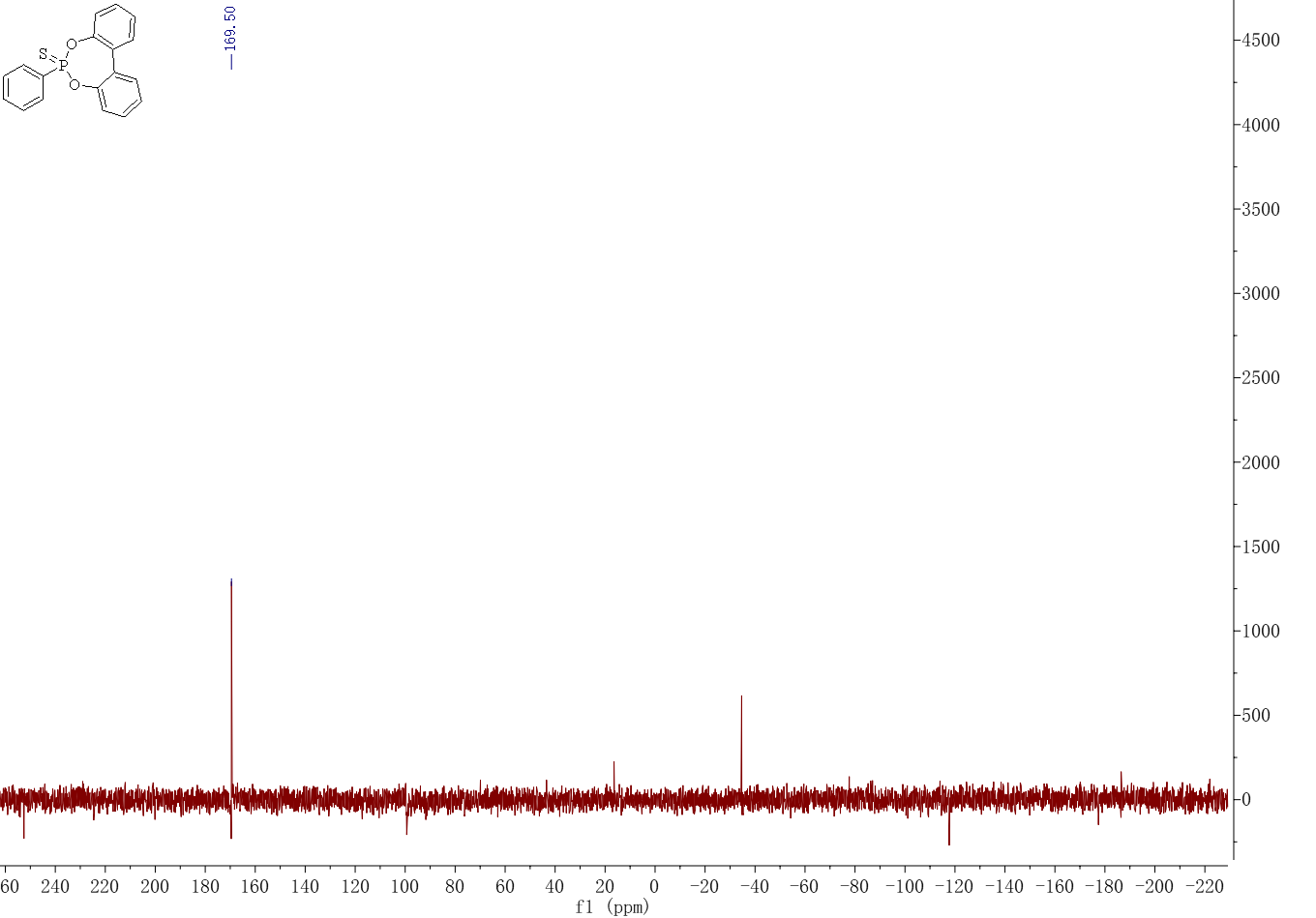


Fig 39. *^31^P NMR of* **S12** (162 MHz, DMSO)


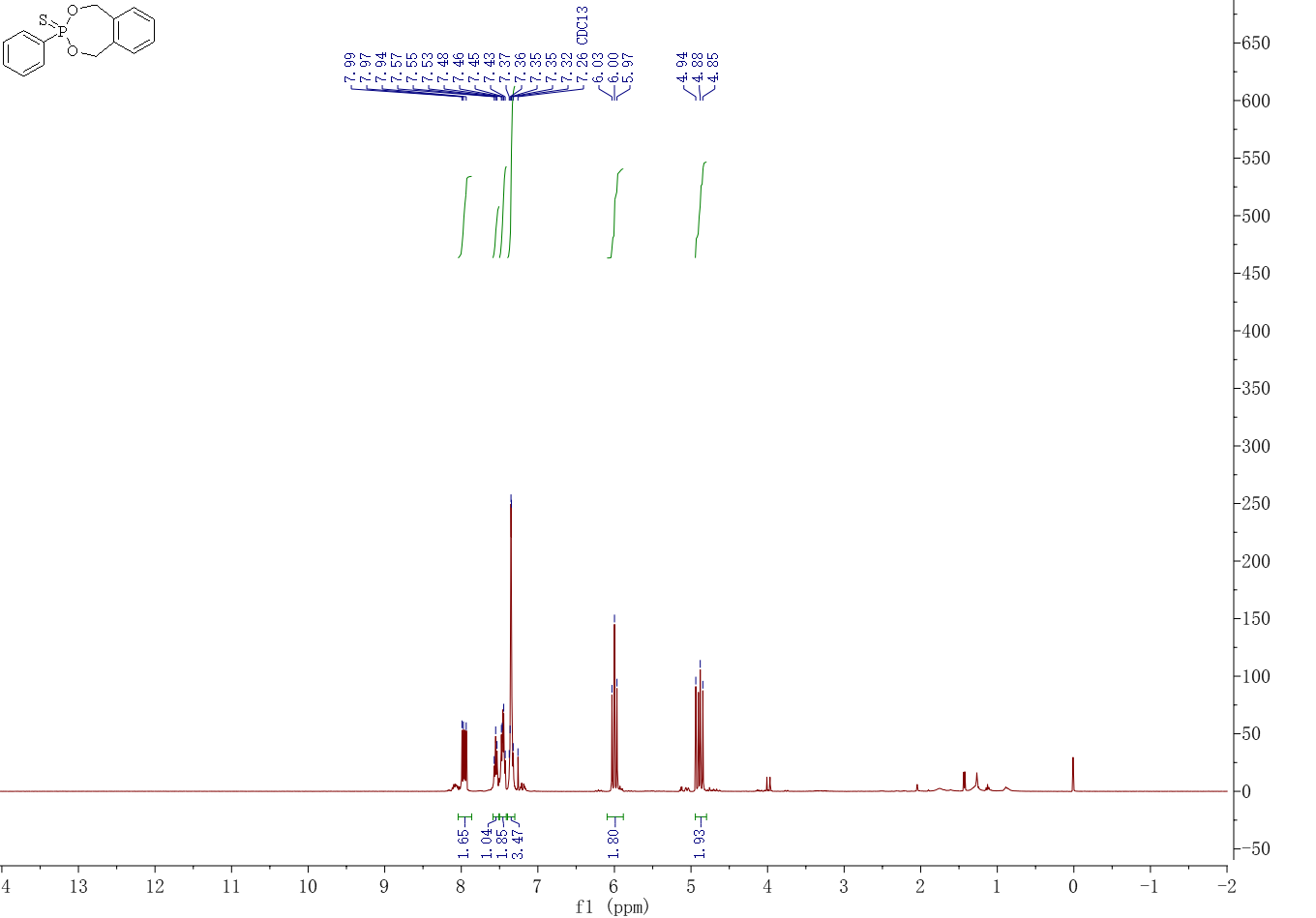


Fig 40. *^1^H NMR of* **S13** (400 MHz, CDCl_3_)


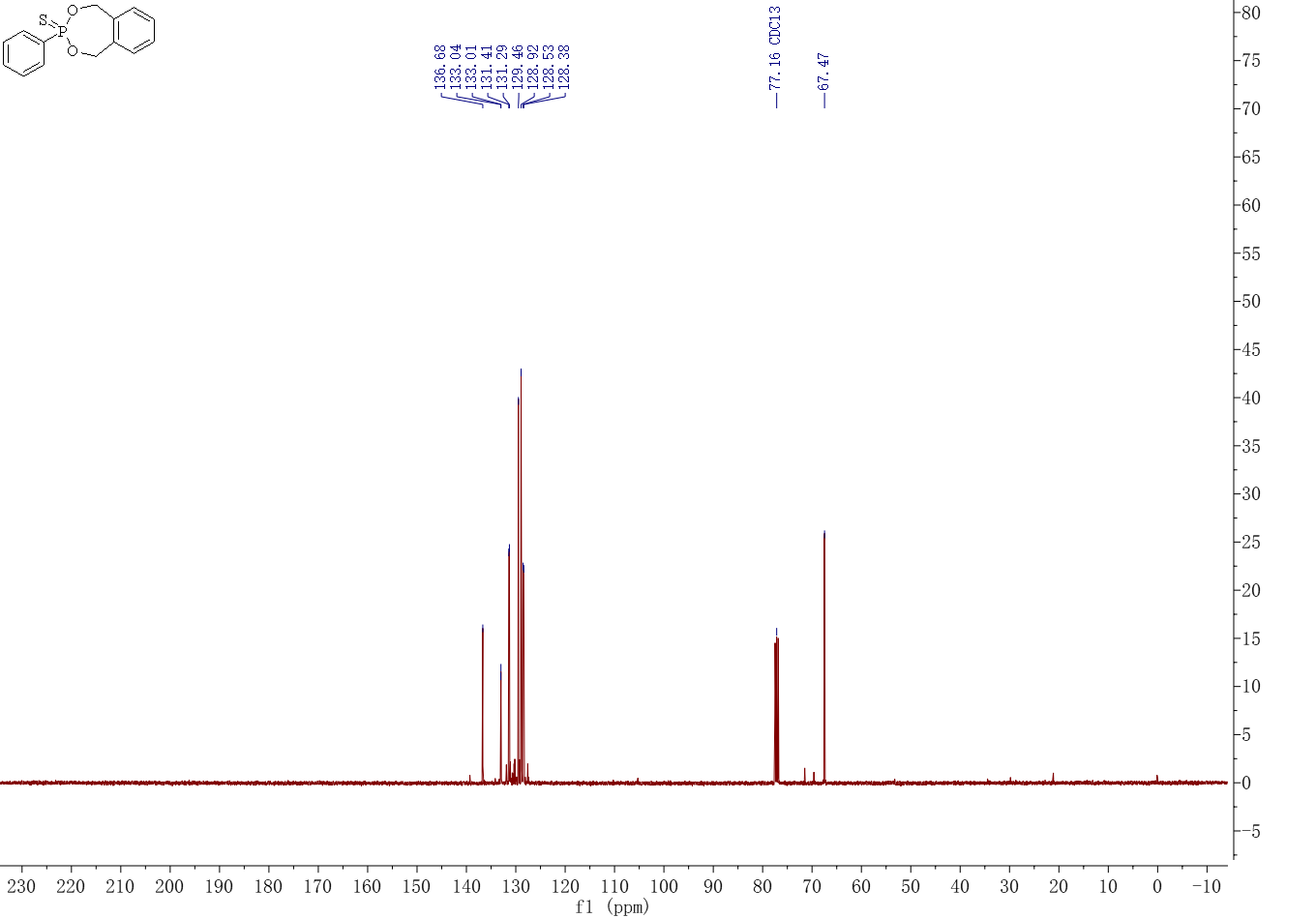


Fig 41. *^13^C NMR of* **S13** (100 MHz, CDCl_3_)


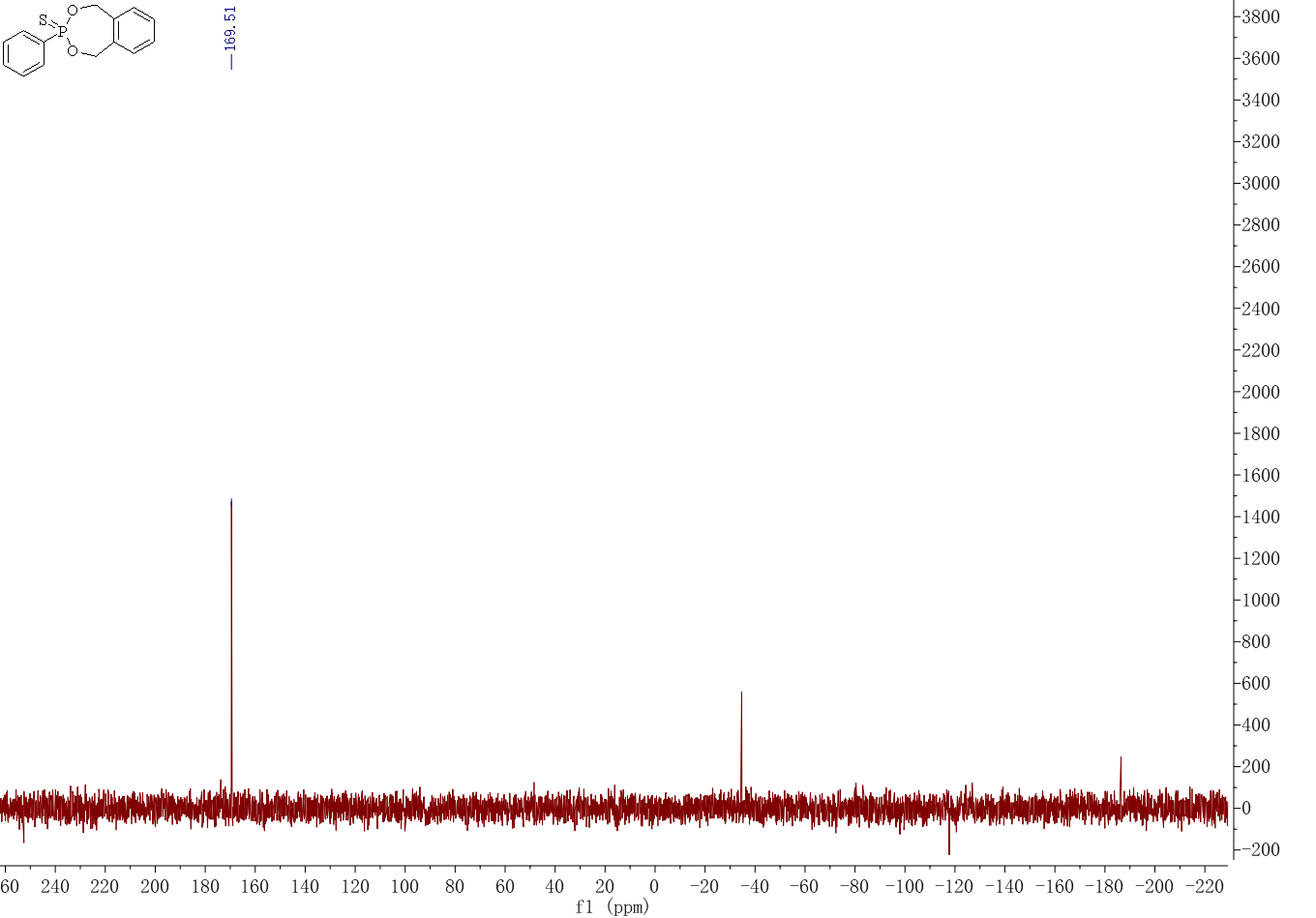


Fig 42. *^31^P NMR of* **S13** (162 MHz, DMSO)


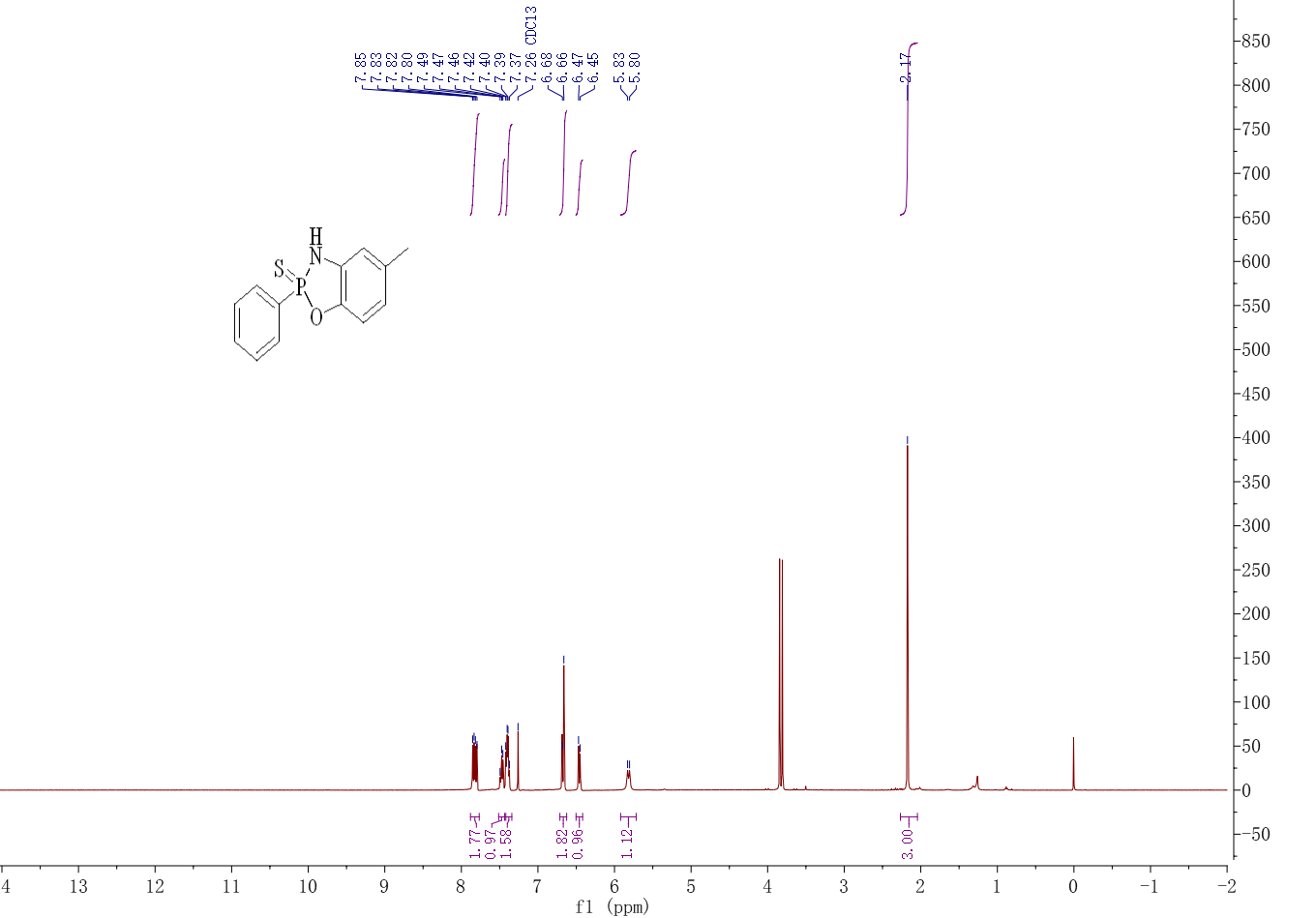


Fig 43. *^1^H NMR of* **S14** (400 MHz, CDCl_3_)


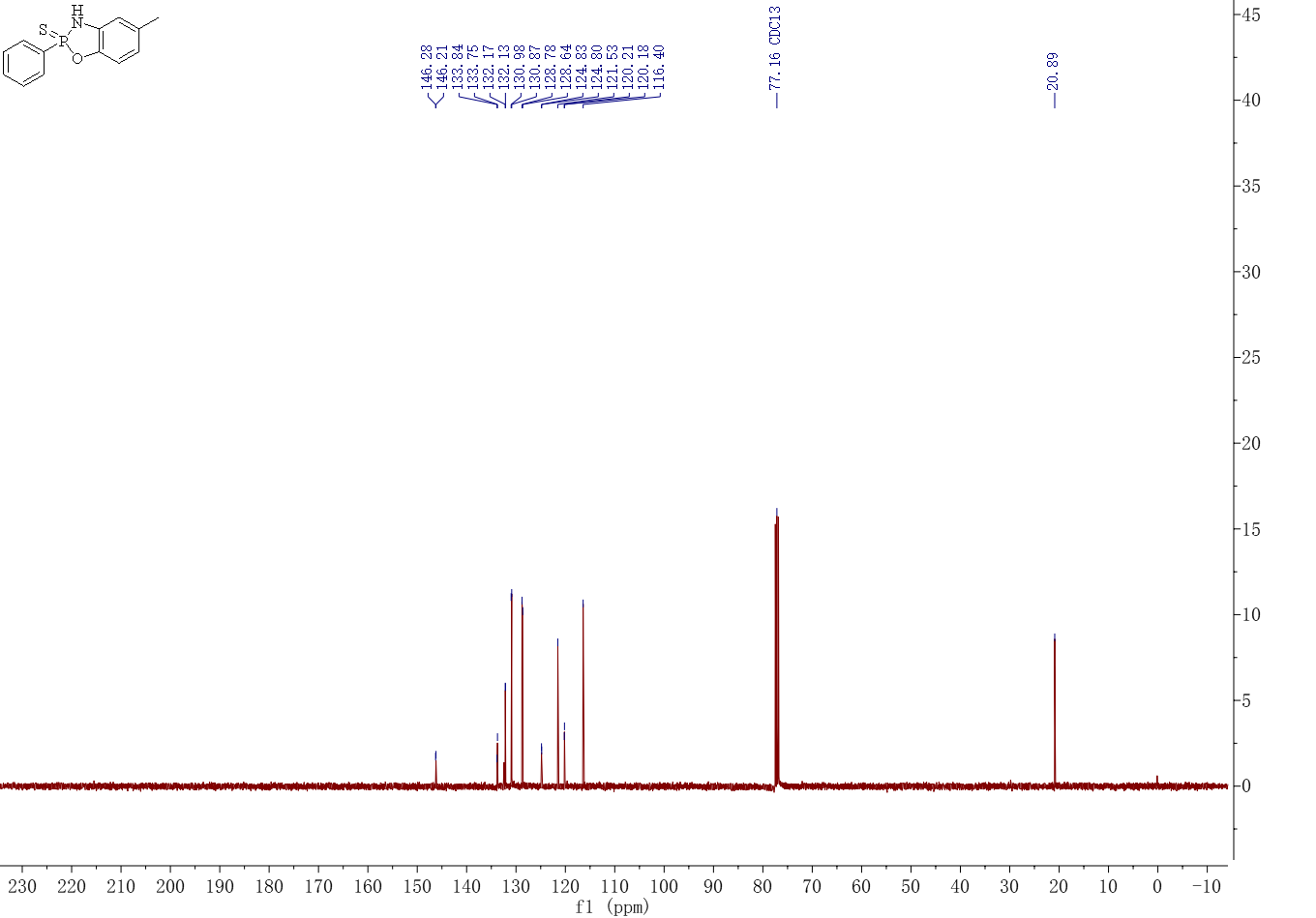


Fig 44. *^13^C NMR of* **S14** (100 MHz, CDCl_3_)


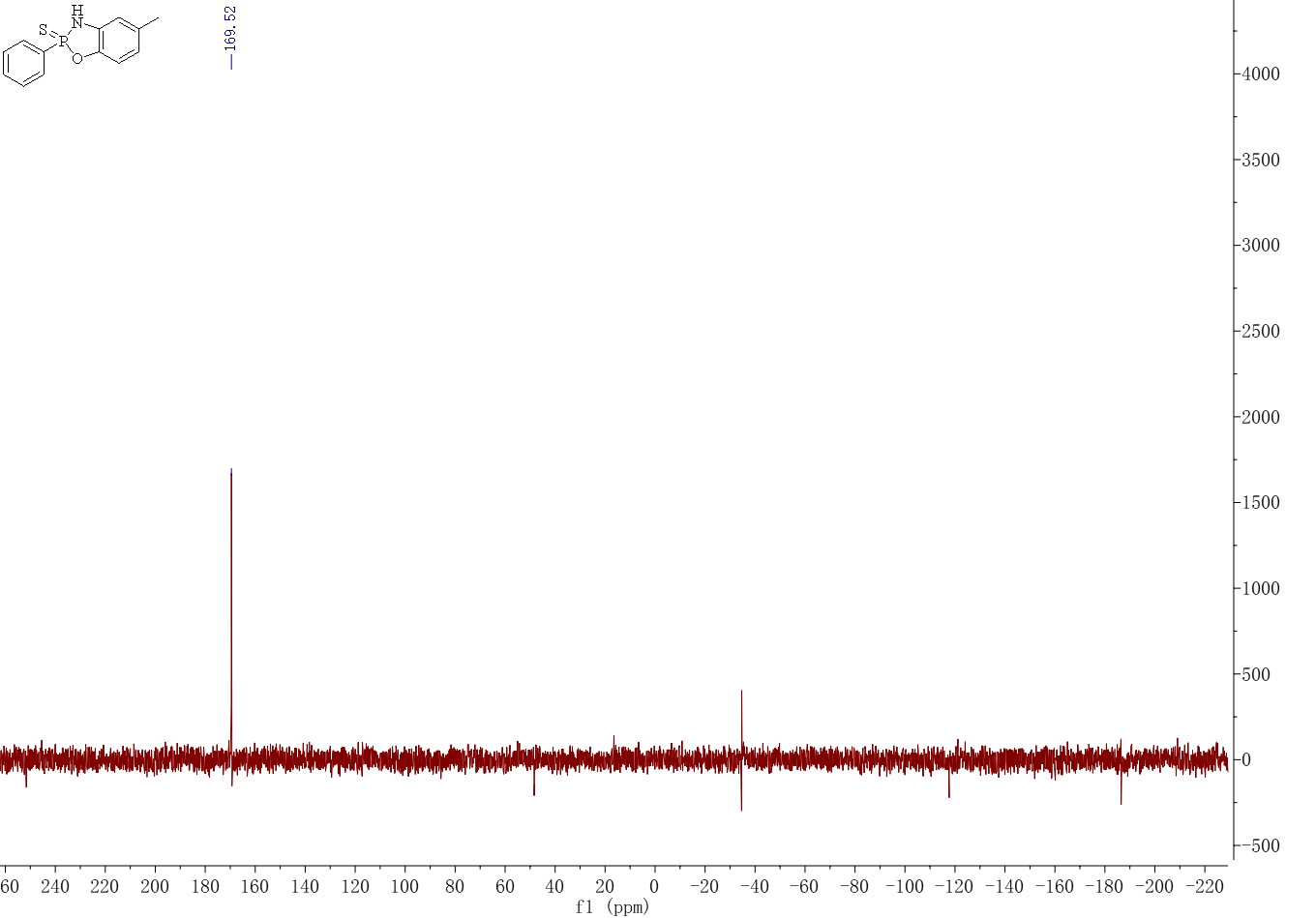


Fig 45. *^31^P NMR of* **S14** (162 MHz, DMSO)


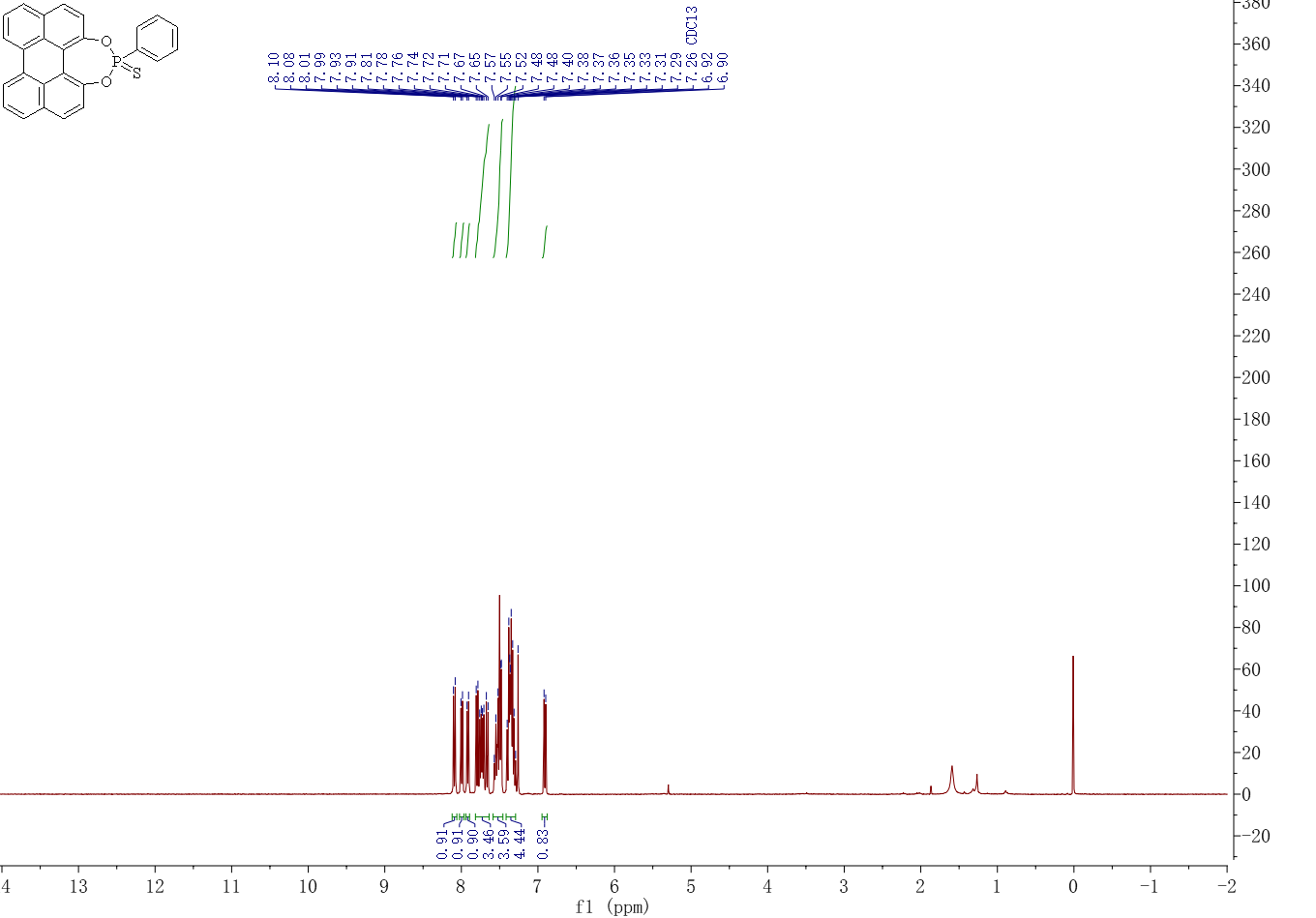


Fig 46. *^1^H NMR of* **S15** (400 MHz, CDCl_3_)


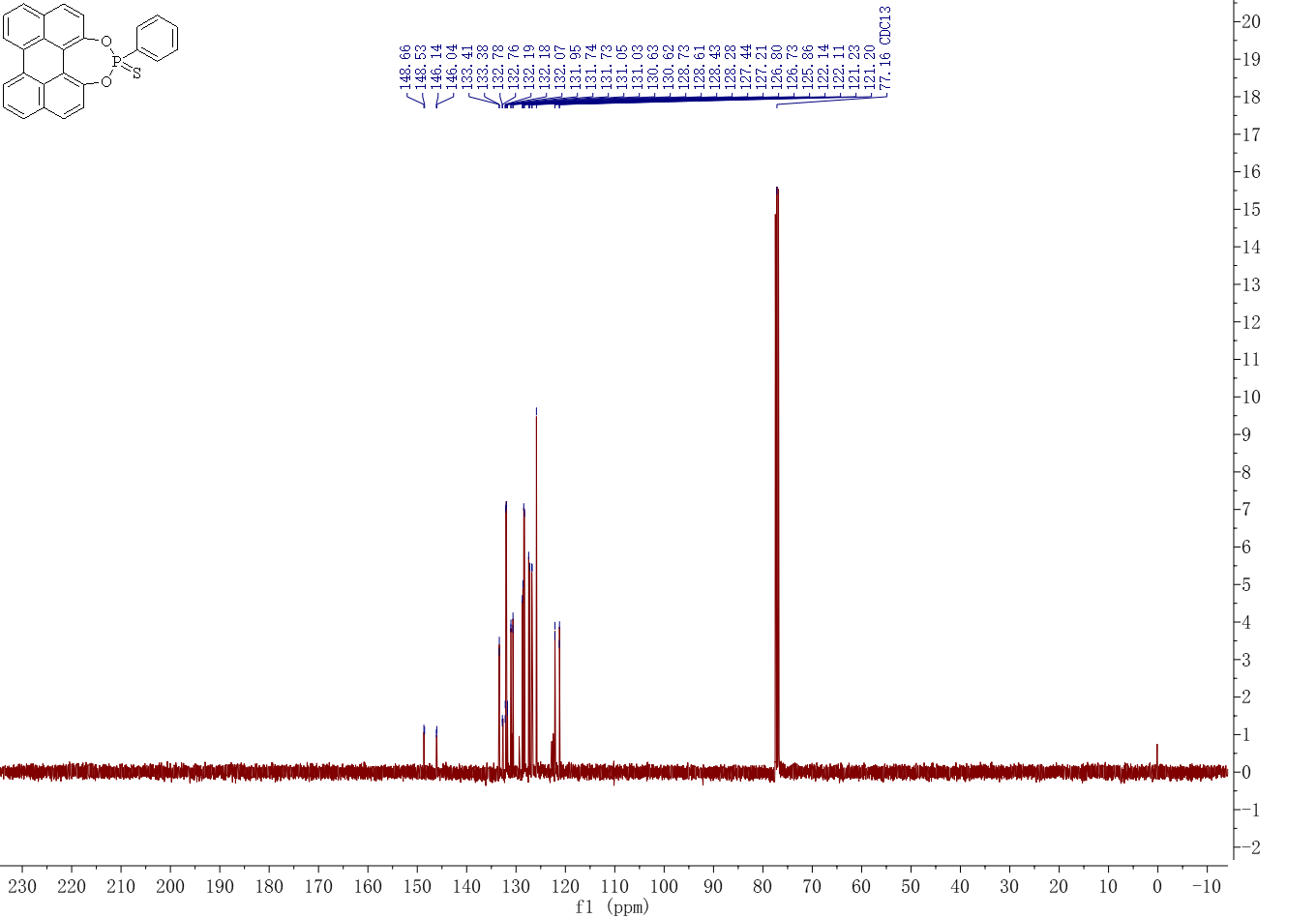


Fig 47. *^13^C NMR of* **S15** (100 MHz, CDCl_3_)


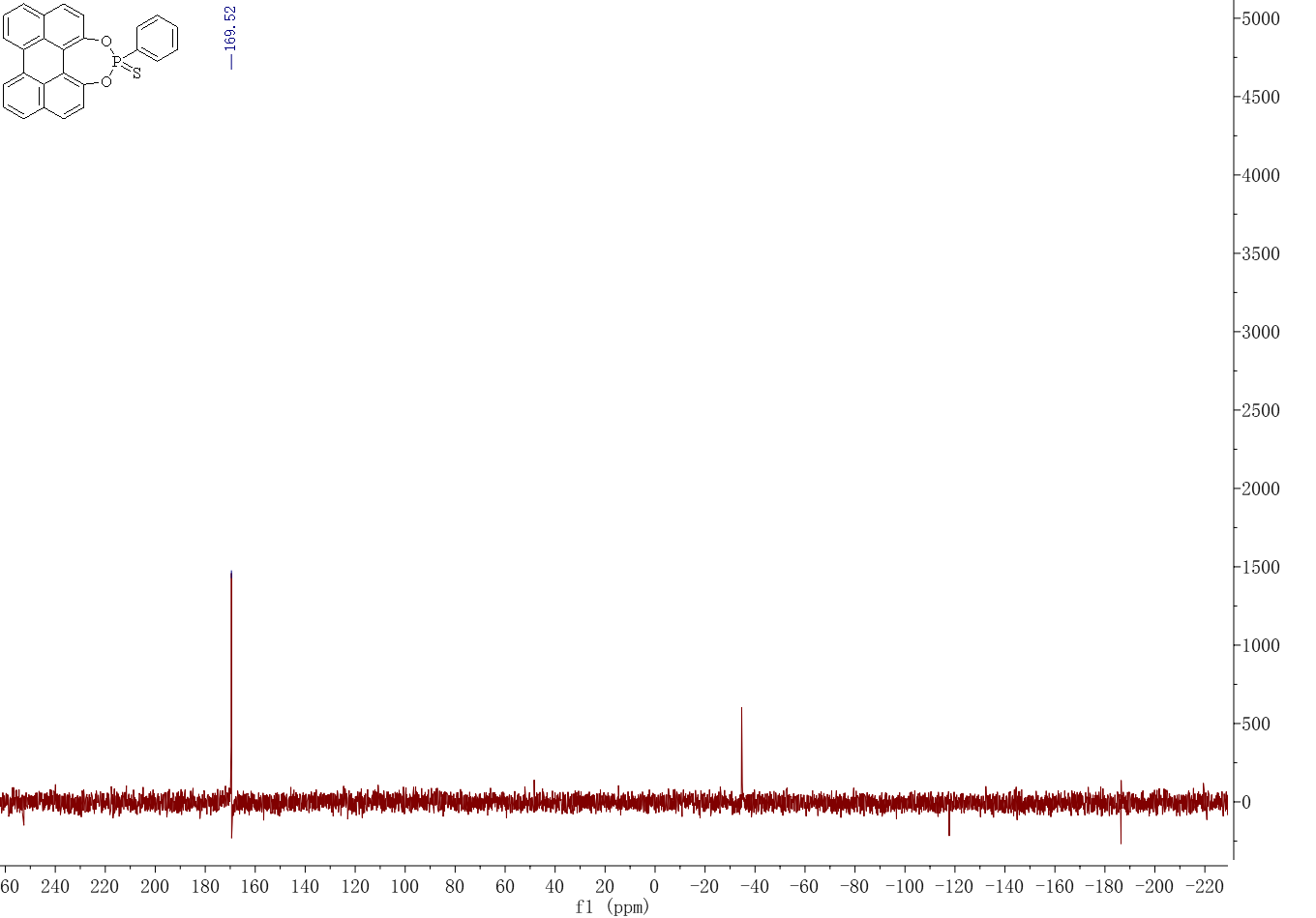


Fig 48. *^31^P NMR of* **S15** (162 MHz, DMSO)


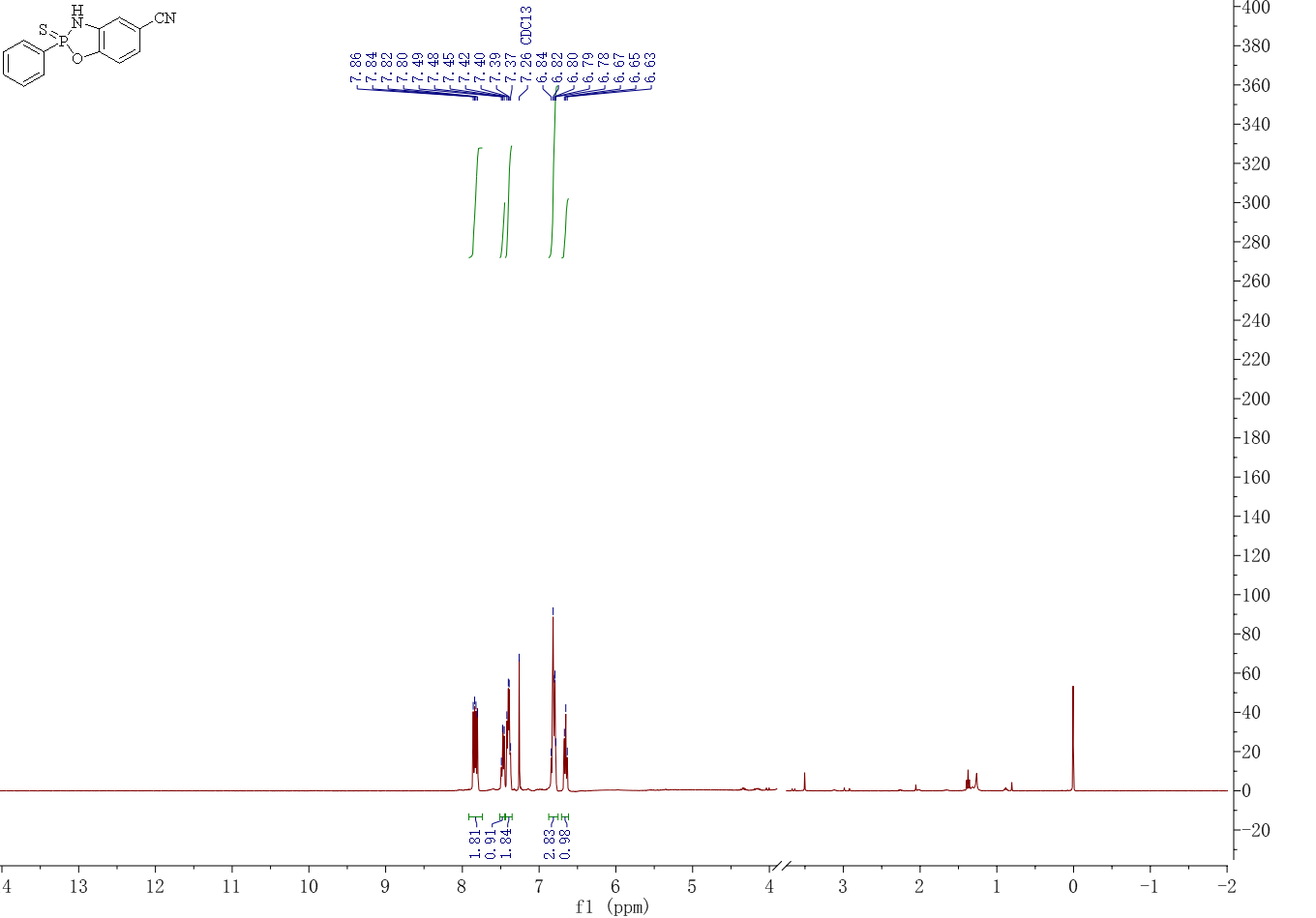


Fig 49. *^1^H NMR of* **S16** (400 MHz, CDCl_3_)


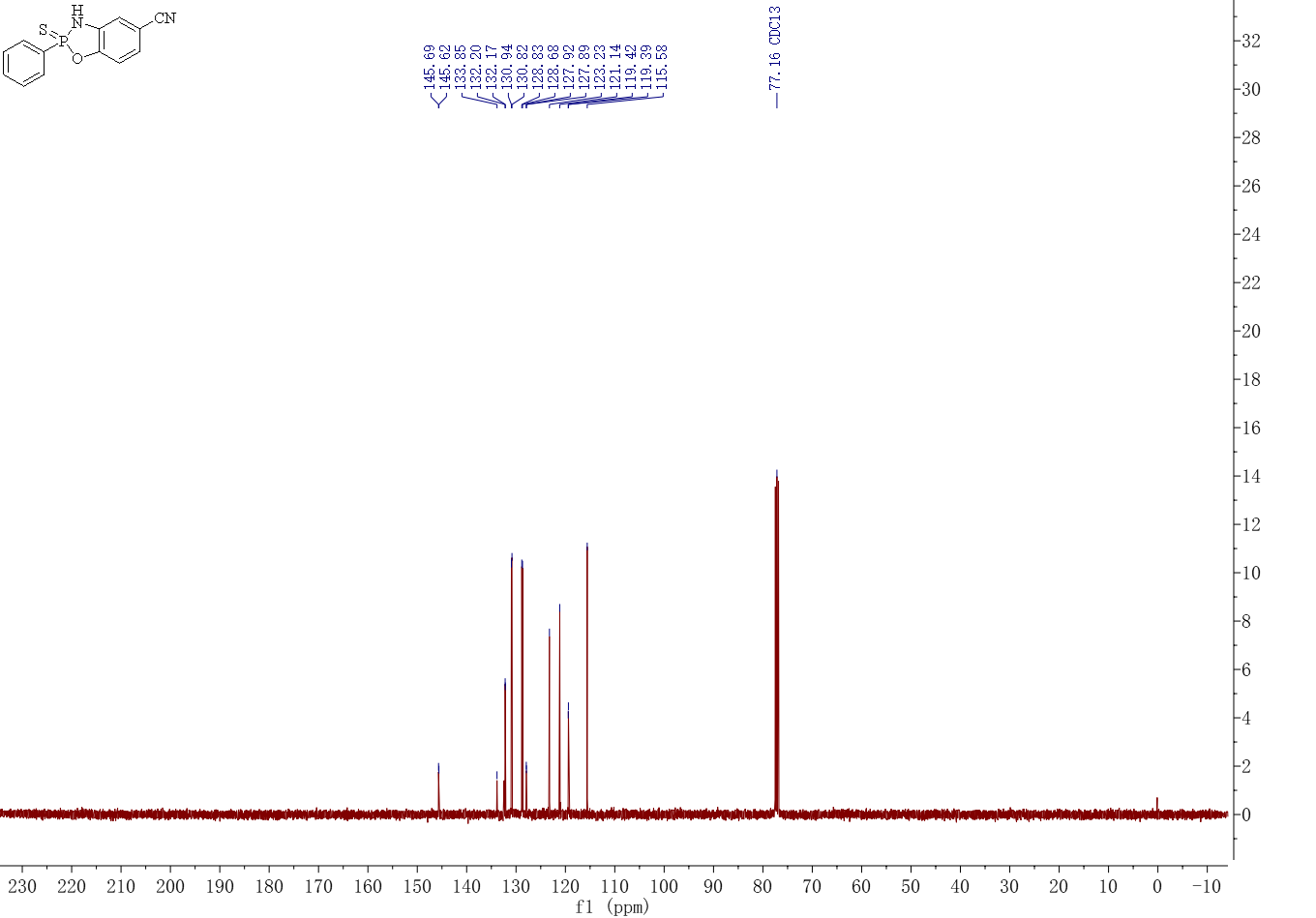


Fig 50. *^13^C NMR of* **S16** (101 MHz, CDCl_3_)


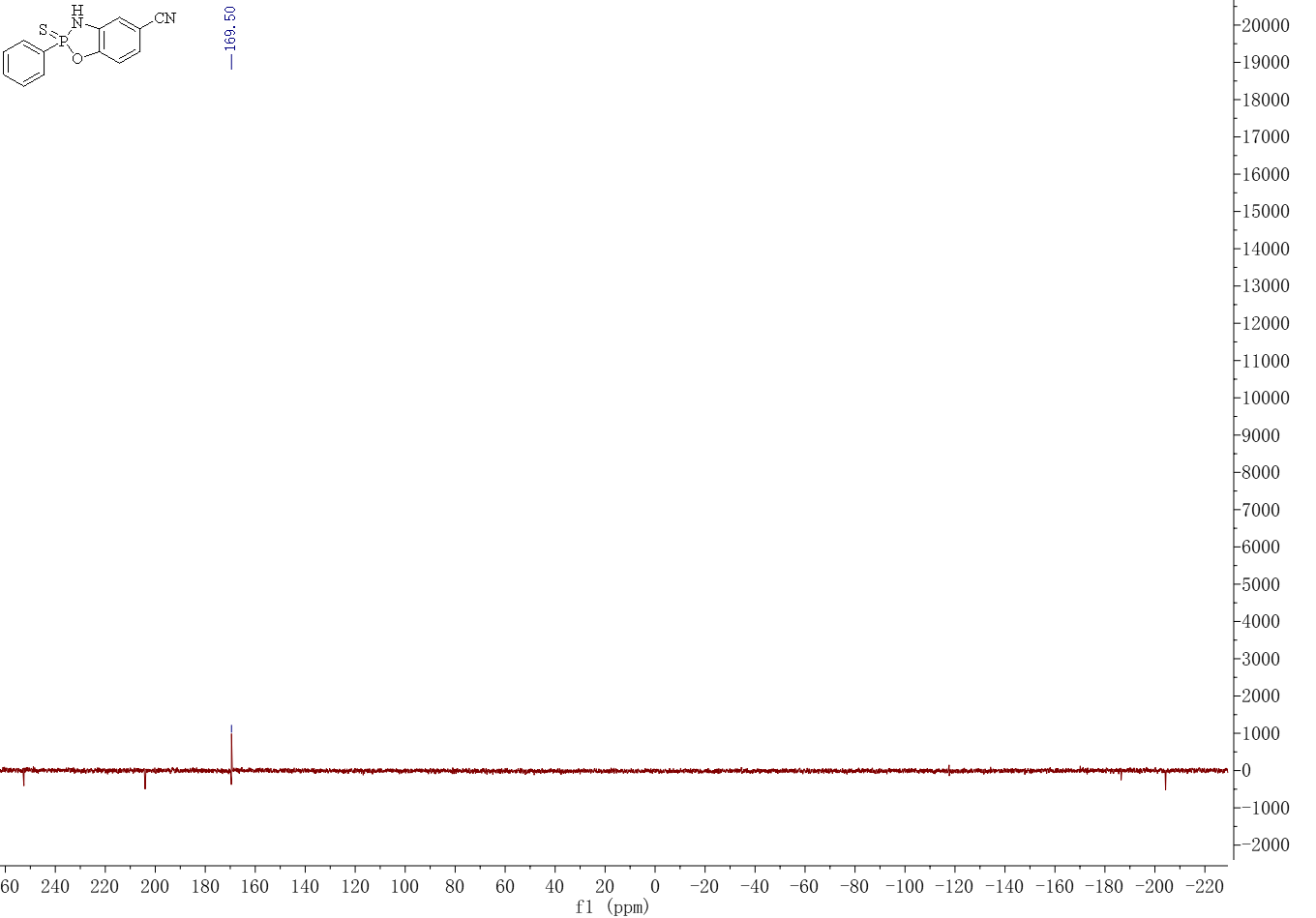


Fig 51. *^31^P NMR of* **S16** (162 MHz, DMSO)


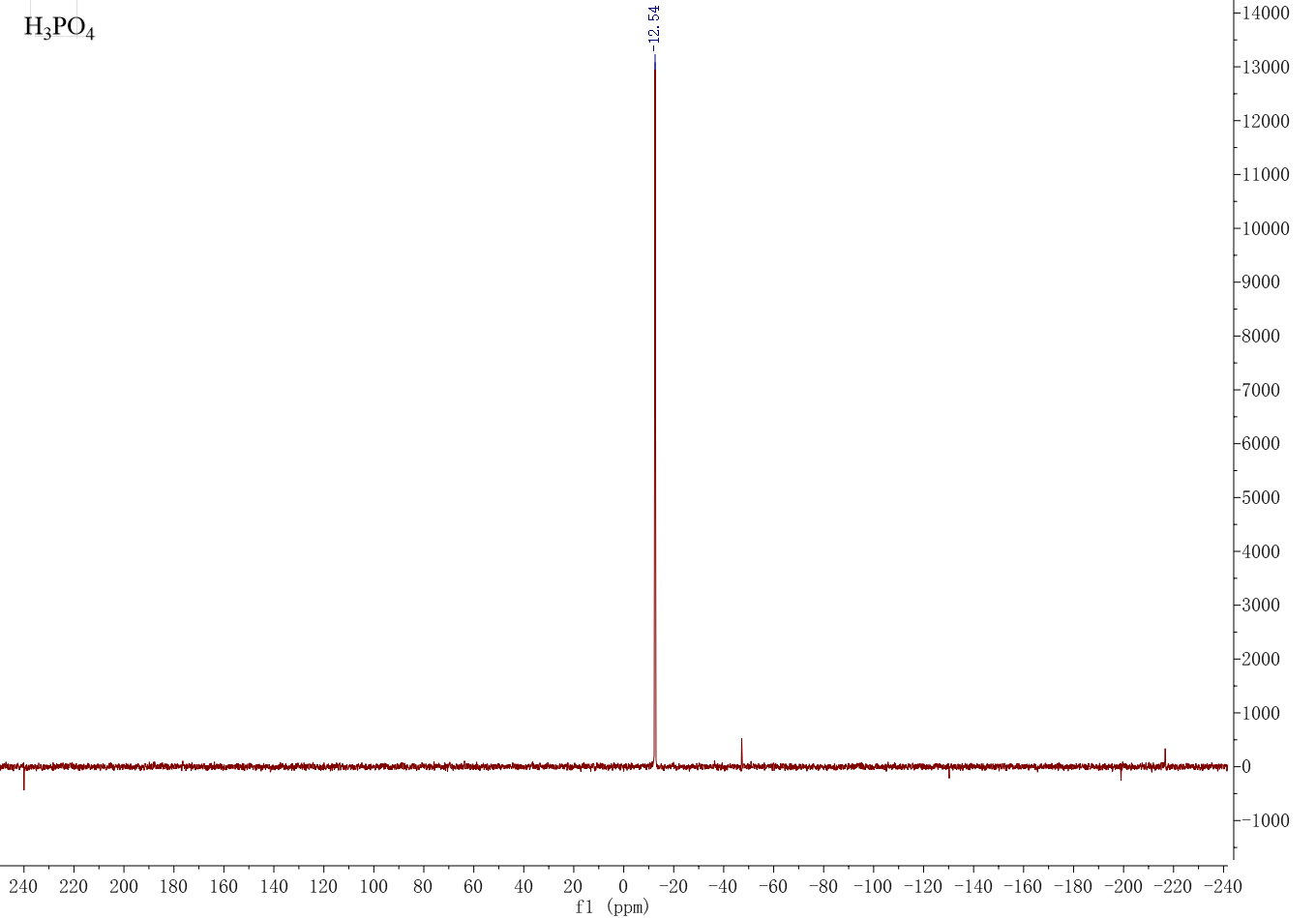


In the phosphorus spectrum, we used 85% phosphoric acid as an external standard.

Fig 52. *^31^P NMR of* **H_3_PO_4_** (162 MHz, DMSO) (pre-calibration)


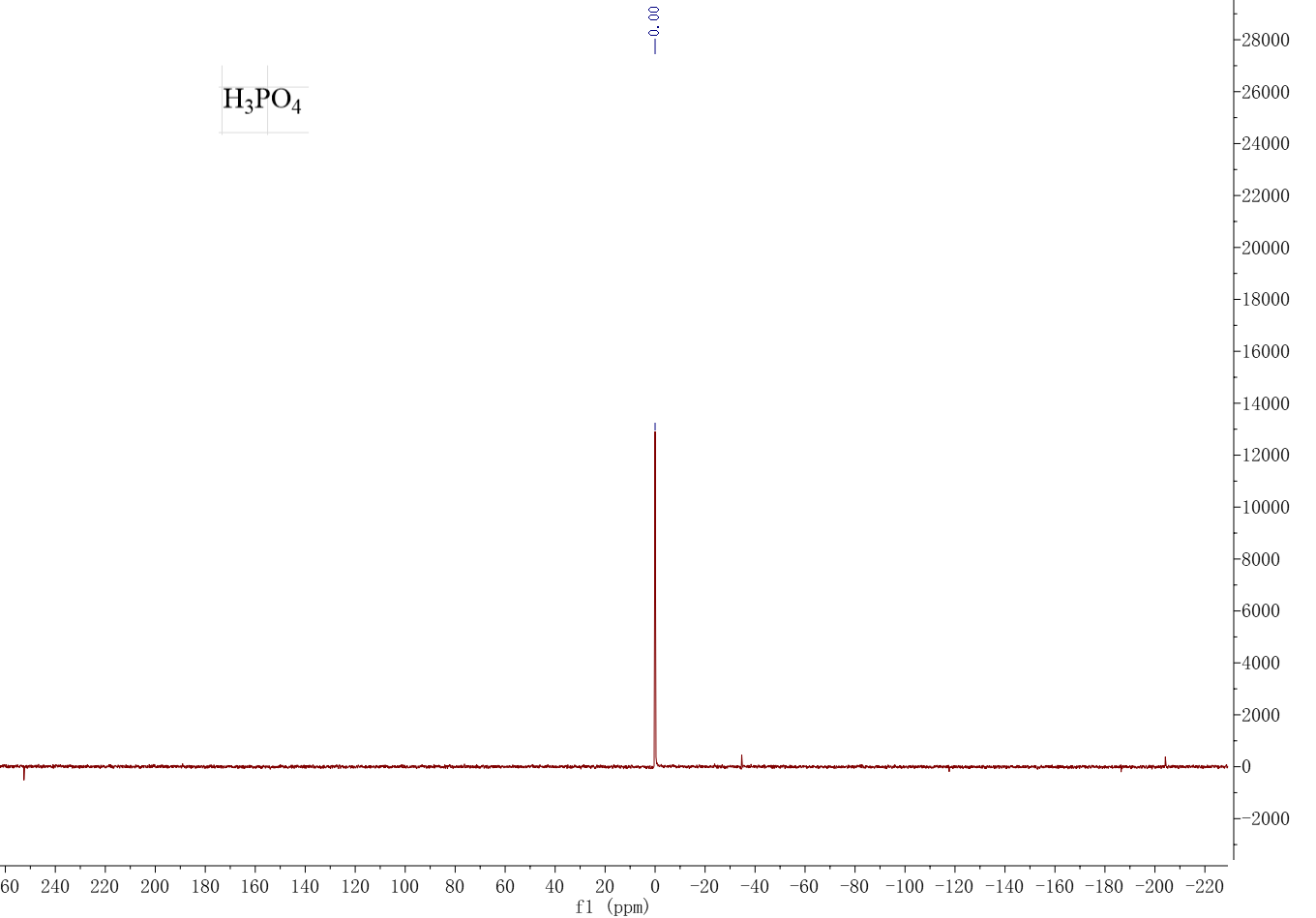


Fig 53. *^31^P NMR of* **H_3_PO_4_** (162 MHz, DMSO) (pre-calibration)


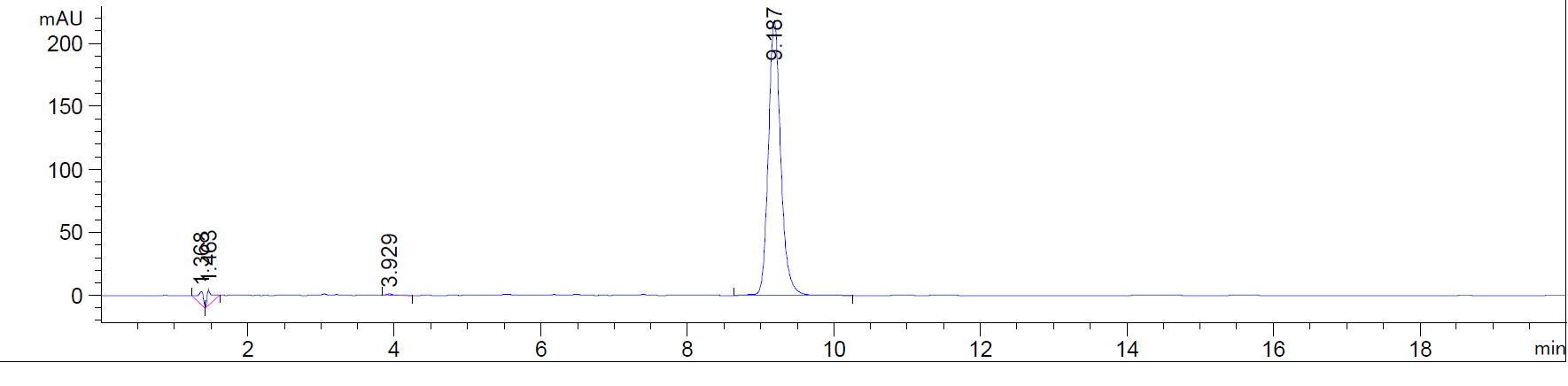

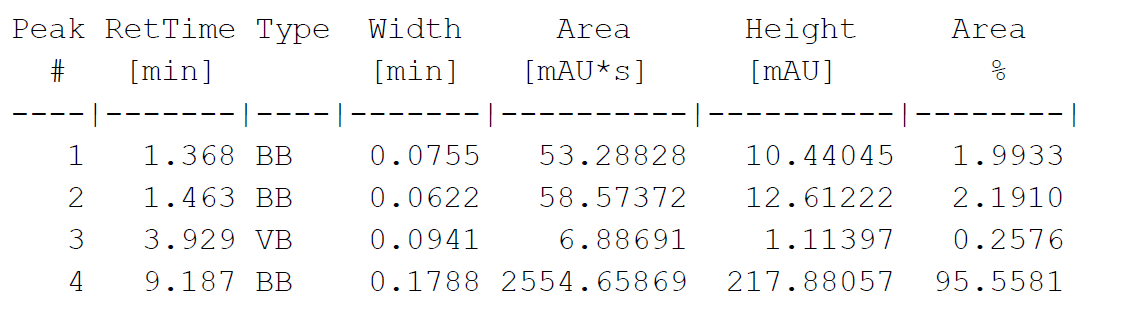


Fig 54. *HPLC chromatograms compound* ***S11****.*

Fig 55. *Mass spectrum of compound* ***S11***
